# The Relationship Between Physical Activity and Non-Modifiable Risk Factors on Alzheimer’s Disease and Brain Health Markers: A UK Biobank Study

**DOI:** 10.3233/JAD-240269

**Published:** 2024-10-08

**Authors:** Felicity S.E. Spencer, Richard J. Elsworthy, Leigh Breen, Jonathan Bishop, Sol Morrissey, Sarah Aldred

**Affiliations:** a School of Sport, Exercise and Rehabilitation Sciences, University of Birmingham, Birmingham, UK; b Birmingham Clinical Trials Unit, Institute of Applied Health Research, Public Health Building, College of Medical and Dental Sciences, University of Birmingham, Birmingham, UK; c Norwich Medical School, Faculty of Medicine and Health Sciences, University of East Anglia, Norwich, UK

**Keywords:** Alzheimer’s disease, apolipoprotein E4, brain, cognition, exercise, sex, UK Biobank

## Abstract

**Background:**

Modifiable (physical activity) and non-modifiable (sex and genotype) risk factors interact to affect Alzheimer’s disease (AD) risk. Further investigation is necessary to understand if these factors influence brain volume and cognition.

**Objective:**

The study aimed to assess the effect of physical activity, *APOE* genotype, and sex on AD risk, brain volume, and cognition.

**Methods:**

UK Biobank data from 2006 to 2023 was accessed. Physical activity was measured by accelerometers, and International Physical Activity Questionnaire. Outcomes were AD incidence; brain volume (ventricular cerebrospinal fluid and total brain); and cognition (executive function, memory, visuospatial ability, processing speed, and reaction time). Logistic and linear regression models were conducted.

**Results:**

69,060 participants met inclusion criteria (mean age: 62.28 years, SD: 7.84; 54.64% female). Higher self-reported (OR = 0.63, 95% CI [0.40, 1.00], *p* = 0.047) and accelerometer-assessed (OR = 0.96 [0.93, 0.98], *p* = 0.002) physical activity was associated with lower disease incidence. Smaller ventricular cerebrospinal fluid volume (β= – 65.43 [– 109.68, – 17.40], *p* = 0.007), and larger total brain volume (β= 4398.46 [165.11, 8631.82], *p* < 0.001) was associated with increased accelerometer-assessed and self-reported physical activity respectively. Both brain volume analyses were moderated by sex. Increased accelerometer-assessed physical activity levels were associated with faster reaction time (β= – 0.43 [– 0.68, – 0.18], *p* = 0.001); though poorer visuospatial ability (β= – 0.06 [– 0.09, – 0.03], *p* < 0.001), and executive function (β= 0.49 [0.31, 0.66], *p* < 0.001; β= 0.27 [0.10, 0.45], *p* = 0.002) was related to self-reported physical activity levels.

**Conclusions:**

Higher levels of physical activity reduce AD risk independently of non-modifiable risk factors. Moderation of sex on brain volume highlighted the importance of incorporating non-modifiable risk factors in analysis.

## BACKGROUND

Dementia is a neurodegenerative condition that affects cognitive functioning.^1^ Alzheimer’s disease (AD) is the most common form of dementia; accounting for 6-7 million new cases annually.^2^ AD incidence is expected to increase as the population ages, resulting in a growing burden on individuals affected by the disease and their carers, in addition to wider society and economics.^3^ Therefore, a better understanding of methods to prevent or delay the onset of AD is urgently needed.

Deterioration of brain structure and cognitive ability are typically evident decades before an AD diagnosis;^4,5^ and can be considered indicators of brain health. Specifically, accelerated shrinking of total brain volume and increasing ventricular volume can predict progression from mild cognitive impairment (MCI) to AD.^6^ Declines in cognitive domains, including executive function;^7^ memory;^8^ visuospatial ability;^9^ processing speed;^10^ and reaction time (RT)^11^ may accompany these structural changes.

Key non-modifiable risk factors for AD are sex, with women twice as likely as men to be diagnosed,^12^ and the Apolipoprotein (*APOE*) *ɛ*4 genotype. Homozygous carriers of *APOE4* have up to an 8-fold increased risk of developing AD.^13^ Interactions between sex and genetics substantially heighten AD risk for female carriers of *APOE4* (compared to non-carriers, females are 1.81× more likely to have AD, but males are only 1.27× more likely).^14^ Magnetic resonance imaging (MRI) scans show that *APOE4* carriers have reduced grey matter volume and larger ventricular cerebrospinal fluid (CSF) volume compared to non-carriers.^15^ Additionally, the *APOE4* genotype is associated with poorer cognitive abilities, including memory, and executive function.^16^

Approximately 40% of AD risk is the result of modifiable risk factors, including physical inactivity.^17^ Regular physical activity (PA) has a direct effect on neuronal health and cognition^18,19^ and has been associated with reduced AD risk;^20–22^ though there is not enough evidence to state causation. Systematic reviews^23,24^ have identified some benefits of exercise in randomized controlled trials (RCTs), compared to usual care or active control groups, for preventing dementia, but RCTs are typically conducted in small sample sizes (often underpowered)^25^ and with short intervention and follow-up periods; therefore, conclusive evidence for the benefits of physical activity is lacking.^26^ Additional research is needed to be able to better understand the relationship between daily PA and dementia accounting for non-modifiable risk factors, and thus to move towards evidence-based guidance on exercise prescription for dementia.

Higher levels of PA are associated with larger total brain volume^27^ and less self-reported cognitive decline^28^ in cross-sectional investigations, though this relationship can be mediated by non-modifiable risk factors. Low and high levels of PA have a greater effect on cognitive decline in *APOE4* carriers compared to non-carriers.^29,30^ Active women outperform active men in executive function tests.^31^ In mid-life, *APOE4* carriers may experience a decline in hippocampal volume with low PA, while non-carriers remain stable.^32^ Middle-aged females show increased brain volume with low-intensity PA compared to men.^31^ However, Brown et al. (2022)^33^ found that men with higher PA, showed increased grey matter and right hippocampal volume compared to women.

It’s likely that non-modifiable risk factors for AD interact with modifiable protective factors, such as PA, to amplify AD risk. Although RCTs have explored risk reduction via PA, few assess APOE genotype or disaggregate data by sex. Therefore, this observational study aims to better understand how the relationship between PA, AD incidence, brain volume, and cognitive function is influenced by sex and *APOE* genotype. This information may contribute towards the development of personalized guidance for a tailored PA program dependent on sex or *APOE* genotype.

## METHODS

This study follows Strengthening the Reporting of Observational Studies in Epidemiology (STROBE) guidelines.^34^ The protocol for this project was approved by the UK Biobank (project application number: 99415).

### Participants and setting

Participants were recruited by mail to the UK Biobank (UKB) from 2006– 2010 (instance 0: baseline).^35^ Invited participants were aged between 40– 69, lived within 25 miles of UKB assessment centers, and registered with the National Health Service (NHS). Ethical approval was granted by the NHS National Research Ethics Service. Participants supplied written consent.

### Exposure

International Physical Activity Questionnaire (IPAQ)^36^ was used to assess self-reported PA at baseline. The UKB categorized participants as having low, moderate, or high activity.

Objectively measured physical activity (accelerometer-assessed PA) was collected by wrist worn Axivity AX3 triaxial accelerometers worn continuously for seven days between February 2013 and December 2015.^37^ Average acceleration was recorded (milligravities; mg/week). For context, when walking at 1.6 mph, the average acceleration on wrist-worn accelerometers is 27.9 mg, and at 2.2 mph, it is 34.1 mg.^38^

*APOE* genotype was extracted from whole exome sequence data published in 2022 (see Supplementary Material 1 and Supplementary Table 1) and categorized as non-carrier/*APOE4* heterozygous/*APOE4* homozygous. Sex was self-reported at baseline.

### Primary outcome

AD incidence was established from linked NHS records until March 2023, regardless of diagnosis date.

### Cognitive outcomes

The following cognitive tests were completed at instance 2 (follow up) by touchscreen: Trail Making Test B (TMT-B) (participants sequentially pressed a series of digits and letters; executive function); numeric memory (participants recalled two digits, with the number of digits increasing by one after each successful recall, up to a maximum of 12; memory); pairs matching (participants matched cards shown briefly on the screen; the first round had 3 pairs of cards, and the second had 6; visuospatial ability); symbol digit substitution task (SDST) (participants inputted symbols into a grid, using a grid of corresponding symbols and letters as a guide; processing speed); and RT (participants were shown two cards simultaneously and pressed a button if they matched, for 12 rounds; RT).

### Neuroimaging outcomes

Full body MRI scans in a standard Siemans Kyra 3T scanner were completed at follow up.^39^ The UKB pre-processed data to calculate tissue volumes (mm^3^), including total brain volume (grey and white matter) and ventricular CSF volume.^39^

### Covariates

The following covariates were controlled for from follow up assessment: suspected Bipolar or depression; ethnicity (White/Asian/Black/Mixed); smoking; education (none/secondary/higher); cardiovascular disease; alcohol intake (none/rarely/frequently); sleep duration (<7 hours/7– 9 hours/>9 hours); diabetes; body mass index (underweight/healthy/overweight/obese/severely obese); social activity (none/rarely/frequently); Townsend Index of Multiple Deprivation Score (IMD); and age (calculated from date of birth to date of follow up visit) (see Supplementary Table 2 in Supplementary Material 1 for details of how these variables were generated from the UK Biobank database).

### Statistical analysis

An *a priori* power analysis was conducted using G*Power version 3.1.9.7^40^ for estimation of sample size; based on previously published UKB data on dementia risk reduction as a result of physical activity.^41^ It was calculated that the risk of dementia was reduced between 40– 84% depending on the type and frequency of physical activity conducted, compared to conducting less than the recommended amount of physical activity per week. An average value of 62% risk reduction was taken. Average acceleration was extracted, *M* = 28.23 (SD = 8.34) from Rowlands et al. (2021).^42^ With a Type 1 error rate of 5% and 80% power, the sample size needed was N = 27,431, to have a 5% chance of incorrectly rejecting the null hypothesis in detecting a 62% reduction in AD risk.

Analyses were conducted in R version 4.2.2; models were fitted using the base stats package. All tests were two-sided. Outliers were identified for accelerometer-assessed PA, executive function and RT using Tukey fences. Percentage correct scores were generated for the SDST, numeric memory and pairs matching tasks (see Supplementary Material 1 for further details). AD incidence was analyzed using logistic multivariable regression models, brain volume (total brain volume and ventricular CSF volume) and cognitive function (executive function, memory, processing speed, visuospatial ability, and RT) were analyzed using linear multivariable regression models. accelerometer-assessed PA and self-reported PA were analyzed in separate models. Each outcome was analyzed with two models. The first model included PA, *APOE* genotype, and sex; the second (full) added in all covariates. The reference values were as follows: sex (female); ethnicity (White); *APOE* genotype (non-carrier); IPAQ group (low); depression/bipolar diagnosis (no depression or single depressive episode only); smoking status (no); level of education (none); CVD diagnosis (no); diabetes diagnosis (no); frequency of alcohol intake (never); sleep duration (7– 9 hours); frequency of social visits (never) and BMI (healthy).

Interactions between PA, *APOE* genotype, and sex were considered in the first model alone, to avoid potential convergence issues in the full models. Interactions were examined by considering the *p* value for the overall interaction term; and, if significant, examining the individual level interaction terms in the model. Model assumptions were tested (see Supplementary Material 1). All variables were examined to determine whether assumptions were met, and if violated, the appropriate transformation was made to the variable. If assumption violations were unable to be corrected by transformation, then models that were robust to the assumption violations were also conducted.

As follow up times for AD incidence varied, AD incidence rates were estimated using Poisson regression models. Time to follow up (from baseline to AD diagnosis (or if no AD diagnosis, last data collection in March 2023)) (measured in days) was an offset term.

The relationship between accelerometer-assessed and self-reported physical activity was examined by Spearman’s rank correlation.

*Post-hoc* sensitivity analyses on models with significant findings were conducted to investigate the impact of missing data. Missing data was classified as any participant that would have otherwise been eligible for the study but was ineligible due to missing data for: suspected Bipolar or depression, smoking, alcohol intake, IMD, social activity, qualifications, diabetes, CVD, sleep duration, BMI, or ethnicity.

A multiple imputation approach, with best- and worst-case covariate data inputted with respect to Alzheimer’s Disease risk (see Supplementary Material 2) was used.

### Eligibility criteria

Participants were required to have all key predictor and covariate data available for the complete-case analysis (though additional inclusion criteria was applied separately for brain structure and cognition). The following exclusion criteria were applied: outlying accelerometer-assessed PA, or UKB indicated a problem with accelerometer-assessed PA data quality; diagnosis of AD prior to accelerometer-assessed PA collection (brain structure and cognition: follow up visit) or up to six weeks afterwards; accelerometer removed for >3 days; *ɛ*2/*ɛ*4 *APOE* genotype (as this genotype contains both risk reducing and risk increasing alleles); and outlying cognitive data.

## RESULTS

**Fig. 1:**
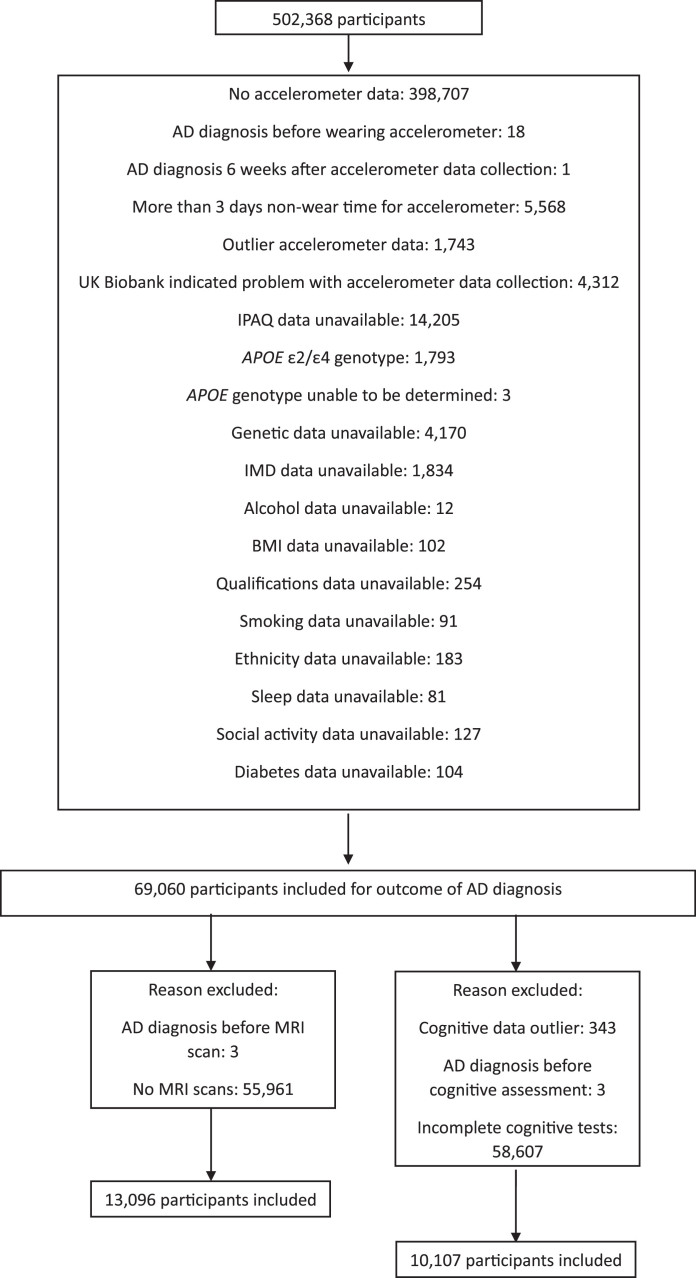
Exclusion criteria applied to the participants. Left: Outcomes of volume of ventricular CSF and total brain volume; Right: Outcomes of executive function; processing speed; memory; reaction time; and visuospatial ability.

69,060 participants (Mean age = 62.28 (SD: 7.84) years; 54.64% female) were eligible for inclusion in this study for the outcome of AD incidence; with 13096 (Mean age = 61.62 (7.62) years; 53.38% female) and 10107 (Mean age = 60.69 (7.53) years; 51.99% female) eligible for the outcomes of brain volume and cognition, respectively (Fig. 1).

Eligible participants were typically White (97.05% of total sample) and had received higher education (73.12% of total sample). Participants had an overall acceleration average of 27.59 (SD: 7.21), and most were classed as moderately active by the IPAQ (42.91% of total sample). These trends were reflected across the total sample of 69060 participants, and the subgroups for the outcomes of brain volume and cognition (Table 1).

**Table 1:** Descriptive statistics summarized by outcome

	Total AD incidence group (included participants) (N = 69060) N(%)	AD incidence (N = 69060) N(%)		Total MRI group (N = 13096) N(%)	Total cognitive function group (N = 10107) N(%)
		Yes (N = 141)	No (N = 68919)
**Sex**
Male	31326 (45.36)	79 (56.03)	31247 (45.34)	6105 (46.62)	4852 (48.01)
Female	37734 (54.64)	62 (43.97)	37672 (54.66)	6991 (53.38)	5255 (51.99)
**Ethnicity**
White	67022 (97.05)	136 (96.45)	66886 (97.05)	12787 (97.64)	9876 (97.71)
Asian	796 (1.15)	<5	793 (1.15)	132 (1.01)	100 (0.99)
Black	493 (0.71)	<5	492 (0.71)	58 (0.44)	37 (0.37)
Mixed	749 (1.08)	<5	748 (1.09)	119 (0.91)	94 (0.93)
**APOE genotype**
Non-carrier	50983 (73.82)	54 (38.30)	50929 (73.90)	9790 (74.76)	7550 (74.70)
Heterozygous carrier	16475 (23.86)	66 (46.81)	16409 (23.81)	3009 (22.98)	2334 (23.09)
Homozygous carrier	1602 (2.32)	21 (14.89)	1581 (2.29)	297 (2.27)	223 (2.21)
**Overall acceleration average (mg), Mean (SD)**	27.59 (7.21)	23.84 (7.37)	27.60 (7.21)	28.09 (7.07)	28.44 (7.05)
**IPAQ group**
Low	12330 (17.85)	31 (21.99)	12299 (17.85)	2369 (18.09)	1915 (18.95)
Moderate	27097 (42.91)	51 (36.17)	29582 (42.92)	5572 (42.55)	4355 (43.09)
High	29633 (39.24)	59 (41.84)	27038 (39.23)	5155 (39.36)	3837 (37.96)
**Age (at follow up), Mean (SD)**	62.28 (7.84)	70.13 (4.77)	62.26 (7.83)	61.62 (7.62)	60.69 (7.53)
**Depression/Bipolar status**
No depression or single depressive episode	64787 (93.81)	137 (97.16)	64650 (93.81)	12340 (94.23)	9529 (94.28)
Bipolar	204 (0.30)	<5	203 (0.29)	34 (0.26)	27 (0.27)
Recurrent depression	4069 (5.89)	<5	4066 (5.90)	722 (5.51)	551 (5.45)
**Smoking status**
No	26621 (38.55)	49 (34.75)	26572 (38.56)	5125 (39.13)	3973 (39.31)
Yes	42439 (61.45)	92 (65.25)	42347 (61.44)	7971 (60.87)	6134 (60.69)
**Level of education**
None	4862 (7.04)	29 (20.57)	4833 (7.01)	507 (3.87)	274 (2.71)
Secondary (GCSE/A level)	13703 (19.84)	28 (19.86)	13675 (19.84)	2054 (15.68)	1491 (14.75)
Higher (University degree/professional qualification)	50495 (73.12)	84 (59.57)	50411 (73.15)	10535 (80.44)	8342 (82.54)
**CVD diagnosis**
No	45123 (65.34)	76 (53.90)	45047 (65.36)	7840 (59.87)	6160 (60.95)
Yes	23937 (34.66)	65 (46.10)	23872 (34.64)	5256 (40.13)	3947 (39.05)
**Diabetes diagnosis**
No	66155 (95.79)	132 (93.62)	66023 (95.80)	12440 (95.00)	9613 (95.11)
Yes	2905 (4.21)	9 (6.38)	2896 (4.20)	656 (5.00)	494 (4.89)
**Frequency of alcohol intake**
None	4069 (5.89)	20 (14.18)	4049 (5.86)	799 (6.10)	605 (5.96)
Rarely (special occasions/1-3x per month)	14211 (20.58)	25 (17.73)	14186 (20.58)	2881 (22.00)	2169 (21.46)
Frequently (more than 1/2x per week)	50780 (73.53)	96 (68.09)	50684 (73.54)	9416 (71.90)	7333 (72.55)
**Sleep duration**
Less than 7 hours	15327 (22.19)	38 (26.95)	15289 (22.18)	3036 (23.18)	2333 (23.08)
7 – 9 hours	52897 (76.60)	102 (72.34)	52795 (76.60)	9892 (75.53)	7649 (75.68)
More than 9 hours	836 (1.21)	<5	836 (1.21)	168 (1.28)	125 (1.24)
**Social activity**
Never/no friends/family outside household	931 (1.35)	<5	928 (1.35)	176 (1.34)	129 (1.28)
Rarely (once every few months/once a month)	14994 (21.70)	31 (21.99)	14953 (21.70)	2606 (19.90)	2073 (20.51)
Frequently (more than once a week)	53145 (76.95)	107 (75.89)	53038 (76.96)	10314 (78.76)	7905 (78.21)
**BMI (kg/m^**2**^)**
Underweight (>18.5)	417 (0.60)	<5	417 (0.61)	100 (0.76)	88 (0.87)
Healthy (< = 18.5 & <25)	26852 (38.88)	56 (39.72)	26796 (38.88)	5471 (41.78)	4231 (41.86)
Overweight (< = 25 & <30)	28502 (41.27)	57 (40.43)	28445 (41.27)	5295 (40.43)	4073 (40.30)
Obese (< = 30 & <40)	12367 (17.91)	24 (17.02)	12343 (17.91)	2108 (16.10)	1610 (15.93)
Severely obese (< =40)	922 (1.34)	<5	918 (1.33)	122 (0.93)	105 (1.04)
**IMD**, Mean (SD)	14.59 (11.82)	15.51 (12.59)	14.59 (11.82)	14.73 (11.86)	13.93 (11.32)
**AD incidence**	141 (0.20)	141 (100.00)	68919 (0.00)	–	–
**Brain volume (mm^**3**^), Mean (SD)**
Volume of ventricular CSF	–	–	–	47437.29 (20523.04)	–
Total brain volume (grey and white matter)	–	–	–	1162172.00 (110413.10)	–
**Cognitive function, Mean (SD)**
Duration to complete TMT-B (s)	–	–	–	–	526.42 (164.69)
Reaction time (ms)	–	–	–	–	585.59 (89.72)
Pairs matching (% correct)	–	–	–	–	80.44 (11.38)
Numeric memory (% correct)	–	–	–	–	82.36 (6.25)
SDST (% correct)	–	–	–	–	95.77 (8.20)
**Time to follow up (years), Mean (SD)**	13.86 (0.84)	13.96 (0.84)	13.86 (0.84)	–	–

Note: Values given are N(%), unless otherwise stated in the row header. Note some values are reported as <5 due to UK Biobank reporting restrictions to preserve anonymity for small numbers.

There were 2,788 (4.04%) participants ineligible for analysis due to missing covariate data (see Supplementary Material 3; Supplementary Table 3). Excluded participants were more likely to be male, *χ*^2^(1) = 8.98, *p* = 0.003, and less likely to be White, *χ*^2^(3) = 47.88, *p* < 0.001, and have higher education, *χ*^2^(2) = 9.44, *p* = 0.009, than included participants (Supplementary Material 3; Supplementary Table 3). Excluded participants had lower average acceleration than included participants, t(71846) = 6.25, *p* < 0.001, but did not differ in distribution across IPAQ groups, *χ*^2^(2) = 5.69, *p* = 0.058.

There were 7,492 participants included in both the brain volume and cognition analyses. IMD score violated the assumptions of normal distribution and linear relationship with outcome (unimproved by transformation) and frequency of social activity violated the multicollinearity assumption. Therefore, both were removed from the full models. Time to complete the TMT-B underwent a square root transformation prior to analysis to correct the non-normal distribution. Percentage correct scores on the SDST, numeric memory, and pairs matching tasks were not normally distributed (unimproved by transformation). Consequently, Box-Cox (numeric memory) and Yeo-Johnson (SDST and pairs matching) transformed models were conducted, in addition to the uncorrected models (uncorrected models presented below as results unchanged for key predictors). For complete model statistics, see Supplementary Material 3 (Supplementary Tables 4–27).

**Fig. 2:**
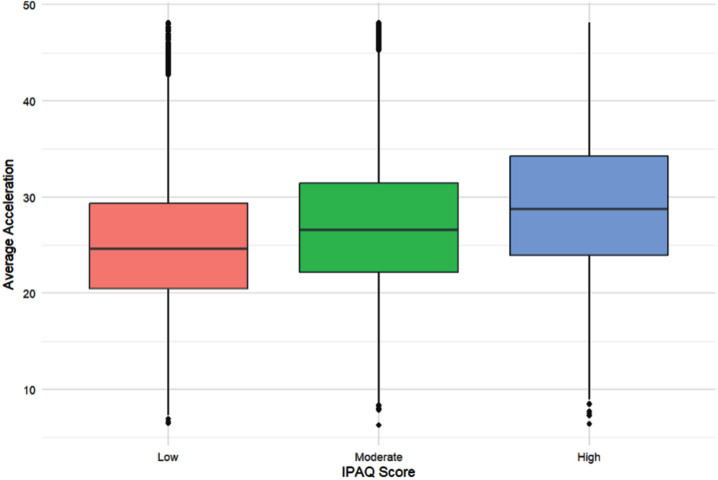
A boxplot of the relationship between average acceleration and IPAQ group. N = 69,060 participants (IPAQ low: 12,330; IPAQ moderate: 27,097; IPAQ high; 29,633). The red box represents people in the low IPAQ group; the green box people in the moderate IPAQ group; and the blue box those in the high IPAQ group. The line in the middle of the box represents the median acceleration (mg/week) for each group. The boxes represent the interquartile range (IQR) (first to third quartile (Q1 to Q3)); and the whiskers extend to Q1/Q3 – /+ 1.5 * the IQR.

Examination of the relationship between IPAQ score and acceleration revealed that Spearman’s rank correlation showed a weak positive relationship between all three groups: AD incidence, *r* = 0.20, *p* < 0.001, brain volume, *r* = 0.20, *p* < 0.001, and cognition, *r* = 0.21, *p* < 0.001 (Fig. 2).

**Fig. 3:**
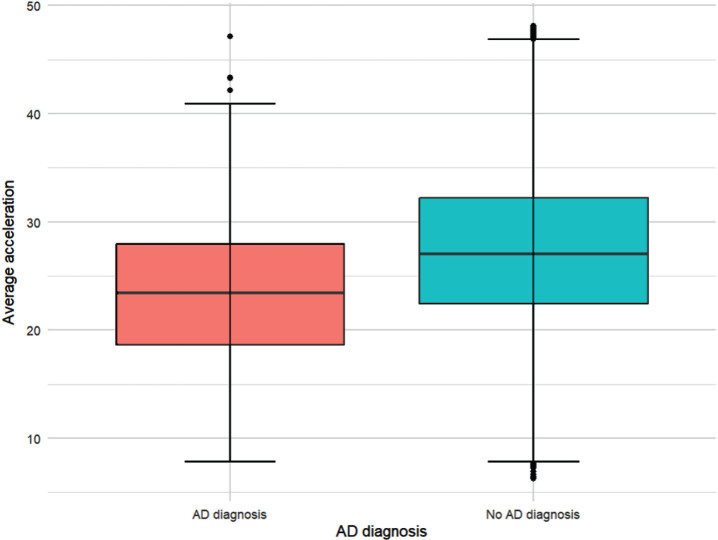
Average acceleration (mg/week) by AD diagnosis. N = 69,060 participants (141 with a diagnosis of AD; 68,919 without). The red box represents people with an AD diagnosis; and the blue box those without an AD diagnosis as of March 2023. The line in the middle of the box represents the median acceleration (mg/week) for each group. The boxes represent the interquartile range (IQR) (first to third quartile (Q1 to Q3)); and the whiskers extend to Q1/Q3 – /+ 1.5 * the IQR.

Higher levels of accelerometer-assessed PA were associated with a reduced risk of AD (OR [95% CI] = 0.96, [0.93, 0.98] *p* = 0.002) (Fig. 3).

The OR of 0.96 in the full model represents a 4% reduction in the odds of receiving an AD diagnosis with an increase of 1 mg weekly accelerometer-assessed PA (see Methods for interpretation of units). Higher levels of self-reported PA were also associated with reduced AD risk. In the full model, participants with moderate activity levels were less likely to have AD than those with low activity, OR [95% CI] = 0.63, [0.40, 1.00], *p* = 0.047. In all AD incidence full models, APOE4 genotype was associated with increased AD risk for heterozygous (OR [95% CI] = 4.21, [2.93, 6.07], *p* < 0.001 (e.g., accelerometer-assessed PA)) and homozygous (OR [95% CI] = 15.14, [8.84, 25.01], *p* < 0.001 (e.g., accelerometer-assessed PA)) carriers. Findings for key predictors remained consistent in the Poisson model for accelerometer-assessed PA (Supplementary Table 4), but self-reported PA became non-significant in the self-reported PA Poisson regression model (Supplementary Table 6).

**Fig. 4:**
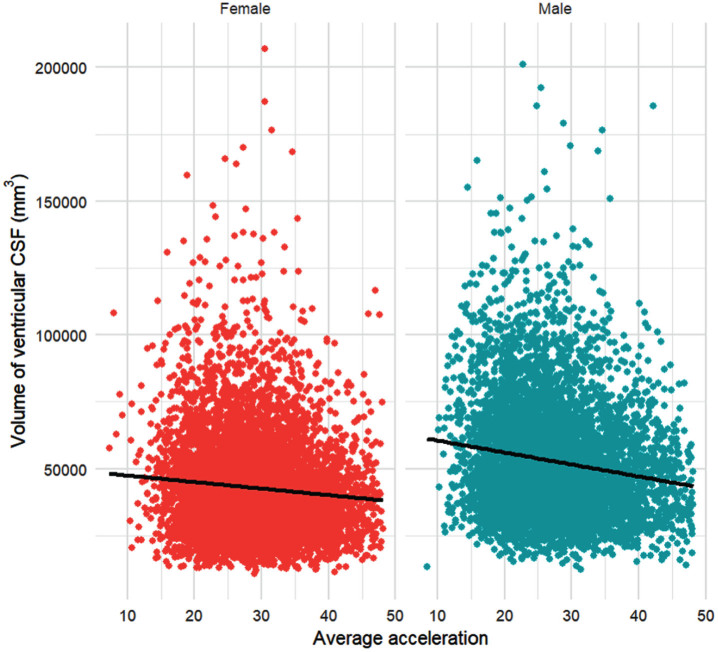
Average acceleration (mg/week) and volume of ventricular CSF (mm^3^), disaggregated by sex. N = 13,096 participants (6,105 male; 6,991 female). The red datapoints represent females, and the blue datapoints represent males. The lines of best fit represent the relationship between the volume of ventricular CSF (mm^3^) and average acceleration (mg/week).

When associations between PA and ventricular CSF volume were assessed, higher levels of accelerometer-assessed PA were associated with a smaller ventricular CSF volume, in the full, β [95% CI] = – 63.54, [– 109.68, – 17.40], *p* = 0.007 model. The full model demonstrates that for every 1 mg/week increase in accelerometer-assessed PA; the volume of ventricular CSF reduces by 63.54 mm^3^ on average. In the initial model, there was a significant interaction between PA and sex, β [95% CI] = – 216.80, [– 328.52, – 105.08], *p* < 0.001. Compared to females, males had a larger ventricular CSF volume, and as acceleration increased, males experienced a steeper decline in this volume (Fig. 4).

Higher self-reported PA was associated with a larger ventricular CSF volume in the initial model, β [95% CI] = 1581.75, [12.12, 3151.38], *p* = 0.048; though this was non-significant in the full model. There was no evidence of *APOE* genotype influencing ventricular CSF volume (e.g., from accelerometer-assessed PA model: APOE4 heterozygous: β [95% CI] = 355.91, [– 373.73, 1085.55], *p* = 0.339; *APOE4* homozygous: β [95% CI] = 722.92, [– 1337.00, 2782.83], *p* = 0.492.

There was little evidence of an effect of accelerometer-assessed PA on total brain volume; but those completing moderate levels of self-reported PA compared to those completing low levels had a larger total brain volume, β [95% CI] = 4398.46, [165.11, 8631.82], *p* = 0.042 in the full model. An interaction between IPAQ group and sex in the initial model β [95% CI] = 11430.15, [822.53, 22037.77], *p* = 0.035, occurred due to men having a larger brain volume on average. There was no evidence of an effect of APOE genotype on total brain volume in either the self-reported PA or accelerometer-assessed PA models (e.g., in the accelerometer-assessed PA model: APOE4 heterozygous: β [95% CI] = 1593.20, [– 1992.07, 5178.46], *p* = 0.384; APOE4 homozygous: β [95% CI] = – 527.74, [– 10649.64, 9594.17], *p* = 0.919.

There was little evidence of a relationship between accelerometer-assessed PA or self-reported PA alone on percentage correct scores on the SDST or the numeric memory task. Additionally, there was little evidence of an effect of *APOE* genotype on percentage correct scores on the SDST, numeric memory task, or pairs matching task, all *p* > 0.05 (see Supplementary Material).

There was a significant interaction between IPAQ-moderate and APOE4 heterozygous genotype on the numeric memory task, β [95% CI] = 1.13, [0.02, 2.25], *p* = 0.046; however, the higher-order interaction between self-reported PA and *APOE* genotype was non-significant, *p* = *0.*092, suggesting little evidence of an interaction here.

Increasing accelerometer-assessed PA was associated with a faster RT, β [95% CI] = – 0.43, [– 0.68, – 0.18], *p* = 0.001; modified by *APOE* genotype, β [95% CI] = 0.94, [0.14, 1.75], *p* = 0.021. The decreasing RT with increasing accelerometer-assessed PA was more gradual for homozygous *APOE4* carriers than non-carriers. self-reported PA was not associated with RT.

A longer duration to complete the TMT-B was associated with a higher self-reported PA group; high, β [95% CI] = 0.49, [0.31, 0.66], *p* < 0.001; moderate β [95% CI] = 0.27, [0.10, 0.45], *p* = 0.002, in the full model. The largest difference in mean duration to complete TMT-B was seen in high-activity *APOE4* homozygous males, β [95% CI] = 2.80, [0.31, 5.29], *p* = 0.028; although the interaction between sex, *APOE* genotype, and self-reported PA in the initial model showed little evidence of interaction here, (*p* = 0.201). TMT-B duration was unrelated to accelerometer-assessed PA.

Higher accelerometer-assessed PA was associated with lower scores on the pairs matching task, β [95% CI] = – 0.06, [– 0.09, – 0.03], *p* < 0.001. Although the interaction term between IPAQ-moderate and *APOE4* heterozygous carriers was significant, β [95% CI] = 2.13, [0.05, 4.20], *p* = 0.045, inspection of the higher-order interaction term demonstrated little evidence of interaction, *p* = 0.213.

*Post-hoc* sensitivity analyses showed that neither the best nor worst case scenarios affected the significance of any of the key predictors for the outcomes of AD incidence, volume of ventricular CSF, reaction time, or percentage correct on the pairs matching task (see Supplementary Material 2, Supplementary Tables 28–51). For the outcome of total brain volume, IPAQ group became unable to significantly predict this outcome in both the best, and worst, case models. Additionally, for duration to complete the TMT-B, IPAQ group remained significantly able to predict this in both best- and worst-case models; but average acceleration was also able to predict mean duration (higher acceleration associated with longer duration to complete the task).

## DISCUSSION

This study examined the relationship between modifiable and non-modifiable risk factors for AD, and their effect on AD incidence and brain health. Increasing accelerometer-assessed PA and self-reported PA levels were associated with decreased AD risk and improvements in brain structure. PA showed some relationship with cognition; but findings were less clear than for other outcomes.

Accelerometer-assessed PA and self-reported PA predicted AD independently. This suggests that regardless of non-modifiable (sex and *APOE* genotype) and modifiable risk factor (health and lifestyle) status, increasing PA levels decreases the risk of AD. Only 141/69,060 people had received a diagnosis of AD at the time of publication, demonstrating the strength of PA as a predictor for AD. This supports the position that physical inactivity is a key modifiable risk factor for AD.

As accelerometer-assessed PA increased, the volume of ventricular CSF decreased. An increase in ventricular CSF volume is a key predictor of AD;^6^ therefore, this represents an additional mechanism through which PA may contribute to reduced AD risk. The preservation of ventricular volume may stem from improved maintenance of white and grey matter volume with increasing accelerometer-assessed PA.^27,43^ Sex-specific exercise preferences may influence the interaction between sex and accelerometer-assessed PA. Women typically focus more on lower body and weight loss exercises, while men prioritize enjoyment and muscle gain.^44^ This result aligns with findings that PA correlates with brain volume in men alone.^33^ The larger decrease in ventricular CSF volume with increasing PA in men may be partly attributed to better-preserved grey matter volume.

Moderate self-reported PA was associated with larger total brain volume compared to low activity. This somewhat aligns with findings that PA is linearly related to brain volume.^27,45,46^ However, brain volume was unaffected by accelerometer-assessed PA, and there was no difference between high and low IPAQ groups. Varied exercise intensities can contribute to brain volume maintenance,^45^ so it is possible that the typical intensity of PA performed was insufficient to alter brain volume. The interaction between self-reported PA and sex was the result of men having on average, larger brain volume than women; both showed similar trends.

Increasing accelerometer-assessed PA was associated with faster RT. Performing PA provides an opportunity to quicken responses and train cognitive skills that contribute to improved reaction times.^47^ Slower RT is associated with MCI and AD,^48,49^ but the mechanism linking this is unclear.^48^ Faster RT in older adults is associated with reduced fall risk,^50^ demonstrating the importance of PA in this population.

Surprisingly, compared to low activity, moderate and high self-reported PA levels were associated with a slower duration to complete the TMT-B. All combinations of sex and *APOE* genotype took longer to complete the TMT-B with increasing activity, except for homozygous *APOE4* females. This contrasts with the finding that high levels of PA can mitigate accelerated cognitive decline in *APOE4* carriers;^51^ though the small number of homozygous *APOE4* participants here may influence the findings.

Increasing accelerometer-assessed PA was associated with worse visuospatial ability. However, further inspection of the data revealed that the difference between average percentage correct score on the pairs matching task between participants with low and high acceleration values was negligible. Neither processing speed nor memory were predicted by accelerometer-assessed PA or self-reported PA alone. Both have been associated with PA;^52–55^ though the null findings here may be the result of ceiling effects, as participants achieved high percentage correct scores.

Accelerometer-assessed PA and self-reported PA were well-matched, suggesting that responses were reliable. Despite this, they did not always predict the same outcomes. This may be as the IPAQ uses categories whereas accelerometer data records activity continuously. accelerometer-assessed PA and self-reported PA correspond well at the population level, however, on a case-by-case basis there may be discrepancies between them, leading to the disparity between the models.

This study had some key strengths. Firstly, the assessment of accelerometer-assessed PA and self-reported PA allowed for a better understanding of the relationship between both kinds of PA and the outcomes measured. Additionally, the large number of participants included in the study enabled the investigation of the relationship between PA and non-modifiable risk factors for AD. This demonstrated that there was some interaction between the two risk factor types, with possible implications for tailored physical activity interventions. Finally, the variables for this research were collected over a period of up to 17 years, allowing long-term associations between PA, and AD incidence, brain volume, and cognition to be ascertained.

Despite the strengths of this study, some limitations were present. Firstly, though numerous lifestyle covariates were included to account for relationships between these variables and PA; diet was not, as diet measurement in the UKB is complex and multi-faceted.^56^ Furthermore, causal relationships between variables cannot be drawn from the regression models used. Due to a small number of recorded AD diagnoses and large number of included covariates in the dataset, more appropriate time-to-event models would not converge so simpler regression models were used. Generalizability of the findings is limited by the lack of ethnic diversity in the UKB. Though the dataset was representative of the UK population at creation; it is now biased towards White participants.^57^

This study demonstrated that physical activity is related to AD incidence, brain volume, and cognition. Regardless of genetics and lifestyle factors, higher levels of PA can reduce AD risk. The relationship between physical activity and cognition was less clear here, though did suggest a role for physical activity in faster reaction times.

Finally, sex did mediate the relationship between physical activity and volume of ventricular cerebrospinal fluid; highlighting the importance of incorporating non-modifiable risk factors into data analysis to better understand how physical activity contributes to reduced Alzheimer’s disease risk. Future research could explore the relationship between PA and these outcomes in more diverse participants.

## AUTHOR CONTRIBUTIONS

Felicity Spencer (Conceptualization; Data curation; Formal analysis; Methodology; Software; Visualization; Writing – original draft; Writing – review & editing); Richard Jonathan Elsworthy (Conceptualization; Methodology; Supervision; Writing – review & editing); Leigh Breen (Supervision; Writing – review & editing); Jonathan Bishop (Conceptualization; Data curation; Formal analysis; Methodology; Supervision; Validation; Writing – review & editing); Sol Morrissey (Conceptualization; Formal analysis; Methodology; Writing – review & editing); Sarah Aldred (Conceptualization; Methodology; Supervision; Writing – review & editing).

## Supplementary Material

Supplementary Material 1

Supplementary Material 2

Supplementary Material 3

Supplementary Material 4

## Data Availability

The data that support the findings of this study are available from the UK Biobank, but restrictions apply to the availability of these data, which were used under license (project number: 99415) for the current study, and so are not publicly available. Data are however available from the UK Biobank upon reasonable request.
