# Supplementary material for: The Relationship Between Physical Activity and Non-Modifiable Risk Factors on Alzheimer’s Disease and Brain Health Markers: A UK Biobank Study
Source: J Alzheimers Dis. 2024 Oct 8;101(4):1029–42. doi: 10.3233/JAD-240269 (PMC11492105; doi:10.3233/JAD-240269)
Supplement: Supplementary Material 1 [file jad-101-jad240269-s001.docx]

**Method**

**Generating APOE genotype**

Data on APOE genotype was obtained from whole exome sequence data on chromosome 19 on the UK Biobank research analysis platform. The swiss army knife tool version 4.9.1 was used to generate a list of participants that had withdrawn, which were removed from the extracted data. A file was generated with the location of rs7412 and rs429358 on chromosome 19 (19 44908822 44908822; 19 44908684 44908684) and homozygous ref/alt/heterozygous status was extracted from these locations. Ref for rs7412 is C; and for rs429358 it is T.

APOE4 genotype was generated according to this table.^1^

Supplementary Table 1: APOE genotype and alleles at the corresponding rsIDs.

| APOE genotype | rs429358 | rs7412 |
| --- | --- | --- |
| ε2ε2 | (T;T) | (T;T) |
| ε2ε3 | (T;T) | (C;T) |
| ε2ε4 | (C;T) | (C;T) |
| ε3ε3 | (T;T) | (C;C) |
| ε3ε4 | (C;T) | (C;C) |
| ε4ε4 | (C;C) | (C;C) |

APOE genotype was categorised into homozygous carrier (ε4/ε4); heterozygous carrier (ε3/ε4), and non-carrier (ε2/ε2, ε2/ε3, and ε3/ε3). Participants with the ε2/ε4 genotype were excluded.

**Generation of cognitive scores**

For analysis of the cognitive data, percentage correct scores were generated for the numeric memory, pairs matching and SDST. For numeric memory and SDST, percentage correct was generated by dividing the number of correct rounds (SDST: matches) by the number of rounds (SDST: matches) attempted and multiplying by 100. For pairs matching the number of correct matches was divided by the sum of the number of correct and incorrect matches for each round (round one and two separately) and multiplying by 100, before averaging the two percentage scores to generate an overall percentage correct score for the two rounds. Round one and two alone were used as the UK Biobank reported that data from round three did not add any additional information about participant performance (UK Biobank, 2023).

Supplementary Table 2: Variables extracted from the UK Biobank database, and transformation applied prior to analysis

| **Variable Code** | **Variable name**  **(All recorded at instance 2 alone unless otherwise stated)** | **Variable categories** | **Variable categories transformation** |
| --- | --- | --- | --- |
| 90012 | Overall acceleration average | Continuous | Outliers removed |
| 90002 | Data problem indicator | Dataset previously flagged as unreliable, now believed valid; unreliable due to unexpectedly small size; unreliable due to unexpectedly large size | Matched with 90012 to remove any values from 90012 that met any of these categories |
| 90010 | Start time of wear | Continuous (date) |  |
| 22032 | IPAQ activity group (Instance 0) | Low; Moderate; High |  |
| 131036 | Date G30 first reported (Alzheimer’s disease) (March 2023) | Continuous (date) | Transformed from date of diagnosis to a yes/no outcome; and compared to ‘start time to wear’ to establish if a diagnosis of AD occurred before or up to six weeks after accelerometer data collection |
| 6350 | Duration to complete alphanumeric path (trail #2) | Continuous, Trail not completed | Outliers removed, participants with trail not completed excluded |
| 23323 | Number of symbol digit matches attempted | Continuous | Combined to create a % correct score for this task |
| 23324 | Number of symbol digit matches made correctly | Continuous |  |
| 398 | Number of correct matches in round (Array 1 and 2) | Continuous | % correct score for each array generated and then average across arrays |
| 399 | Number of incorrect matches in round (Array 1 and 2) | Continuous |  |
| 4282 | Maximum digits remembered correctly | Continuous, Abandoned | Combined to create a % correct score for this task. Participants who abandoned were excluded. |
| 4283 | Number of rounds of numeric memory test performed | Continuous |  |
| 20023 | Mean time to correctly identify matches | Continuous | Outliers removed |
| 25009 | Volume of brain, grey+white matter (normalised for head size) | Continuous |  |
| 25003 | Volume of ventricular cerebrospinal fluid (normalised for head size) | Continuous |  |
| 20126 | Bipolar and major depression status | No Bipolar or Depression; Bipolar I Disorder; Bipolar II Disorder; Probable Recurrent major depression (severe); Probable Recurrent major depression (moderate); Single Probable major depression episode | Merged as follows:  1 (No Bipolar or Recurrent Depression) – No Bipolar or Depression and Single Probable major depression episode or blank  2 (Bipolar) – Bipolar I Disorder and Bipolar II Disorder  3 (Depression) – Probable Recurrent major depression (severe) and Probable Recurrent major depression (moderate) |
| 21000 | Ethnic background (Instances 0-2) | White; British; Irish; Any other white background; Mixed; White and Black Caribbean; White and Black African; White and Asian; Any other mixed background; Asian or Asian British; Indian; Pakistani; Bangladeshi; Any other Asian background; Black or Black British; Caribbean; African; Any other Black background; Chinese; Other ethnic group; Do not know; Prefer not to answer | Merged as follows across instances:  1 (White) – White, British, Irish, Any other white background  2 (Asian) – Asian or Asian British, Indian, Pakistani, Bangladeshi, Any other Asian background, Chinese  3 (Black) – Black or Black British, Caribbean, African, Any other Black background  4 (Mixed) – Any other ethnic background, or if a combination of categories 1/2/3 was given across instances 0-2  Only include participants in categories 1, 2, 3 and 4. Participants recording prefer not to answer were excluded |
| 20160 | Ever smoked (Instances 0-2) | Yes; No | If Yes was given at any instance then it was considered that the participant had smoked |
| 6138 | Qualifications (Instances 0-2) | College or University degree; A levels/AS levels or equivalent; O levels/GCSEs or equivalent; CSEs or equivalent; NVQ or HND or HNC or equivalent; Other professional qualifications, e.g., nursing, teaching; None of the above; Prefer not to answer | The highest level of reported qualification at any timepoint was used to create the below categories.  Merged as follows:  1 (None) – None of the above  2 (Secondary) – A levels/AS levels or equivalent; O levels/GCSEs or equivalent; CSEs or equivalent  3 (Higher & Vocational) – College or University degree; Other professional qualifications, e.g., nursing, teaching; NVQ or HND or HNC or equivalent  Participants recording prefer not to answer were excluded |
| 20002 | Non-cancer illness code, self-reported (Instances 0-2) |  | CVD extracted, by search for the terms "hypertension", "essential hypertension", "gestational hypertension/pre-eclampsia", "heart/cardiac problem", "angina", "heart attack/myocardial infarction", "heart failure/pulmonary oedema", "heart arrhythmia", "atrial fibrillation", "atrial flutter", "wolff parkinson white / wpw syndrome", "irregular heart beat", "sick sinus syndrome", "svt / supraventricular tachycardia", "heart valve problem/heart murmur", "mitral valve disease", "mitral valve prolapse", "mitral stenosis", "mitral regurgitation / incompetence", "aortic valve disease", "aortic stenosis", "aortic regurgitation / incompetence", "cardiomyopathy", "hypertrophic cardiomyopathy (hcm / hocm)", "pericardial problem", "pericarditis", "pericardial effusion", "myocarditis", "rheumatic fever", "cerebrovascular disease", "stroke", "subarachnoid haemorrhage", "brain haemorrhage", "ischaemic stroke", "transient ischaemic attack (tia)", "subdural haemorrhage/haematoma", "cerebral aneurysm", "peripheral vascular disease"," leg claudication/ intermittent claudication", "arterial embolism", "aortic aneurysm", "aortic aneurysm rupture", "aortic dissection", "venous thromboembolic disease", "pulmonary embolism +/- dvt", "deep venous thrombosis (dvt)", "high cholesterol", "other venous/lymphatic disease", "varicose veins", "lymphoedema" and "varicose ulcer", if reported at any instance a diagnosis of CVD was recorded. |
| 1558 | Alcohol intake frequency (Instances 0-2) | Prefer not to answer; Never; Special occasions only; One to three times a month; Once or twice a week; Three or four times a week; Daily or almost daily | If instance 2 was available, this value was used as the final value. If not, then the value from instance 1 was used, and so on.  Merged as follows:  1 (None) – Never  2 (Rarely) – Special occasions only; One to three times a month  3 (Frequently) – Once or twice a week; Three or four times a week; Daily or almost daily  Participants recording prefer not to answer were excluded |
| 1160 | Sleep duration (Instances 0-2) | Continuous; Prefer not to answer; Do not know | If instance 2 was available, this value was used as the final value. If not, then the value from instance 1 was used, and so on.  Merged as follows:  1 – Less than 7 hours per night  2 – 7–9 hours per night  3 – More than 9 hours per night  Participants recording prefer not to answer or do not know were excluded |
| 2443 | Diabetes diagnosed by doctor (Instances 0-2) | Yes; No; Do not know; Prefer not to answer | If Yes was given at any instance then it was considered that the participant had received a diagnosis of diabetes from a doctor  Participants recording prefer not to answer or do not know were excluded |
| 21001 | Body mass index (BMI) (Instances 0-2) | Continuous | If instance 2 was available, this value was used as the final value. If not, then the value from instance 1 was used, and so on.  Merged as follows:  1 (Underweight) – BMI less than 18.5  2 (Healthy weight) – BMI more than or equal to 18.5 but less than 25  3 (Overweight) – BMI more than or equal to 25 but less than 30  4 (Obese) – BMI more than or equal to 30 but less than 40  5 (Severely obese) – BMI more than or equal to 40 |
| 1031 | Frequency of friend/family visits (Instances 0-2) | Almost daily; 2-4 times a week; About once a week; About once a month; Once every few months; Never or almost never; No friends/family outside household; Do not know; Prefer not to answer | If instance 2 was available, this value was used as the final value. If not, then the value from instance 1 was used, and so on.  Merged as follows:  1 (None) – Never or almost never; No friends/family outside household  2 (Rarely) – About once a month; Once every few months  3 (Frequently) – Almost daily; 2-4 times a week; About once a week  Participants recording prefer not to answer or do not know were excluded |
| 26410 | Index of Multiple Deprivation (England) (Instance 0) | Continuous | Scores were merged. Participants without available data were excluded. |
| 26427 | Index of Multiple Deprivation (Scotland) (Instance 0) | Continuous |  |
| 26426 | Index of Multiple Deprivation (Wales) (Instance 0) | Continuous |  |
| 34 | Year of birth (Instance 0) | Continuous (date) | Used to calculate age at instance 2 |
| 31 | Sex (Instance 0) | Female; Male |  |

**Assumptions testing**

Assumptions testing for the logistic regression models was completed for the assumptions of having, no influential observations measured by Cook’s distance (defined as a score of Cook’s distance >1), and no high intercorrelation (multicollinearity) between the predictors examined by variance inflation factor (VIF). High multicollinearity was defined as a score of VIF >10. Residuals were examined with plots of residual vs. fitted values, and QQ plots. The assumption of linearity between the logit of the outcome and predictor was tested for continuous predictors only.

Assumptions testing for the linear regression models examined whether there was a linear relationship between a functional form of the outcome and predictor by scatterplot; if residuals of included variables were normally distributed by histogram; that multicollinearity (examined by VIF; VIF >10 indicates high multicollinearity) and auto-correlation (examined by non-constant error variance; p<0.05 indicates high auto-correlation) between the variables was not present, and whether there was homoscedasticity (examined by the Durbin Watson test; p<0.05 indicates heteroscedasticity).

**REFERENCES**

1. Li M, Zhao JV, Kwok MK et al. Age and sex specific effects of APOE genotypes on ischemic heart disease and its risk factors in the UK Biobank. *Sci Reports* 2021;11: 9229.
