# Supplementary material for: The Relationship Between Physical Activity and Non-Modifiable Risk Factors on Alzheimer’s Disease and Brain Health Markers: A UK Biobank Study
Source: J Alzheimers Dis. 2024 Oct 8;101(4):1029–42. doi: 10.3233/JAD-240269 (PMC11492105; doi:10.3233/JAD-240269)
Supplement: Supplementary Material 2 [file jad-101-jad240269-s002.docx]

**Sensitivity Analysis Model Coefficients**

The following variables were entered into the best case model: smoking (non-smoker) ^1^; ethnicity (White) ^2^; education (higher) ^3^; CVD (no) ^1^; alcohol intake (rare) ^4^; sleep duration (7-9 hours) ^5^; diabetes (no) ^1^; BMI (healthy range) ^6^; depression/bipolar (no depression) ^7^.

The following variables were entered into the worst case model: smoking (smoker) ^1^; ethnicity (Black) ^2^; education (none) ^3^; CVD (yes) ^1^; alcohol intake (frequently) ^4^; sleep duration (less than 7 hours) ^5^; diabetes (yes) ^1^; BMI (severely obese) ^8^; depression/bipolar (recurrent depression) ^7^.

Supplementary Table 28. Results from the logistic regression sensitivity analysis where AD incidence was modelled with average acceleration; ‘worst case’ data was imputed for missing covariates.

|  | Model one | | | Model two | | | |
| --- | --- | --- | --- | --- | --- | --- | --- |
|  | Estimate | Odds ratio  [95% CI] | p | | Estimate | Odds ratio  [95% CI] | p |
| Average acceleration | -010 [-0.16, -0.04] | 0.91 [0.85, 0.96] | **0.003** | | -0.04 [-0.07, -0.01] | 0.96 [0.94, 0.99] | **0.004** |
| Sex | -0.76 [-0.16, -0.04] | 0.47 [0.06, 3.65] | 0.466 | | 0.25 [-0.10, 0.60] | 1.28 [0.91, 1.82] | 0.163 |
| APOE4 heterozygous | 1.31 [-0.79, 3.43] | 3.69 [0.45, 30.81] | 0.225 | | 1.44 [1.09, 1.81] | 4.24 [2.97, 6.09] | **<0.001** |
| APOE4 homozygous | 2.92 [-0.01, 5.78] | 18.60 [0.99, 323.70] | **0.048** | | 2.78 [2.27, 3.27] | 16.17 [9.64, 26.29] | **<0.001** |
| Average acceleration*Sex | 0.05 [-0.04, 0.13] | 1.05 [0.96, 1.14] | 0.276 | |  |  |  |
| Average acceleration*APOE4 heterozygous | 0.00 [-0.08, 0.09] | 1.00 [0.92, 1.09] | 0.954 | |  |  |  |
| Average acceleration* APOE4 homozygous | -0.01 [-0.12, 0.11] | 0.99 [0.88, 1.11] | 0.931 | |  |  |  |
| Sex * APOE4 heterozygous | 0.22 [-2.54, 2.97] | 1.24 [0.08, 19.42] | 0.877 | |  |  |  |
| Sex * APOE4 homozygous | 0.49 [-3.33, 4.31] | 1.63 [0.04, 74.21] | 0.803 | |  |  |  |
| Average acceleration * Sex * APOE4 heterozygous | -0.01 [-0.12, 0.10] | 0.99 [0.89, 1.11] | 0.876 | |  |  |  |
| Average acceleration * Sex * APOE4 homozygous | -0.03 [-0.19, 0.12] | 0.97 [0.83, 1.13] | 0.678 | |  |  |  |
| Bipolar |  |  |  | | 0.94 [-1.97, 2.54] | 2.56 [0.14, 12.71] | 0.369 |
| Recurrent depression |  |  |  | | -0.74 [-2.14, 0.24] | 0.48 [0.12, 1.27] | 0.208 |
| Ethnicity Asian |  |  |  | | 1.09 [-0.34, 2.11] | 2.96 [0.71, 8.28] | 0.072 |
| Ethnicity Black |  |  |  | | 0.58 [-2.31, 2.13] | 1.78 [ 0.10, 8.45] | 0.573 |
| Ethnicity Mixed |  |  |  | | 0.24 [-2.64, 1.75] | 1.27 [0.07, 5.78] | 0.815 |
| Has smoked |  |  |  | | 0.10 [-0.25, 0.46] | 1.10 [ 0.78, 1.58] | 0.594 |
| Secondary education |  |  |  | | -0.52 [-1.05, 0.01] | 0.60 [0.35, 1.01] | 0.055 |
| Higher education |  |  |  | | -0.65 [-1.07, -0.20] | 0.52 [ 0.34, 0.82] | **<0.001** |
| CVD diagnosis |  |  |  | | -0.13 [-0.48, 0.21] | 0.88 [0.62, 1.24] | 0.460 |
| Rare alcohol intake |  |  |  | | -0.89 [-1.47, -0.29] | 0.41 [0.23, 0.75] | **<0.001** |
| Frequent alcohol intake |  |  |  | | -0.89 [-1.37, -0.36] | 0.41 [0.25, 0.69] | **<0.001** |
| Sleep less than 7 hours |  |  |  | | 0.28 [-0.10, 0.64] | 1.32 [0.90, 1.90] | 0.142 |
| Sleep more than 9 hours |  |  |  | | -0.94 [-3.82, 0.57] | 0.39 [0.02, 1.78] | 0.351 |
| Diabetes diagnosis |  |  |  | | -0.07 [-0.85, 0.59] | 0.93 [0.43, 1.80] | 0.845 |
| BMI obese |  |  |  | | -0.41 [-0.93, 0.08] | 0.66 [0.39, 1.09] | 0.113 |
| BMI overweight |  |  |  | | -0.36 [-0.74, 0.01] | 0.70 [0.48, 1.01] | 0.058 |
| BMI severe obese |  |  |  | | 0.57 [-0.67, 1.54] | 1.78 [0.51, 4.69] | 0.298 |
| BMI underweight |  |  |  | | -12.10 [-120.00, -9.77] | 0.00 [0.00, 0.00] | 0.965 |
| Age |  |  |  | | 0.19 [0.15, 0.22] | 1.21 [1.16, 1.25] | **<0.001** |

Supplementary Table 29. Results from the logistic regression sensitivity analysis where AD incidence was modelled with IPAQ group; ‘worst case’ data was imputed for missing covariates.

|  | Model one | | | | Model two | | |  |
| --- | --- | --- | --- | --- | --- | --- | --- | --- |
|  | Estimate | Odds ratio | p | Estimate | | Odds ratio | p | |
| IPAQ high | -0.07 [-1.02, 0.98] | 0.93 [0.36, 2.68] | 0.887 | -0.27 [-0.70, 0.19] | | 0.77 [0.50, 1.20] | 0.236 | |
| IPAQ moderate | -0.95 [-2.11, 0.22] | 0.39 [0.12, 1.24] | 0.102 | -0.46 [-0.91, 0.00] | | 0.63 [0.40, 1.00] | **0.047** | |
| Sex | 0.63 [-0.36, 1.71] | 1.88 [0.70, 5.52] | 0.224 | 0.27 [-0.08, 0.62] | | 1.31 [0.92, 1.86] | 0.133 | |
| APOE4 heterozygous | 1.30 [0.19, 2.43] | 3.65 [1.21, 11.37] | **0.020** | 1.44 [1.08, 1.80] | | 4.22 [2.96, 6.06] | **<0.001** | |
| APOE4 homozygous | 1.82 [-1.12, 3.60] | 6.18 [0.33, 36.49] | 0.093 | 2.78 [2.26, 3.26] | | 16.10 [9.60, 26.17] | **<0.001** | |
| IPAQ high*Sex | -0.55 [-1.12, 3.60] | 0.58 [0.15, 2.08] | 0.408 |  | |  |  | |
| IPAQ moderate*Sex | 0.06 [-1.88, 0.73] | 1.06 [0.24, 4.56] | 0.940 |  | |  |  | |
| IPAQ high * APOE4 heterozygous | -0.21 [-1.41, 1.52] | 0.81 [0.20, 3.16] | 0.763 |  | |  |  | |
| IPAQ moderate * APOE4 heterozygous | 0.45 [-1.59, 1.15] | 1.56 [0.35, 7.07] | 0.554 |  | |  |  | |
| IPAQ high * APOE4 homozygous | 0.74 [-1.04, 1.96] | 2.09 [0.25, 45.14] | 0.541 |  | |  |  | |
| IPAQ moderate * APOE4 homozygous | 1.51 [-1.38, 3.81] | 4.52 [0.51, 101.71] | 0.225 |  | |  |  | |
| Sex * APOE4 heterozygous | -0.65 [-0.68, 4.62] | 0.52 [0.11, 2.30] | 0.396 |  | |  |  | |
| Sex * APOE4 homozygous | -0.65 [-2.18, 0.84] | 0.52 [0.02, 14.86] | 0.663 |  | |  |  | |
| IPAQ high * Sex * APOE4 heterozygous | 0.95 [-4.02, 2.70] | 2.59 [0.41, 17.06] | 0.315 |  | |  |  | |
| IPAQ moderate * Sex * APOE4 heterozygous | 0.69 [-0.89, 2.84] | 1.99 [0.28, 14.49] | 0.494 |  | |  |  | |
| IPAQ high * Sex * APOE4 homozygous | 1.00 [-2.59, 4.60] | 2.72 [0.08, 99.39] | 0.550 |  | |  |  | |
| IPAQ moderate * Sex * APOE4 homozygous | -0.02 [-3.71, 3.67] | 0.99 [0.02, 38.42] | 0.993 |  | |  |  | |
| Bipolar |  |  |  | 0.98 [-1.94, 2.58] | | 2.65 [0.14, 13.26] | 0.353 | |
| Recurrent depression |  |  |  | -0.73 [-2.13, 0.25] | | 0.48 [0.12, 1.29] | 0.216 | |
| Ethnicity Asian |  |  |  | 1.08 [-0.35, 2.11] | | 2.95 [0.71, 8.28] | 0.074 | |
| Ethnicity Black |  |  |  | 0.55 [-2.34, 2.10] | | 1.73 [0.10, 8.16] | 0.593 | |
| Ethnicity Mixed |  |  |  | 0.20 [-2.68, 1.71] | | 1.21 [0.07, 5.55] | 0.847 | |
| Has smoked |  |  |  | 0.11 [-0.25, 0.47] | | 1.11 [0.78, 1.59] | 0.567 | |
| Secondary education |  |  |  | -0.52 [-1.05, 0.01] | | 0.60 [0.35, 1.01] | 0.055 | |
| Higher education |  |  |  | -0.63 [-1.05, -0.18] | | 0.53 [0.35, 0.84] | **0.004** | |
| CVD diagnosis |  |  |  | -0.09 [-0.44, 0.25] | | 0.91 [0.64, 1.29] | 0.598 | |
| Rare alcohol intake |  |  |  | -0.86 [-1.45, -0.27] | | 0.42 [0.23, 0.77] | **0.004** | |
| Frequent alcohol intake |  |  |  | -0.89 [-1.37, -0.36] | | 0.41 [0.25, 0.70] | **<0.001** | |
| Sleep less than 7 hours |  |  |  | 0.26 [-0.12, 0.62] | | 1.30 [0.89, 1.86] | 0.170 | |
| Sleep more than 9 hours |  |  |  | -0.90 [-3.77, 0.62] | | 0.41 [0.02, 1.86] | 0.373 | |
| Diabetes diagnosis |  |  |  | -0.02 [-0.80, 0.64] | | 0.98 [0.45, 1.89] | 0.958 | |
| BMI obese |  |  |  | -0.27 [-0.78, 0.21] | | 0.76 [0.46, 1.24] | 0.288 | |
| BMI overweight |  |  |  | -0.29 [-0.66, 0.08] | | 0.75 [0.52, 1.09] | 0.128 | |
| BMI severe obese |  |  |  | 0.82 [-0.41, 1.77] | | 2.27 [0.66, 5.89] | 0.131 | |
| BMI underweight |  |  |  | -12.20 [-119.00, -9.69] | | 0.00 [0.00, 0.00] | 0.964 | |
| Age |  |  |  | 0.20 [0.16, 0.24] | | 1.22 [1.18, 1.27] | <**0.001** | |

Supplementary Table 30. Results from the logistic regression sensitivity analysis where AD incidence was modelled with average acceleration; ‘best case’ data was imputed for missing covariates.

|  | Model one | | | | Model two | | |  |
| --- | --- | --- | --- | --- | --- | --- | --- | --- |
|  | Estimate | Odds ratio [95% CI] | p | Estimate | | Odds ratio [95% CI] | p | |
| Average acceleration | -010 [-0.16, -0.04] | 0.91 [0.85, 0.96] | **0.003** | -0.04 [-0.07, -0.01] | | 0.96 [0.94, 0.99] | **0.004** | |
| Sex | -0.76 [-0.16, -0.04] | 0.47 [0.06, 3.65] | 0.466 | 0.26 [-0.10, 0.60] | | 1.30 [0.92, 1.84] | 0.143 | |
| APOE4 heterozygous | 1.31 [-0.79, 3.43] | 3.69 [0.45, 30.81] | 0.225 | 1.44 [1.09, 1.81] | | 4.24 [2.97, 6.09] | **<0.001** | |
| APOE4 homozygous | 2.92 [-0.01, 5.78] | 18.60 [0.99, 323.70] | **0.048** | 2.79 [2.27, 3.27] | | 16.24 [9.68, 26.40] | **<0.001** | |
| Average acceleration*Sex | 0.05 [-0.04, 0.13] | 1.05 [0.96, 1.14] | 0.276 |  | |  |  | |
| Average acceleration*APOE4 heterozygous | 0.00 [-0.08, 0.09] | 1.00 [0.92, 1.09] | 0.954 |  | |  |  | |
| Average acceleration* APOE4 homozygous | -0.01 [-0.12, 0.11] | 0.99 [0.88, 1.11] | 0.931 |  | |  |  | |
| Sex * APOE4 heterozygous | 0.22 [-2.54, 2.97] | 1.24 [0.08, 19.42] | 0.877 |  | |  |  | |
| Sex * APOE4 homozygous | 0.49 [-3.33, 4.31] | 1.63 [0.04, 74.21] | 0.803 |  | |  |  | |
| Average acceleration * Sex * APOE4 heterozygous | -0.01 [-0.12, 0.10] | 0.99 [0.89, 1.11] | 0.876 |  | |  |  | |
| Average acceleration * Sex * APOE4 homozygous | -0.03 [-0.19, 0.12] | 0.97 [0.83, 1.13] | 0.678 |  | |  |  | |
| Bipolar |  |  |  | 0.99 [-1.94, 2.63] | | 2.68 [0.14, 13.88] | 0.351 | |
| Recurrent depression |  |  |  | 0.03 [-0.36, 0.45] | | 1.03 [0.70, 1.56] | 0.894 | |
| Ethnicity Asian |  |  |  | 1.09 [-0.34, 2.12] | | 2.97 [0.71, 8.35] | 0.073 | |
| Ethnicity Black |  |  |  | 0.70 [-1.11, 1.88] | | 2.02 [ 0.33, 6.56] | 0.331 | |
| Ethnicity Mixed |  |  |  | 0.20 [-2.68, 1.72] | | 1.22 [0.07, 5.56] | 0.844 | |
| Has smoked |  |  |  | 0.08 [-0.27, 0.44] | | 1.08 [ 0.76, 1.56] | 0.655 | |
| Secondary education |  |  |  | -0.51 [-1.04, 0.02] | | 0.60 [0.35, 1.02] | 0.059 | |
| Higher education |  |  |  | -0.64 [-1.05, -0.19] | | 0.53 [ 0.35, 0.82] | **<0.001** | |
| CVD diagnosis |  |  |  | -0.14 [-0.48, 0.21] | | 0.87 [0.62, 1.23] | 0.441 | |
| Rare alcohol intake |  |  |  | -0.89 [-1.47, -0.29] | | 0.41 [0.23, 0.75] | **<0.001** | |
| Frequent alcohol intake |  |  |  | -0.88 [-1.36, -0.36] | | 0.41 [0.26, 0.70] | **<0.001** | |
| Sleep less than 7 hours |  |  |  | 0.27 [-0.11, 0.63] | | 1.31 [0.89, 1.88] | 0.156 | |
| Sleep more than 9 hours |  |  |  | -0.96 [-3.83, 0.59] | | 0.38 [0.02, 1.76] | 0.345 | |
| Diabetes diagnosis |  |  |  | -0.07 [-0.85, 0.59] | | 0.93 [0.43, 1.80] | 0.842 | |
| BMI obese |  |  |  | -0.41 [-0.93, 0.09] | | 0.67 [0.39, 1.09] | 0.117 | |
| BMI overweight |  |  |  | -0.35 [-0.73, 0.03] | | 0.70 [0.48, 1.03] | 0.068 | |
| BMI severe obese |  |  |  | 0.63 [-0.45, 1.53] | | 1.90 [0.63, 4.60] | 0.194 | |
| BMI underweight |  |  |  | -12.10 [-120.00, -14.10] | | 0.00 [0.00, 0.00] | 0.965 | |
| Age |  |  |  | 0.19 [0.15, 0.23] | | 1.21 [1.17, 1.25] | **<0.001** | |

Supplementary Table 31. Results from the logistic regression sensitivity analysis where AD incidence was modelled with IPAQ group; ‘best case’ data was imputed for missing covariates.

|  | Model one | | | | Model two | | |
| --- | --- | --- | --- | --- | --- | --- | --- |
|  | Estimate | Odds ratio | p | Estimate | | Odds ratio | p |
| IPAQ high | -0.07 [-1.02, 0.98] | 0.93 [0.36, 2.68] | 0.887 | -0.27 [-0.70, 0.19] | | 0.77 [0.50, 1.20] | 0.236 |
| IPAQ moderate | -0.95 [-2.11, 0.22] | 0.39 [0.12, 1.24] | 0.102 | -0.46 [-0.90, 0.01] | | 0.63 [0.41, 1.01] | **0.049** |
| Sex | 0.63 [-0.36, 1.71] | 1.88 [0.70, 5.52] | 0.224 | 0.28 [-0.07, 0.63] | | 1.32 [0.93, 6.06] | 0.117 |
| APOE4 heterozygous | 1.30 [0.19, 2.43] | 3.65 [1.21, 11.37] | **0.020** | 1.44 [1.08, 1.80] | | 4.22 [2.96, 6.06] | <**0.001** |
| APOE4 homozygous | 1.82 [-1.12, 3.60] | 6.18 [0.33, 36.49] | 0.093 | 2.78 [2.27, 3.27] | | 16.15 [9.63, 26.25] | <**0.001** |
| IPAQ high*Sex | -0.55 [-1.12, 3.60] | 0.58 [0.15, 2.08] | 0.408 |  | |  |  |
| IPAQ moderate*Sex | 0.06 [-1.88, 0.73] | 1.06 [0.24, 4.56] | 0.940 |  | |  |  |
| IPAQ high * APOE4 heterozygous | -0.21 [-1.41, 1.52] | 0.81 [0.20, 3.16] | 0.763 |  | |  |  |
| IPAQ moderate * APOE4 heterozygous | 0.45 [-1.59, 1.15] | 1.56 [0.35, 7.07] | 0.554 |  | |  |  |
| IPAQ high * APOE4 homozygous | 0.74 [-1.04, 1.96] | 2.09 [0.25, 45.14] | 0.541 |  | |  |  |
| IPAQ moderate * APOE4 homozygous | 1.51 [-1.38, 3.81] | 4.52 [0.51, 101.71] | 0.225 |  | |  |  |
| Sex * APOE4 heterozygous | -0.65 [-0.68, 4.62] | 0.52 [0.11, 2.30] | 0.396 |  | |  |  |
| Sex * APOE4 homozygous | -0.65 [-2.18, 0.84] | 0.52 [0.02, 14.86] | 0.663 |  | |  |  |
| IPAQ high * Sex * APOE4 heterozygous | 0.95 [-4.02, 2.70] | 2.59 [0.41, 17.06] | 0.315 |  | |  |  |
| IPAQ moderate * Sex * APOE4 heterozygous | 0.69 [-0.89, 2.84] | 1.99 [0.28, 14.49] | 0.494 |  | |  |  |
| IPAQ high * Sex * APOE4 homozygous | 1.00 [-2.59, 4.60] | 2.72 [0.08, 99.39] | 0.550 |  | |  |  |
| IPAQ moderate * Sex * APOE4 homozygous | -0.02 [-3.71, 3.67] | 0.99 [0.02, 38.42] | 0.993 |  | |  |  |
| Bipolar |  |  |  | 1.02 [-1.90, 2.67] | | 2.78 [0.15, 14.48] | 0.353 |
| Recurrent depression |  |  |  | 0.03 [-0.36, 0.45] | | 1.03 [0.70, 1.57] | 0.878 |
| Ethnicity Asian |  |  |  | 1.09 [-0.34, 2.13] | | 2.98 [0.71, 8.38] | 0.073 |
| Ethnicity Black |  |  |  | 0.68 [-1.14, 1.85] | | 1.97 [0.32, 6.36] | 0.350 |
| Ethnicity Mixed |  |  |  | 0.16 [-2.71, 1.68] | | 1.18 [0.07, 5.36] | 0.873 |
| Has smoked |  |  |  | 0.09 [-0.26, 0.45] | | 1.09 [0.77, 1.57] | 0.620 |
| Secondary education |  |  |  | -0.51 [-1.04, 0.02] | | 0.60 [0.35, 1.02] | 0.058 |
| Higher education |  |  |  | -0.62 [-1.04, -0.18] | | 0.54 [0.35, 0.84] | **0.004** |
| CVD diagnosis |  |  |  | -0.10 [-0.44, 0.25] | | 0.91 [0.64, 1.28] | 0.578 |
| Rare alcohol intake |  |  |  | -0.86 [-1.45, -0.26] | | 0.42 [0.24, 0.77] | **0.004** |
| Frequent alcohol intake |  |  |  | -0.88 [-1.36, -0.36] | | 0.41 [0.26, 0.70] | **<0.001** |
| Sleep less than 7 hours |  |  |  | 0.25 [-0.13, 0.61] | | 1.29 [0.88, 1.85] | 0.184 |
| Sleep more than 9 hours |  |  |  | -0.91 [-3.79, 0.61] | | 0.40 [0.02, 1.83] | 0.367 |
| Diabetes diagnosis |  |  |  | -0.02 [-0.80, 0.64] | | 1.29 [0.88, 1.85] | 0.958 |
| BMI obese |  |  |  | -0.27 [-0.78, 0.21] | | 0.98 [0.45, 1.89] | 0.287 |
| BMI overweight |  |  |  | -0.28 [-0.66, 0.09] | | 0.76 [0.46, 1.24] | 0.141 |
| BMI severe obese |  |  |  | 0.85 [-0.24, 1.72] | | 2.34 [0.79, 5.56] | 0.082 |
| BMI underweight |  |  |  | -12.20 [-125.00, 116.00] | | 0.00 [0.00, 0.00] | 0.965 |
| Age |  |  |  | 0.20 [0.17, 0.24] | | 1.22 [1.18, 1.27] | **<0.001** |

Supplementary Table 32. Results from the sensitivity analysis linear regression where volume of ventricular CSF was modelled by average acceleration, ‘best case’ data was imputed for missing covariates.

|  | **Model one** | | **Model two** | |
| --- | --- | --- | --- | --- |
|  | **Estimate** | **p** | **Estimate** | **p** |
| Average acceleration | -270.85 [-348.49, -192.30] | **<0.001** | -69.19 [-114.60, -23.79] | **0.003** |
| Sex | 15004.86 [11842.67, 18167.05] | **<0.001** | 7175.16 [6544.64, 7805.68] | **<0.001** |
| APOE4 heterozygous | -1145.21 [-5693.24, 3402.82] | 0.622 | 368.74 [-348.55, 1086.03] | 0.314 |
| APOE4 homozygous | -7079.87 [-20923.63, 6763.89] | 0.316 | 442.20 [-1597.22, 2481.61] | 0.671 |
| Average acceleration*Sex | -194.42 [-304.24, -84.60] | **<0.001** |  |  |
| Average acceleration*APOE4 heterozygous | 51.81 [-101.21, 204.83] | 0.507 |  |  |
| Average acceleration* APOE4 homozygous | 205.37 [-258.34, 669.08] | 0.385 |  |  |
| Sex * APOE4 heterozygous | -1872.95 [-8377.02, 4631.13] | 0.572 |  |  |
| Sex * APOE4 homozygous | 7849.62 [-11616.82, 27316.07] | 0.429 |  |  |
| Average acceleration * Sex * APOE4 heterozygous | 30.41 [-193.24, 254.06] | 0.790 |  |  |
| Average acceleration * Sex * APOE4 homozygous | -181.63 [-842.63, 379.38] | 0.590 |  |  |
| Bipolar |  |  | -773.76 [-6607.15, 5059.64] | 0.795 |
| Recurrent depression |  |  | 68.13 [-1395.32, 1259.05] | 0.920 |
| Ethnicity Asian |  |  | -1844.35 [-4847.78, 1159.07] | 0.229 |
| Ethnicity Black |  |  | -6281.96 [-10750.54, -1813.38] | **0.006** |
| Ethnicity Mixed |  |  | 271.12 [-2839.70, 3381.93] | 0.864 |
| Has smoked |  |  | 461.30 [-162.24, 1084.84] | 0.147 |
| Secondary education |  |  | 1076.43 [-643.48, 2796.34] | 0.220 |
| Higher education |  |  | 2425.80 [843.15, 4008.45] | **0.003** |
| CVD diagnosis |  |  | 1171.67 [510.56, 1832.78] | **<0.001** |
| Rare alcohol intake |  |  | -1426.21 [-2798.89, -53.52] | **0.020** |
| Frequent alcohol intake |  |  | -101.90 [-1382.60, 1178.79] | 0.876 |
| Sleep less than 7 hours |  |  | -3.80 [-717.01, 709.42] | 0.992 |
| Sleep more than 9 hours |  |  | 1768.66 [-926.81, 4464.13] | 0.198 |
| Diabetes diagnosis |  |  | 3466.36 [2053.37, 4879.35] | **<.001** |
| BMI obese |  |  | -102.25 [-1031.04, 826.54] | 0.829 |
| BMI overweight |  |  | -554.57 [-1236.29,127.15] | 0.111 |
| BMI severe obese |  |  | 2247.98 [-946.13, 5442.10] | 0.168 |
| BMI underweight |  |  | -608.70 [-4058.29 2840.90] | 0.729 |
| Age |  |  | 1133.05 [1090.04, 1176.06] | **<0.001** |

Supplementary Table 33. Results from the sensitivity analysis linear regression where volume of ventricular CSF was modelled by IPAQ group, ‘best case’ data was imputed for missing covariates.

|  | Model one | | Model two | |  |
| --- | --- | --- | --- | --- | --- |
|  | Estimate | p | Estimate | p | |
| IPAQ high | 1604.37 [61.65, 3147.10] | **0.042** | 852.74 [-7.05, 1712.52] | 0.052 | |
| IPAQ moderate | 1117.52 [-387.60, 2622.64] | 0.146 | 793.64 [-54.12, 1641.39] | 0.067 | |
| Sex | 9317.67 [7502.30, 11133.05] | **<0.001** | 7213.41 [6582.66, 7844.16] | **<0.001** | |
| APOE4 heterozygous | -661.14 [-3257.71, 1935.43] | 0.618 | 342.45 [-375.03, 1059.93] | 0.350 | |
| APOE4 homozygous | -2236.77 [-9858.96, 5385.43] | 0.565 | 411.96 [-1627.88, 2451.79] | 0.692 | |
| IPAQ high*Sex | 366.14 [-1835.28, 2567.56] | 0.744 |  |  | |
| IPAQ moderate*Sex | 1230.37 [-940.11, 3400.85] | 0.267 |  |  | |
| IPAQ high * APOE4 heterozygous | 1658.11 [-1461.55, 4777.78] | 0.298 |  |  | |
| IPAQ moderate * APOE4 heterozygous | 442.41 [-2637.38, 3522.20] | 0.778 |  |  | |
| IPAQ high * APOE4 homozygous | 1573.32 [-7583.56, 10730.19] | 0.736 |  |  | |
| IPAQ moderate * APOE4 homozygous | 774.06 [-8117.95, 9666.07] | 0.865 |  |  | |
| Sex * APOE4 heterozygous | -54.50 [-3890.23, 3781.24] | 0.978 |  |  | |
| Sex * APOE4 homozygous | 5127.37 [-5471.95, 15726.70] | 0.343 |  |  | |
| IPAQ high * Sex * APOE4 heterozygous | -1242.20 [-5833.89, 3349.48] | 0.596 |  |  | |
| IPAQ moderate * Sex * APOE4 heterozygous | -1300.42 [-5870.23, 3269.38] | 0.577 |  |  | |
| IPAQ high * Sex * APOE4 homozygous | -1106.09 [-13833.80, 11621.62] | 0.865 |  |  | |
| IPAQ moderate * Sex * APOE4 homozygous | -6937.26 [-19897.86, 6023.24] | 0.294 |  |  | |
| Bipolar |  |  | -829.67 [-6664.34, 5004.99] | 0.780 | |
| Recurrent depression |  |  | -8.10 [-1334.87, 1318.66] | 0.990 | |
| Ethnicity Asian |  |  | -1893.53 [-4897.83, 1110.77] | 0.217 | |
| Ethnicity Black |  |  | -6260.48 [-10730.11, -1790.85] | **0.006** | |
| Ethnicity Mixed |  |  | 250.51 [-2861.35, 3362.37] | 0.875 | |
| Has smoked |  |  | 464.91 [-158.80, 1088.62] | 0.144 | |
| Secondary education |  |  | 1149.42 [-571.41, 2870.25] | 0.190 | |
| Higher education |  |  | 2475.07 [890.90, 4059.24] | **0.002** | |
| CVD diagnosis |  |  | 1222.30 [561.46, 1883.14] | **<0.001** | |
| Rare alcohol intake |  |  | -1389.14 [-2762.14, -16.13] | **0.047** | |
| Frequent alcohol intake |  |  | -145.14 [-1425.99, 1135.70] | 0.824 | |
| Sleep less than 7 hours |  |  | -7.80 [-721.38, 705.79] | 0.983 | |
| Sleep more than 9 hours |  |  | 1949.53 [-745.48, 4644.54] | 0.156 | |
| Diabetes diagnosis |  |  | 3663.12 [2251.00, 5075.24] | **<0.001** | |
| BMI obese |  |  | 244.87 [-668.05, 1157.79] | 0.559 | |
| BMI overweight |  |  | -397.59 [-1074.46, 279.29] | 0.250 | |
| BMI severe obese |  |  | 2957.93 [-4217.48, 6133.34] | 0.068 | |
| BMI underweight |  |  | -697.11 [-4146.82, 2752.61] | 0.692 | |
| Age |  |  | 1144.55 [1102.44, 1186.66] | **<0.001** | |

Supplementary Table 34. Results from the sensitivity analysis linear regression where total brain volume was modelled by average acceleration, ‘best case’ data was imputed for missing covariates.

|  | Model one | | Model two | |
| --- | --- | --- | --- | --- |
|  | Estimate | p | Estimate | p |
| Average acceleration | 709.68 [339.56, 1079.80] | .**<0.001** | -84.04 [-307.17, 139.09] | 0.460 |
| Sex | 101891.79 [86817.93, 116965.65] | **<0.001** | 123371.36 [120272.83, 126469.89] | **<0.001** |
| APOE4 heterozygous | -24052.32 [-45732.35, -2372.29] | **0.030** | 1015.31 [-2509.65, 4540.26] | 0.572 |
| APOE4 homozygous | -1277.29 [-67269.22, 64714.64] | 0.970 | -141.35 [-10163.57, 9880.87] | 0.978 |
| Average acceleration*Sex | 433.19 [-90.31, 956.69] | 0.105 |  |  |
| Average acceleration*APOE4 heterozygous | 798.79 [69.37, 1528.21] | **0.032** |  |  |
| Average acceleration* APOE4 homozygous | 145.84 [-2064.62, 2356.31] | 0.897 |  |  |
| Sex * APOE4 heterozygous | 38281.37 [7277.05, 69285.69] | **0.016** |  |  |
| Sex * APOE4 homozygous | 55608.82 [-37185.91,148403.56] | 0.240 |  |  |
| Average acceleration * Sex * APOE4 heterozygous | -1036.24 [-2102.38, 29.90] | 0.057 |  |  |
| Average acceleration * Sex * APOE4 homozygous | -2048.95 [-5199.90, 1102.01] | 0.202 |  |  |
| Bipolar |  |  | -12391.30 [-41058.14, 16275.55] | 0.397 |
| Recurrent depression |  |  | 6251.15 [-270.98, 12773.29] | 0.060 |
| Ethnicity Asian |  |  | -94479.05 [-109238.68, -79719.42] | **<0.001** |
| Ethnicity Black |  |  | -57818.84 [-79778.63, -35859.05] | **<0.001** |
| Ethnicity Mixed |  |  | -43228.28 [-58515.65, -27940.91] | **<0.001** |
| Has smoked |  |  | -1732.34 [-4796.58, 1331.90] | 0.268 |
| Secondary education |  |  | 10779.58 [2327.51, 19231.66] | **0.012** |
| Higher education |  |  | 20533.77 [12756.24, 28311.31] | **<0.001** |
| CVD diagnosis |  |  | -2666.88 [-5915.74, 581.99] | 0.108 |
| Rare alcohol intake |  |  | 7442.58 [696.85, 14188.32] | 0.**031** |
| Frequent alcohol intake |  |  | 4927.46 [-1366.22, 11221.15] | 0.125 |
| Sleep less than 7 hours |  |  | -5249.89 [-8754.81, -1744.97] | **0.003** |
| Sleep more than 9 hours |  |  | -10771.97 [-24018.24, 2474.30] | 0.111 |
| Diabetes diagnosis |  |  | -24032.20 [-30976.01, -17088.39] | **<0.001** |
| BMI obese |  |  | 786.69 [-3777.64, 5351.02] | 0.735 |
| BMI overweight |  |  | 1999.82 [-1350.34, 5349.97] | 0.242 |
| BMI severe obese |  |  | 15894.09 [197.35, 31590.83] | **0.036** |
| BMI underweight |  |  | -17196.38 [-34148.60, -244.16] | **0.047** |
| Age |  |  | -4291.34 [-4502.71, -4079.96] | **<0.001** |

Supplementary Table 35. Results from the sensitivity analysis linear regression where total brain volume was modelled by IPAQ group, ‘best case; data was imputed for missing covariates.

|  | Model one | | Model two | |
| --- | --- | --- | --- | --- |
|  | Estimate | p | Estimate | p |
| IPAQ high | -8448.61 [-15765.96, -1131.26] | **0.024** | 2783.31 [-1330.77, 7007.40] | 0.197 |
| IPAQ moderate | 667.53 [-6471.46, 7806.52] | 0.855 | 3786.62 [-378.33, 7951.58] | 0.075 |
| Sex | 107558.23 [98947.66, 116168.81] | **<0.001** | 123477.98 [120379.15, 126576.81] | **<0.001** |
| APOE4 heterozygous | -2408.74 [-14724.62, 9907.15] | 0.701 | 980.39 [-2544.53, 4505.31] | 0.586 |
| APOE4 homozygous | -9081.46 [-45234.61, 27071.70] | 0.622 | -134.58 [-10156.16, 9887.00] | 0.979 |
| IPAQ high*Sex | 12029.12 [1587.47, 22470.77] | **0.024** |  |  |
| IPAQ moderate*Sex | 2085.62 [-8209.28, 12380.52] | 0.691 |  |  |
| IPAQ high * APOE4 heterozygous | 8344.79 [-6452.23, 23141.81] | 0.269 |  |  |
| IPAQ moderate * APOE4 heterozygous | -2799.37 [-17407.26, 11808.51] | 0.707 |  |  |
| IPAQ high * APOE4 homozygous | 19440.10 [-23992.23, 62872.43] | 0.380 |  |  |
| IPAQ moderate * APOE4 homozygous | 11334.11 [-30841.93, 53510.15] | 0.598 |  |  |
| Sex * APOE4 heterozygous | 10562.04 [-7631.38, 28755.46] | 0.255 |  |  |
| Sex * APOE4 homozygous | 38917.76 [-11356.32, 89191.84] | 0.129 |  |  |
| IPAQ high * Sex * APOE4 heterozygous | -11448.98 [-33227.97, 10330.02] | 0.303 |  |  |
| IPAQ moderate * Sex * APOE4 heterozygous | 5291.41 [-16383.81, 26966.63] | 0.632 |  |  |
| IPAQ high * Sex * APOE4 homozygous | -56854.75 [-117224.06, 3514.57] | 0.065 |  |  |
| IPAQ moderate * Sex * APOE4 homozygous | -44414.40 [-105887.86, 17059.07] | 0.157 |  |  |
| Bipolar |  |  | -12504.36 [-41169.68, 16160.96] | 0.393 |
| Recurrent depression |  |  | 6270.10 [-248.21, 12788.41] | 0.059 |
| Ethnicity Asian |  |  | -94659.53 [-109419.46, -79899.61] | **<0.001** |
| Ethnicity Black |  |  | -57720.58 [-79679.58, -35761.58] | **<0.001** |
| Ethnicity Mixed |  |  | -43271.14 [-58559.51, -27982.78] | **<0.001** |
| Has smoked |  |  | -1703.84 [-4768.10, 1360.41] | 0.276 |
| Secondary education |  |  | 10803.64 [2349.33, 19257.96] | **0.012** |
| Higher education |  |  | 20458.15 [12675.23, 28241.07] | **<0.001** |
| CVD diagnosis |  |  | -2584.68 [-5831.36, 662.00] | 0.119 |
| Rare alcohol intake |  |  | 7507.86 [762.37, 14253.35] | **0.029** |
| Frequent alcohol intake |  |  | 4830.94 [-1461.76, 11123.65] | 0.132 |
| Sleep less than 7 hours |  |  | -5207.73 [-8713.52, -1701.93] | **0.004** |
| Sleep more than 9 hours |  |  | -10406.55 [-23646.95, 2833.85] | 0.123 |
| Diabetes diagnosis |  |  | -23632.21 [-30569.85, -16694.57] | **<0.001** |
| BMI obese |  |  | 1341.51 [-3143.62, 5826.63] | 0.558 |
| BMI overweight |  |  | 2247.25 [-1078.19, 5572.70] | 0.185 |
| BMI severe obese |  |  | 17109.91 [1509.33, 32710.49] | **0.032** |
| BMI underweight |  |  | -17181.99 [-34130.20, -233.77] | **0.047** |
| Age |  |  | -4282.52 [-4489.40, -4075.64] | **<0.001** |

Supplementary Table 36. Results from the sensitivity analysis linear regression where volume of ventricular CSF was modelled by average acceleration, ‘worst case’ data was imputed for missing covariates.

|  | **Model one** | | | **Model two** | |
| --- | --- | --- | --- | --- | --- |
|  | **Estimate** | **p** | **Estimate** | | **p** |
| Average acceleration | -270.85 [-348.49, -192.30] | **<0.001** | -70.07 [-115.47, -24.68] | | **0.002** |
| Sex | 15004.86 [11842.67, 18167.05] | **<0.001** | 7182.38 [6552.12, 7812.65] | | **<0.001** |
| APOE4 heterozygous | -1145.21 [-5693.24, 3402.82] | 0.622 | 366.22 [-351.01, 1083.46] | | 0.317 |
| APOE4 homozygous | -7079.87 [-20923.63, 6763.89] | 0.316 | 409.11 [-11630.20, 2448.41] | | 0.694 |
| Average acceleration*Sex | -194.42 [-304.24, -84.60] | **<0.001** |  | |  |
| Average acceleration*APOE4 heterozygous | 51.81 [-101.21, 204.83] | 0.507 |  | |  |
| Average acceleration* APOE4 homozygous | 205.37 [-258.34, 669.08] | 0.385 |  | |  |
| Sex * APOE4 heterozygous | -1872.95 [-8377.02, 4631.13] | 0.572 |  | |  |
| Sex * APOE4 homozygous | 7849.62 [-11616.82, 27316.07] | 0.429 |  | |  |
| Average acceleration * Sex * APOE4 heterozygous | 30.41 [-193.24, 254.06] | 0.790 |  | |  |
| Average acceleration * Sex * APOE4 homozygous | -181.63 [-842.63, 379.38] | 0.590 |  | |  |
| Bipolar |  |  | -667.18 [-6531.64, 5197.27] | | 0.824 |
| Recurrent depression |  |  | -25.52 [-770.70, 719.66] | | 0.946 |
| Ethnicity Asian |  |  | -1806.46 [-4809.83, 1196.91] | | 0.238 |
| Ethnicity Black |  |  | -4500.49 [-8128.39, -872.59] | | **0.015** |
| Ethnicity Mixed |  |  | 287.28 [-2824.07, 3398.63] | | 0.856 |
| Has smoked |  |  | 467.14 [-1156.51, 1090.79] | | 0.142 |
| Secondary education |  |  | 612.32 [-1042.01, 2266.64] | | 0.468 |
| Higher education |  |  | 1949.18 [437.96, 3460.39] | | **0.011** |
| CVD diagnosis |  |  | 1158.18 [497.17, 1819.18] | | **0.001** |
| Rare alcohol intake |  |  | -1414.86 [-2787.71, -42.01] | | **0.043** |
| Frequent alcohol intake |  |  | -77.92 [-1358.42, 1202.58] | | 0.905 |
| Sleep less than 7 hours |  |  | 13.04 [-698.82, 724.89] | | 0.971 |
| Sleep more than 9 hours |  |  | 1766.09 [-930.24, 4462.42] | | 0.199 |
| Diabetes diagnosis |  |  | 3478.27 [2065.15, 4891.39] | | **<0.001** |
| BMI obese |  |  | -112.57 [-1041.38, 816.24] | | 0.812 |
| BMI overweight |  |  | -566.33 [-1248.09, 115.44] | | 0.103 |
| BMI severe obese |  |  | 2194.55 [-999.67, 5388.77] | | 0.178 |
| BMI underweight |  |  | -610.64 [-4060.62, 2839.33] | | 0.729 |
| Age |  |  | 1133.11 [1090.21, 1176.01] | | **<0.001** |

Supplementary Table 37. Results from the sensitivity analysis linear regression where volume of ventricular CSF was modelled by IPAQ group, ‘worst case’ data was imputed for missing covariates.

|  | Model one | | Model two | |
| --- | --- | --- | --- | --- |
|  | Estimate | p | Estimate | p |
| IPAQ high | 1604.37 [61.65, 3147.10] | **0.042** | 853.51 [-6.68, 1713.70] | 0.052 |
| IPAQ moderate | 1117.52 [-387.60, 2622.64] | 0.146 | 800.01 [-47.93, 1647.95] | 0.064 |
| Sex | 9317.67 [7502.30, 11133.05] | **<0.001** | 7221.26 [6590.72, 7851.80] | **<0.001** |
| APOE4 heterozygous | -661.14 [-3257.71, 1935.43] | 0.618 | 339.18 [-378.24, 1056.60] | 0.354 |
| APOE4 homozygous | -2236.77 [-9858.96, 5385.43] | 0.565 | 378.33 [-1661.41, 2418.07] | 0.716 |
| IPAQ high*Sex | 366.14 [-1835.28, 2567.56] | 0.744 |  |  |
| IPAQ moderate*Sex | 1230.37 [-940.11, 3400.85] | 0.267 |  |  |
| IPAQ high * APOE4 heterozygous | 1658.11 [-1461.55, 4777.78] | 0.298 |  |  |
| IPAQ moderate * APOE4 heterozygous | 442.41 [-2637.38, 3522.20] | 0.778 |  |  |
| IPAQ high * APOE4 homozygous | 1573.32 [-7583.56, 10730.19] | 0.736 |  |  |
| IPAQ moderate * APOE4 homozygous | 774.06 [-8117.95, 9666.07] | 0.865 |  |  |
| Sex * APOE4 heterozygous | -54.50 [-3890.23, 3781.24] | 0.978 |  |  |
| Sex * APOE4 homozygous | 5127.37 [-5471.95, 15726.70] | 0.343 |  |  |
| IPAQ high * Sex * APOE4 heterozygous | -1242.20 [-5833.89, 3349.48] | 0.596 |  |  |
| IPAQ moderate * Sex * APOE4 heterozygous | -1300.42 [-5870.23, 3269.38] | 0.577 |  |  |
| IPAQ high * Sex * APOE4 homozygous | -1106.09 [-13833.80, 11621.62] | 0.865 |  |  |
| IPAQ moderate * Sex * APOE4 homozygous | -6937.26 [-19897.86, 6023.24] | 0.294 |  |  |
| Bipolar |  |  | -691.93 [-6557.68, 5173.81] | 0.817 |
| Recurrent depression |  |  | 20.15 [-725.21, 765.51] | 0.958 |
| Ethnicity Asian |  |  | -1849.29 [-4853.56, 1154.98] | 0.228 |
| Ethnicity Black |  |  | -4439.53 [-8068.20, -810.86] | **0.016** |
| Ethnicity Mixed |  |  | 266.10 [-2846.32, 3378.53] | 0.867 |
| Has smoked |  |  | 469.90 [-153.92, 1093.71] | 0.140 |
| Secondary education |  |  | 675.01 [-980.27, 2330.29] | 0.424 |
| Higher education |  |  | 1987.28 [474.52, 3500.03] | **0.010** |
| CVD diagnosis |  |  | 1209.40 [548.67, 1870.13] | **<0.001** |
| Rare alcohol intake |  |  | -1378.15 [-2751.33, -4.97] | **0.049** |
| Frequent alcohol intake |  |  | -122.47 [-1403.12, 1158.17] | 0.851 |
| Sleep less than 7 hours |  |  | 8.94 [-703.29, 721.16] | 0.980 |
| Sleep more than 9 hours |  |  | 1947.26 [-748.61, 4643.13] | 0.157 |
| Diabetes diagnosis |  |  | 3676.76 [2263.48, 5089.04] | **<0.001** |
| BMI obese |  |  | 238.51 [-674.28, 1151.30] | 0.609 |
| BMI overweight |  |  | -407.92 [-1084.83, 268.99] | 0.238 |
| BMI severe obese |  |  | 2909.12 [-266.61, 6084.85] | 0.073 |
| BMI underweight |  |  | -698.79 [-4148.93, 2751.36] | 0.691 |
| Age |  |  | 1144.68 [1102.66, 1186.69] | **<0.001** |

Supplementary Table 38. Results from the sensitivity analysis linear regression where total brain volume was modelled by average acceleration, ‘worst case’ data was imputed for missing covariates.

|  | Model one | | | Model two | |
| --- | --- | --- | --- | --- | --- |
|  | Estimate | p | Estimate | | p |
| Average acceleration | 709.68 [339.56, 1079.80] | .**<0.001** | -99.07 [-322.20, 124.05] | | 0.384 |
| Sex | 101891.79 [86817.93, 116965.65] | **<0.001** | 123156.69 [120058.76, 126254.62] | | **<0.001** |
| APOE4 heterozygous | -24052.32 [-45732.35, -2372.29] | **0.030** | 917.96 [-2607.45, 4443.37] | | 0.610 |
| APOE4 homozygous | -1277.29 [-67269.22, 64714.64] | 0.970 | -427.18 [-10450.94, 9596.58] | | 0.933 |
| Average acceleration*Sex | 433.19 [-90.31, 956.69] | 0.105 |  | |  |
| Average acceleration*APOE4 heterozygous | 798.79 [69.37, 1528.21] | **0.032** |  | |  |
| Average acceleration* APOE4 homozygous | 145.84 [-2064.62, 2356.31] | 0.897 |  | |  |
| Sex * APOE4 heterozygous | 38281.37 [7277.05, 69285.69] | **0.016** |  | |  |
| Sex * APOE4 homozygous | 55608.82 [-37185.91,148403.56] | 0.240 |  | |  |
| Average acceleration * Sex * APOE4 heterozygous | -1036.24 [-2102.38, 29.90] | 0.057 |  | |  |
| Average acceleration * Sex * APOE4 homozygous | -2048.95 [-5199.90, 1102.01] | 0.202 |  | |  |
| Bipolar |  |  | -12641.43 [-41466.85, 16183.99] | | 0.390 |
| Recurrent depression |  |  | -1066.73 [-4729.51, 2596.04] | | 0.568 |
| Ethnicity Asian |  |  | -93902.54 [-108664.95, -79140.14] | | **<0.001** |
| Ethnicity Black |  |  | -39168.00 [-57000.13, -21335.86] | | **<0.001** |
| Ethnicity Mixed |  |  | -42991.17 [-58284.31, -27698.02] | | **<0.001** |
| Has smoked |  |  | -1575.13 [-4640.53, 1490.27] | | 0.314 |
| Secondary education |  |  | 9754.17 [1622.71, 17885.63] | | **0.019** |
| Higher education |  |  | 19641.61 [12213.57, 27069.66] | | <**0.001** |
| CVD diagnosis |  |  | -2663.09 [-5912.13, 585.96] | | 0.108 |
| Rare alcohol intake |  |  | 7497.26 [749.32, 14245.21] | | **0.029** |
| Frequent alcohol intake |  |  | 4966.30 [-1327.73, 11260.33] | | 0.122 |
| Sleep less than 7 hours |  |  | -5721.33 [-9220.29, -222.37] | | **0.001** |
| Sleep more than 9 hours |  |  | -10763.30 [-24016.52, 2489.92] | | 0.111 |
| Diabetes diagnosis |  |  | -23979.29 [-30925.18, -17033.40] | | **<0.001** |
| BMI obese |  |  | 967.14 [-3598.21, 5532.50 | | 0.678 |
| BMI overweight |  |  | 2034.24 [-1316.84, 5385.31] | | 0.223 |
| BMI severe obese |  |  | 15400.28 [-300.19, 31100.75] | | 0.055 |
| BMI underweight |  |  | -16947.57 [-33905.15, 10.02] | | 0.050 |
| Age |  |  | -4291.55 [-4502.43, -4080.67] | | **<0.001** |

Supplementary Table 39. Results from the sensitivity analysis linear regression where total brain volume was modelled by IPAQ group, ‘worst case’ data was imputed for missing covariates.

|  | Model one | | Model two | | |  |
| --- | --- | --- | --- | --- | --- | --- |
|  | Estimate | p | | Estimate | p | |
| IPAQ high | -8448.61 [-15765.96, -1131.26] | **0.024** | | 2803.50 [-1330.77, 7007.40] | 0.194 | |
| IPAQ moderate | 667.53 [-6471.46, 7806.52] | 0.855 | | 3824.70 [-378.33, 7951.58] | 0.072 | |
| Sex | 107558.23 [98947.66, 116168.81] | **<0.001** | | 123273.83 [120379.15, 126576.81] | **<0.001** | |
| APOE4 heterozygous | -2408.74 [-14724.62, 9907.15] | 0.701 | | 878.70 [-2544.53, 4505.31] | 0.625 | |
| APOE4 homozygous | -9081.46 [-45234.61, 27071.70] | 0.622 | | -426.95 [-10156.16, 9887.00] | 0.933 | |
| IPAQ high*Sex | 12029.12 [1587.47, 22470.77] | **0.024** | |  |  | |
| IPAQ moderate*Sex | 2085.62 [-8209.28, 12380.52] | 0.691 | |  |  | |
| IPAQ high * APOE4 heterozygous | 8344.79 [-6452.23, 23141.81] | 0.269 | |  |  | |
| IPAQ moderate * APOE4 heterozygous | -2799.37 [-17407.26, 11808.51] | 0.707 | |  |  | |
| IPAQ high * APOE4 homozygous | 19440.10 [-23992.23, 62872.43] | 0.380 | |  |  | |
| IPAQ moderate * APOE4 homozygous | 11334.11 [-30841.93, 53510.15] | 0.598 | |  |  | |
| Sex * APOE4 heterozygous | 10562.04 [-7631.38, 28755.46] | 0.255 | |  |  | |
| Sex * APOE4 homozygous | 38917.76 [-11356.32, 89191.84] | 0.129 | |  |  | |
| IPAQ high * Sex * APOE4 heterozygous | -11448.98 [-33227.97, 10330.02] | 0.303 | |  |  | |
| IPAQ moderate * Sex * APOE4 heterozygous | 5291.41 [-16383.81, 26966.63] | 0.632 | |  |  | |
| IPAQ high * Sex * APOE4 homozygous | -56854.75 [-117224.06, 3514.57] | 0.065 | |  |  | |
| IPAQ moderate * Sex * APOE4 homozygous | -44414.40 [-105887.86, 17059.07] | 0.157 | |  |  | |
| Bipolar |  |  | | -12686.67 [-41169.68, 16160.96] | 0.388 | |
| Recurrent depression |  |  | | -967.35 [-248.21, 12788.41] | 0.605 | |
| Ethnicity Asian |  |  | | -94078.55 [-109419.46, -79899.61] | **<0.001** | |
| Ethnicity Black |  |  | | -39012.36 [-79679.58, -35761.58] | **<0.001** | |
| Ethnicity Mixed |  |  | | -43034.11 [-58559.51, -27982.78] | **<0.001** | |
| Has smoked |  |  | | -1549.98 [-4768.10, 1360.41] | 0.322 | |
| Secondary education |  |  | | 9783.24 [2349.33, 19257.96] | **0.018** | |
| Higher education |  |  | | 19566.06 [12675.23, 28241.07] | **<0.001** | |
| CVD diagnosis |  |  | | -2572.97 [-5831.36, 662.00] | 0.120 | |
| Rare alcohol intake |  |  | | 7566.67 [762.37, 14253.35] | **0.028** | |
| Frequent alcohol intake |  |  | | 4860.73 [-1461.76, 11123.65] | 0.130 | |
| Sleep less than 7 hours |  |  | | -5682.73 [-8713.52, -1701.93] | **0.001** | |
| Sleep more than 9 hours |  |  | | -10372.52 [-23646.95, 2833.85] | 0.125 | |
| Diabetes diagnosis |  |  | | -23549.51 [-30569.85, -16694.57] | **<0.001** | |
| BMI obese |  |  | | 1582.51 [-3143.62, 5826.63] | 0.489 | |
| BMI overweight |  |  | | 2309.22 [-1078.19, 5572.70] | 0.174 | |
| BMI severe obese |  |  | | 16740.65 [1509.33, 32710.49] | **0.036** | |
| BMI underweight |  |  | | -16956.36 [-34130.20, -233.77] | 0.050 | |
| Age |  |  | | -4279.84 [-4489.40, -4075.64] | **<0.001** | |

Supplementary Table 40. Results from the sensitivity analysis linear regression where duration to complete the TMT-B was modelled by average acceleration, ‘worst case’ data was imputed for missing covariates.

|  | Model one | | Model two | |
| --- | --- | --- | --- | --- |
|  | Estimate | p | Estimate | p |
| Average acceleration | -0.05 [-0.06, -0.03] | **<0.001** | 0.01 [0.00, 0.02] | **0.039** |
| Sex | 0.04 [-0.60, 0.69] | 0.892 | -0.03 [-0.17, 0.09] | 0.603 |
| APOE4 heterozygous | -0.54 [-1.48, 0.40] | 0.257 | 0.09 [-0.07, 0.23] | 0.212 |
| APOE4 homozygous | -0.15 [-2.85, 2.55] | 0.913 | 0.03 [-0.38, 0.46] | 0.890 |
| Average acceleration*Sex | 0.01 [-0.02, 0.03] | 0.577 |  |  |
| Average acceleration*APOE4 heterozygous | 0.02 [-0.01, 0.05] | 0.178 |  |  |
| Average acceleration* APOE4 homozygous | 0.00 [-0.09, 0.09] | 0.957 |  |  |
| Sex * APOE4 heterozygous | 0.07 [-1.27, 1.41] | 0.919 |  |  |
| Sex * APOE4 homozygous | -0.69 [-4.46, 3.09] | 0.722 |  |  |
| Average acceleration * Sex * APOE4 heterozygous | -0.01 [-0.05, 0.04] | 0.771 |  |  |
| Average acceleration * Sex * APOE4 homozygous | 0.03 [-0.10, 0.16] | 0.672 |  |  |
| Bipolar |  |  | -0.38 [-1.71, 0.69] | 0.527 |
| Recurrent depression |  |  | 0.20 [-0.11, 0.44] | **0.010** |
| Ethnicity Asian |  |  | 1.51 [0.90, 2.16] | **<0.001** |
| Ethnicity Black |  |  | 1.49 [0.78, 2.83] | **0.001** |
| Ethnicity Mixed |  |  | 0.72 [0.22, 1.51] | **0.024** |
| Has smoked |  |  | 0.12 [-0.01, 0.24] | 0.054 |
| Secondary education |  |  | -1.26 [-1.69, -0.87] | **<0.001** |
| Higher education |  |  | -2.01 [-2.43, -1.66] | **<0.001** |
| CVD diagnosis |  |  | 0.22 [0.12, 0.40] | **0.001** |
| Rare alcohol intake |  |  | -0.22 [-0.51, 0.07] | 0.125 |
| Frequent alcohol intake |  |  | -0.36 [-0.64, -0.11] | **0.007** |
| Sleep less than 7 hours |  |  | 0.14 [-0.01, 0.28] | 0.061 |
| Sleep more than 9 hours |  |  | 0.86 [0.22, 1.34] | **0.002** |
| Diabetes diagnosis |  |  | 0.44 [0.13, 0.72] | **0.003** |
| BMI obese |  |  | 0.33 [0.15, 0.53] | **<0.001** |
| BMI overweight |  |  | 0.04 [-0.10, 0.18] | 0.551 |
| BMI severe obese |  |  | 0.01 [-0.59, 0.65] | 0.985 |
| BMI underweight |  |  | 0.71 [0.08, 1.42] | **0.035** |
| Age |  |  | 0.18 [0.17, 0.19] | **<0.001** |

Supplementary Table 41. Results from the sensitivity analysis linear regression where duration to complete the TMT-B was modelled by IPAQ group, ‘worst case’ data was imputed for missing covariates.

|  | Model one | | Model two | |
| --- | --- | --- | --- | --- |
|  | Estimate | p | Estimate | p |
| IPAQ high | 0.41 [0.10, 0.72] | 0.**010** | 0.51 [0.34, 0.69] | **<0.001** |
| IPAQ moderate | 0.07 [-0.23, 0.37] | 0.669 | 0.29 [0.12, 0.46] | **<0.001** |
| Sex | -0.01 [-0.37, 0.35] | 0.957 | -0.04 [-0.17, 0.09] | 0.561 |
| APOE4 heterozygous | -0.01 [-0.53, 0.51] | 0.967 | 0.09 [-0.06, 0.23] | 0.237 |
| APOE4 homozygous | 0.63 [-0.85, 2.11] | 0.406 | 0.04 [-0.38, 0.46] | 0.852 |
| IPAQ high*Sex | 0.29 [-0.14, 0.73] | 0.186 |  |  |
| IPAQ moderate*Sex | 0.36 [-0.06, 0.79] | 0.095 |  |  |
| IPAQ high * APOE4 heterozygous | 0.10 [-0.53, 0.73] | 0.750 |  |  |
| IPAQ moderate * APOE4 heterozygous | 0.04 [-0.58, 0.66] | 0.894 |  |  |
| IPAQ high * APOE4 homozygous | -1.17 [-2.99, 0.65] | 0.210 |  |  |
| IPAQ moderate * APOE4 homozygous | -1.02 [-2.79, 0.76] | 0.261 |  |  |
| Sex * APOE4 heterozygous | -0.08 [-0.82, 0.67] | 0.841 |  |  |
| Sex * APOE4 homozygous | -1.55 [-3.53, 0.42] | 0.122 |  |  |
| IPAQ high * Sex * APOE4 heterozygous | -0.19 [-1.09, 0.72] | 0.681 |  |  |
| IPAQ moderate * Sex * APOE4 heterozygous | 0.07 [-0.82, 0.97] | 0.871 |  |  |
| IPAQ high * Sex * APOE4 homozygous | 2.95 [0.46, 5.44] | **0.020** |  |  |
| IPAQ moderate * Sex * APOE4 homozygous | 1.48 [-0.97, 3.93] | 0.235 |  |  |
| Bipolar |  |  | -0.39 [-1.57, 0.80] | 0.524 |
| Recurrent depression |  |  | 0.21 [0.06, 0.37] | **0.006** |
| Ethnicity Asian |  |  | 1.49 [0.87, 2.12] | **<0.001** |
| Ethnicity Black |  |  | 1.48 [0.69, 2.28] | **<0.001** |
| Ethnicity Mixed |  |  | 0.68 [0.05, 1.31] | **0.033** |
| Has smoked |  |  | 0.13 [0.00, 0.25] | **0.048** |
| Secondary education |  |  | -1.26 [-1.64, -0.87] | **<0.001** |
| Higher education |  |  | -2.01 [-2.37, -1.66] | **<0.001** |
| CVD diagnosis |  |  | 0.22 [0.08, 0.35] | **0.001** |
| Rare alcohol intake |  |  | -0.22 [-0.51, 0.06] | 0.118 |
| Frequent alcohol intake |  |  | -0.36 [-0.62, -0.10] | **0.007** |
| Sleep less than 7 hours |  |  | 0.14 [0.00, 0.29] | 0.056 |
| Sleep more than 9 hours |  |  | 0.86 [0.31, 1.42] | **0.002** |
| Diabetes diagnosis |  |  | 0.45 [0.16, 0.74] | **0.002** |
| BMI obese |  |  | 0.33 [0.15, 0.52] | **<0.001** |
| BMI overweight |  |  | 0.04 [-0.10, 0.17] | 0.606 |
| BMI severe obese |  |  | 0.03 [-0.59, 0.64] | **0.927** |
| BMI underweight |  |  | 0.74 [0.08, 1.39] | **0.028** |
| Age |  |  | 0.18 [0.17, 0.18] | **<0.001** |

Supplementary Table 42. Results from the sensitivity analysis linear regression where mean time to correctly identify matches was modelled by average acceleration, ‘worst case’ data was imputed for missing covariates.

|  | Model one | | Model two | |
| --- | --- | --- | --- | --- |
|  | Estimate | p | Estimate | p |
| Average acceleration | -1.84 [-2.24, -1.43] | **<0.001** | -0.43 [-0.67, -0.18] | **<0.001** |
| Sex | -36.06 [-52.46, -19.66] | **<0.001** | -23.19 [-26.59, -19.79] | **<0.001** |
| APOE4 heterozygous | -30.02 [-53.91, -6.12] | **0.014** | -2.16 [-6.03, 1.72] | 0.276 |
| APOE4 homozygous | -61.31 [-130.83, 6.21] | 0.075 | 5.73 [-5.44, 16.90] | 0.315 |
| Average acceleration*Sex | 0.62 [0.06, 1.18] | **0.031** |  |  |
| Average acceleration*APOE4 heterozygous | 0.97 [0.18, 1.77] | **0.016** |  |  |
| Average acceleration* APOE4 homozygous | 1.93 [-0.34, 4.20] | 0.096 |  |  |
| Sex * APOE4 heterozygous | 2.13 [-31.97, 36.22] | 0.903 |  |  |
| Sex * APOE4 homozygous | 67.69 [-28.24, 163.61] | 0.167 |  |  |
| Average acceleration * Sex * APOE4 heterozygous | -0.19 [-1.35, 0.97] | 0.745 |  |  |
| Average acceleration * Sex * APOE4 homozygous | -1.76 [-5.03, 1.50] | 0.290 |  |  |
| Bipolar |  |  | -6.80 [-38.41, 24.80] | 0.673 |
| Recurrent depression |  |  | 4.58 [0.53, 8.63] | **0.027** |
| Ethnicity Asian |  |  | 25.56 [8.87, 42.25] | **0.003** |
| Ethnicity Black |  |  | 39.98 [18.74, 61.22] | **<0.001** |
| Ethnicity Mixed |  |  | 13.61 [-3.12, 30.34] | 0.111 |
| Has smoked |  |  | 1.46 [-1.91, 4.83] | 0.394 |
| Secondary education |  |  | -13.12 [-23.33, -2.91] | **0.012** |
| Higher education |  |  | -20.82 [-30.31, -11.33] | **<0.001** |
| CVD diagnosis |  |  | -0.32 [-3.90, 3.26] | 0.861 |
| Rare alcohol intake |  |  | 4.18 [-3.33, 11.69] | 0.276 |
| Frequent alcohol intake |  |  | 0.51 [-6.48, 7.50] | 0.886 |
| Sleep less than 7 hours |  |  | -1.10 [-4.96, 2.77] | 0.578 |
| Sleep more than 9 hours |  |  | 9.85 [-4.88, 24.58] | 0.190 |
| Diabetes diagnosis |  |  | 3.17 [-4.56, 10.91] | 0.422 |
| BMI obese |  |  | 3.52 [-1.53, 8.57] | 0.172 |
| BMI overweight |  |  | -0.04 [-3.73, 3.65] | 0.985 |
| BMI severe obese |  |  | -8.82 [-25.31, 7.67] | 0.294 |
| BMI underweight |  |  | 17.26 [-0.28, 34.80] | 0.054 |
| Age |  |  | 3.59 [3.36, 3.83] | <0.**001** |

Supplementary Table 43. Results from the sensitivity analysis linear regression where mean time to correctly identify matches was modelled by IPAQ group, ‘worst case’ data was imputed for missing covariates.

|  | Model one | | | Model two | |
| --- | --- | --- | --- | --- | --- |
|  | Estimate | p | Estimate | | p |
| IPAQ high | -1.18 [-9.11, 6.75] | 0.771 | -1.33 [-5.94, 3.28] | | 0.572 |
| IPAQ moderate | -4.21 [-11.88, 3.45] | 0.281 | -0.64 [-5.15, 3.87] | | 0.782 |
| Sex | -22.17 [-31.25, -13.08] | **<0.001** | -23.02 [-26.43, -19.62] | | **<0.001** |
| APOE4 heterozygous | -5.45 [-18.78, 7.89] | 0.423 | -2.24 [-6.12, 1.64] | | 0.258 |
| APOE4 homozygous | -8.55 [-46.47, 29.36] | 0.658 | 5.76 [-5.42, 16.94] | | 0.312 |
| IPAQ high*Sex | 4.18 [-6.97, 15.33] | 0.463 |  | |  |
| IPAQ moderate*Sex | 7.70 [-3.20, 18.61] | 0.166 |  | |  |
| IPAQ high * APOE4 heterozygous | 2.59 [-13.54, 18.71] | 0.753 |  | |  |
| IPAQ moderate * APOE4 heterozygous | 4.21 [-11.59, 20.00] | 0.601 |  | |  |
| IPAQ high * APOE4 homozygous | -10.69 [-57.30, 35.92] | 0.653 |  | |  |
| IPAQ moderate * APOE4 homozygous | 13.08 [-32.27, 58.43] | 0.572 |  | |  |
| Sex * APOE4 heterozygous | 0.26 [-18.82, 19.33] | 0.979 |  | |  |
| Sex * APOE4 homozygous | 33.32 [-17.05, 83.68] | 0.195 |  | |  |
| IPAQ high * Sex * APOE4 heterozygous | -5.66 [-28.78, 17.47] | 0.632 |  | |  |
| IPAQ moderate * Sex * APOE4 heterozygous | -3.48 [-26.33, 19.38] | 0.765 |  | |  |
| IPAQ high * Sex * APOE4 homozygous | 2.31 [-61.27, 65.88] | 0.943 |  | |  |
| IPAQ moderate * Sex * APOE4 homozygous | -43.11 [-105.61, 19.39] | 0.176 |  | |  |
| Bipolar |  |  | -6.99 [-38.61, 24.63] | | 0.665 |
| Recurrent depression |  |  | 4.63 [0.57, 8.68] | | **0.025** |
| Ethnicity Asian |  |  | 25.70 [9.00, 42.41] | | **0.003** |
| Ethnicity Black |  |  | 40.36 [19.11, 61.61] | | **<0.001** |
| Ethnicity Mixed |  |  | 14.12 [-2.62, 30.86] | | 0.098 |
| Has smoked |  |  | 1.40 [-1.97, 4.77] | | 0.416 |
| Secondary education |  |  | -12.83 [-23.05, -2.61] | | **0.014** |
| Higher education |  |  | -20.45 [-29.94, -10.95] | | **<0.001** |
| CVD diagnosis |  |  | -0.09 [-3.66, 3.49] | | 0.962 |
| Rare alcohol intake |  |  | 4.55 [-2.96, 12.07] | | 0.235 |
| Frequent alcohol intake |  |  | 0.45 [-6.54, 7.45] | | 0.899 |
| Sleep less than 7 hours |  |  | -1.18 [-5.05, 2.69] | | 0.549 |
| Sleep more than 9 hours |  |  | 10.37 [-4.37, 25.11] | | 0.168 |
| Diabetes diagnosis |  |  | 3.94 [-3.79, 11.67] | | 0.318 |
| BMI obese |  |  | 5.08 [0.10, 10.05] | | **0.045** |
| BMI overweight |  |  | 0.70 [-2.97, 4.37] | | 0.708 |
| BMI severe obese |  |  | -5.89 [-22.32, 10.53] | | 0.482 |
| BMI underweight |  |  | 16.49 [-1.06, 34.03] | | 0.066 |
| Age |  |  | 3.68 [3.45, 3.91] | | **<0.001** |

Supplementary Table 44. Results from the sensitivity analysis linear regression where % correct on the pairs matching task was modelled by average acceleration, ‘worst case’ data was imputed for missing covariates.

|  | Model one | | Model two | |
| --- | --- | --- | --- | --- |
|  | Estimate | p | Estimate | p |
| Average acceleration | 0.01 [-0.04, 0.06] | 0.634 | -0.06 [-0.09, -0.03] | **<0.001** |
| Sex | -0.52 [-2.63, 1.58] | 0.625 | 0.51 [0.06, 0.96] | **0.026** |
| APOE4 heterozygous | 2.00 [-1.07, 5.07] | 0.202 | -0.42 [-0.93, 0.09] | 0.109 |
| APOE4 homozygous | -5.12 [-13.92, 3.67] | 0.254 | -0.57 [-2.04, 0.91] | 0.451 |
| Average acceleration*Sex | 0.02 [-0.06, 0.09] | 0.667 |  |  |
| Average acceleration*APOE4 heterozygous | -0.09 [-0.19, 0.01] | 0.076 |  |  |
| Average acceleration* APOE4 homozygous | 0.19 [-0.10, 0.48] | 0.198 |  |  |
| Sex * APOE4 heterozygous | -1.16 [-5.54, 3.22] | 0.604 |  |  |
| Sex * APOE4 homozygous | 6.64 [-5.67, 18.95] | 0.291 |  |  |
| Average acceleration * Sex * APOE4 heterozygous | 0.07 [-0.08, 0.22] | 0.373 |  |  |
| Average acceleration * Sex * APOE4 homozygous | -0.28 [-0.70, 0.13] | 0.184 |  |  |
| Bipolar |  |  | -0.61 [-4.79, 3.57] | 0.775 |
| Recurrent depression |  |  | -0.45 [-0.09, 0.98] | 0.100 |
| Ethnicity Asian |  |  | -3.28 [-5.48, -1.07] | **0.004** |
| Ethnicity Black |  |  | -5.40 [-8.21, -2.60] | **<0.001** |
| Ethnicity Mixed |  |  | -2.76 [-4.97, -0.55] | **0.014** |
| Has smoked |  |  | -0.35 [-0.80, 0.10] | 0.123 |
| Secondary education |  |  | 1.53 [0.18, 2.88] | **0.026** |
| Higher education |  |  | 2.27 [1.02, 3.53] | **<0.001** |
| CVD diagnosis |  |  | -0.16 [-0.63, 0.32] | 0.517 |
| Rare alcohol intake |  |  | 0.93 [-0.07, 1.92] | 0.067 |
| Frequent alcohol intake |  |  | 0.59 [-0.33, 1.52] | 0.209 |
| Sleep less than 7 hours |  |  | -0.11 [-0.63, 0.40] | 0.660 |
| Sleep more than 9 hours |  |  | -1.39 [-3.34, 0.55] | 0.161 |
| Diabetes diagnosis |  |  | -0.95 [-1.97, 0.07] | 0.068 |
| BMI obese |  |  | 0.12 [-0.55, 0.79] | 0.724 |
| BMI overweight |  |  | 0.08 [-0.41, 0.57] | 0.744 |
| BMI severe obese |  |  | -0.48 [-2.66, 1.70] | 0.664 |
| BMI underweight |  |  | -2.48 [-4.80, -0.16] | **0.035** |
| Age |  |  | -0.28 [-0.31, -0.25] | **<0.001** |

Supplementary Table 45. Results from the sensitivity analysis linear regression where % correct on the pairs matching task was modelled by IPAQ group, ‘worst case’ data was imputed for missing covariates.

|  | Model one | | | Model two | | |
| --- | --- | --- | --- | --- | --- | --- |
|  | Estimate | p | Estimate | | p |  |
| IPAQ high | -1.19 [-2.20, -0.17] | **0.022** | -0.36 [-0.97, 0.25] | | 0.251 |  |
| IPAQ moderate | -0.34 [-1.31, 0.64] | 0.602 | -0.04 [-0.63, 0.56] | | 0.903 |  |
| Sex | -0.12 [-1.28, 1.04] | 0.836 | 0.54 [0.09, 0.99] | | **0.019** |  |
| APOE4 heterozygous | -2.28 [-3.98, -0.58] | **0.009** | -0.43 [-0.94, 0.09] | | 0.104 |  |
| APOE4 homozygous | -2.03 [-6.86, 2.81] | 0.412 | -0.56 [-2.04, 0.92] | | 0.456 |  |
| IPAQ high*Sex | 0.37 [-1.05, 1.80] | 0.606 |  | |  |  |
| IPAQ moderate*Sex | -0.25 [-1.64, 1.14] | 0.722 |  | |  |  |
| IPAQ high * APOE4 heterozygous | 2.07 [0.01, 4.13] | **0.048** |  | |  |  |
| IPAQ moderate * APOE4 heterozygous | 1.80 [-0.22, 3.81] | 0.080 |  | |  |  |
| IPAQ high * APOE4 homozygous | 3.40 [-2.55, 9.34] | 0.263 |  | |  |  |
| IPAQ moderate * APOE4 homozygous | 2.88 [-2.91, 8.66] | 0.329 |  | |  |  |
| Sex * APOE4 heterozygous | 1.34 [-1.10, 3.77] | 0.281 |  | |  |  |
| Sex * APOE4 homozygous | 1.77 [-4.65, 8.20] | 0.589 |  | |  |  |
| IPAQ high * Sex * APOE4 heterozygous | -0.57 [-3.52, 2.38] | 0.705 |  | |  |  |
| IPAQ moderate * Sex * APOE4 heterozygous | -0.59 [-3.51, 2.32] | 0.689 |  | |  |  |
| IPAQ high * Sex * APOE4 homozygous | -4.64 [-12.57, 3.65] | 0.281 |  | |  |  |
| IPAQ moderate * Sex * APOE4 homozygous | -4.12 [-12.09, 3.86] | 0.312 |  | |  |  |
| Bipolar |  |  | -0.64 [-4.82, 3.54] | | 0.764 |  |
| Recurrent depression |  |  | 0.45 [0.09, 0.99] | | 0.099 |  |
| Ethnicity Asian |  |  | -3.26 [-5.64, -1.05] | | **0.004** |  |
| Ethnicity Black |  |  | -5.35 [-8.15, -2.54] | | **<0.001** |  |
| Ethnicity Mixed |  |  | -2.66 [-4.88, -0.45] | | **0.018** |  |
| Has smoked |  |  | -0.36 [-0.80, 0.09] | | 0.116 |  |
| Secondary education |  |  | 1.56 [0.21, 2.91] | | **0.023** |  |
| Higher education |  |  | 2.31 [1.06, 3.56] | | **<0.001** |  |
| CVD diagnosis |  |  | -0.13 [-0.60, 0.35] | | 0.603 |  |
| Rare alcohol intake |  |  | 0.97 [-0.02, 1.97] | | 0.054 |  |
| Frequent alcohol intake |  |  | 0.58 [-0.35, 1.50] | | 0.219 |  |
| Sleep less than 7 hours |  |  | -0.12 [-0.63, 0.39] | | 0.638 |  |
| Sleep more than 9 hours |  |  | -1.32 [-3.26, 0.63] | | 0.185 |  |
| Diabetes diagnosis |  |  | -0.86 [-1.88, 0.16] | | 0.100 |  |
| BMI obese |  |  | 0.32 [-0.34, 0.98] | | 0.339 |  |
| BMI overweight |  |  | 0.18 [-0.30, 0.66] | | 0.466 |  |
| BMI severe obese |  |  | -0.11 [-2.28, 2.06] | | 0.921 |  |
| BMI underweight |  |  | -2.58 [-4.90, -0.26] | | **0.029** |  |
| Age |  |  | -0.27 [-0.30, -0.24] | | **<0.001** |  |

Supplementary Table 46. Results from the sensitivity analysis linear regression where duration to complete the TMT-B was modelled by average acceleration, ‘best case’ data was imputed for missing covariates.

|  | Model one | | Model two | |
| --- | --- | --- | --- | --- |
|  | Estimate | p | Estimate | p |
| Average acceleration | -0.05 [-0.06, -0.03] | **<0.001** | 0.01 [0.00, 0.02] | **0.042** |
| Sex | 0.04 [-0.60, 0.69] | 0.892 | -0.04 [-0.17, 0.08] | 0.510 |
| APOE4 heterozygous | -0.54 [-1.48, 0.40] | 0.257 | 0.09 [-0.06, 0.24] | 0.227 |
| APOE4 homozygous | -0.15 [-2.85, 2.55] | 0.913 | 0.02 [-0.40, 0.44] | 0.929 |
| Average acceleration*Sex | 0.01 [-0.02, 0.03] | 0.577 |  |  |
| Average acceleration*APOE4 heterozygous | 0.02 [-0.01, 0.05] | 0.178 |  |  |
| Average acceleration* APOE4 homozygous | 0.00 [-0.09, 0.09] | 0.957 |  |  |
| Sex * APOE4 heterozygous | 0.07 [-1.27, 1.41] | 0.919 |  |  |
| Sex * APOE4 homozygous | -0.69 [-4.46, 3.09] | 0.722 |  |  |
| Average acceleration * Sex * APOE4 heterozygous | -0.01 [-0.05, 0.04] | 0.771 |  |  |
| Average acceleration * Sex * APOE4 homozygous | 0.03 [-0.10, 0.16] | 0.672 |  |  |
| Bipolar |  |  | -0.54 [-1.73, 0.64] | 0.366 |
| Recurrent depression |  |  | 0.12 [-0.15, 0.40] | 0.371 |
| Ethnicity Asian |  |  | 1.51 [0.89, 2.14] | **<0.001** |
| Ethnicity Black |  |  | 1.84 [0.86, 2.83] | **<0.001** |
| Ethnicity Mixed |  |  | 0.73 [0.11, 1.36] | **0.022** |
| Has smoked |  |  | 0.13 [0.00, 0.25] | 0.051 |
| Secondary education |  |  | -1.29 [-1.70, -0.89] | **<0.001** |
| Higher education |  |  | -2.03 [-2.41, -1.65] | **<0.001** |
| CVD diagnosis |  |  | 0.22 [0.09, 0.36] | **0.001** |
| Rare alcohol intake |  |  | -0.22 [-0.50, 0.07] | 0.134 |
| Frequent alcohol intake |  |  | -0.36 [-0.62, -0.10] | **0.007** |
| Sleep less than 7 hours |  |  | 0.14 [-0.01, 0.28] | 0.069 |
| Sleep more than 9 hours |  |  | 0.88 [0.33, 1.44] | **0.002** |
| Diabetes diagnosis |  |  | 0.44 [0.15, 0.73] | **0.003** |
| BMI obese |  |  | 0.33 [0.14, 0.52] | **<0.001** |
| BMI overweight |  |  | 0.05 [-0.09, 0.19] | 0.505 |
| BMI severe obese |  |  | 0.02 [-0.60, 0.64] | 0.945 |
| BMI underweight |  |  | 0.71 [0.05, 1.37] | **0.034** |
| Age |  |  | 0.18 [0.17, 0.19] | **<0.001** |

Supplementary Table 47. Results from the sensitivity analysis linear regression where duration to complete the TMT-B was modelled by IPAQ group, ‘best case’ data was imputed for missing covariates.

|  | Model one | | Model two | |
| --- | --- | --- | --- | --- |
|  | Estimate | p | Estimate | p |
| IPAQ high | 0.41 [0.10, 0.72] | **0.010** | 0.51 [0.34, 0.69] | **<0.001** |
| IPAQ moderate | 0.07 [-0.23, 0.37] | 0.669 | 0.28 [0.12, 0.46] | **0.001** |
| Sex | -0.01 [-0.37, 0.35] | 0.957 | -0.05 [-0.17, 0.09] | 0.459 |
| APOE4 heterozygous | -0.01 [-0.53, 0.51] | 0.967 | 0.08 [-0.06, 0.23] | 0.254 |
| APOE4 homozygous | 0.63 [-0.85, 2.11] | 0.406 | 0.03 [-0.38, 0.46] | 0.893 |
| IPAQ high*Sex | 0.29 [-0.14, 0.73] | 0.186 |  |  |
| IPAQ moderate*Sex | 0.36 [-0.06, 0.79] | 0.095 |  |  |
| IPAQ high * APOE4 heterozygous | 0.10 [-0.53, 0.73] | 0.750 |  |  |
| IPAQ moderate * APOE4 heterozygous | 0.04 [-0.58, 0.66] | 0.894 |  |  |
| IPAQ high * APOE4 homozygous | -1.17 [-2.99, 0.65] | 0.210 |  |  |
| IPAQ moderate * APOE4 homozygous | -1.02 [-2.79, 0.76] | 0.261 |  |  |
| Sex * APOE4 heterozygous | -0.08 [-0.82, 0.67] | 0.841 |  |  |
| Sex * APOE4 homozygous | -1.55 [-3.53, 0.42] | 0.122 |  |  |
| IPAQ high * Sex * APOE4 heterozygous | -0.19 [-1.09, 0.72] | 0.681 |  |  |
| IPAQ moderate * Sex * APOE4 heterozygous | 0.07 [-0.82, 0.97] | 0.871 |  |  |
| IPAQ high * Sex * APOE4 homozygous | 2.95 [0.46, 5.44] | **0.020** |  |  |
| IPAQ moderate * Sex * APOE4 homozygous | 1.48 [-0.97, 3.93] | 0.235 |  |  |
| Bipolar |  |  | -0.56 [-1.57, 0.80] | 0.435 |
| Recurrent depression |  |  | 0.11 [0.06, 0.37] | **<0.001** |
| Ethnicity Asian |  |  | 1.49 [0.87, 2.12] | **<0.001** |
| Ethnicity Black |  |  | 1.83 [0.69, 2.28] | **<0.001** |
| Ethnicity Mixed |  |  | 0.69 [0.05, 1.31] | **0.030** |
| Has smoked |  |  | 0.13 [0.00, 0.25] | **0.045** |
| Secondary education |  |  | -1.29 [-1.64, -0.87] | **<0.001** |
| Higher education |  |  | -2.03 [-2.37, -1.66] | **<0.001** |
| CVD diagnosis |  |  | 0.22 [0.08, 0.35] | **0.001** |
| Rare alcohol intake |  |  | -0.22 [-0.51, 0.06] | 0.127 |
| Frequent alcohol intake |  |  | -0.36 [-0.62, -0.10] | **0.007** |
| Sleep less than 7 hours |  |  | 0.14 [0.00, 0.29] | 0.062 |
| Sleep more than 9 hours |  |  | 0.89 [0.31, 1.42] | **0.002** |
| Diabetes diagnosis |  |  | 0.45 [0.16, 0.74] | **0.002** |
| BMI obese |  |  | 0.34 [0.15, 0.52] | **<0.001** |
| BMI overweight |  |  | 0.04 [-0.10, 0.17] | 0.552 |
| BMI severe obese |  |  | 0.04 [-0.59, 0.64] | 0.887 |
| BMI underweight |  |  | 0.74 [0.08, 1.39] | **0.027** |
| Age |  |  | 0.18 [0.17, 0.18] | **<0.001** |

Supplementary Table 48. Results from the sensitivity analysis linear regression where mean time to correctly identify matches was modelled by average acceleration, ‘best case’ data was imputed for missing covariates.

|  | Model one | | | Model two | |
| --- | --- | --- | --- | --- | --- |
|  | Estimate | p | Estimate | | p |
| Average acceleration | -1.84 [-2.24, -1.43] | **<0.001** | -0.44 [-0.68, -0.19] | | **<0.001** |
| Sex | -36.06 [-52.46, -19.66] | **<0.001** | -23.60 [-27.00, -20.20] | | **<0.001** |
| APOE4 heterozygous | -30.02 [-53.91, -6.12] | **0.014** | -2.24 [-6.12, 1.63] | | 0.257 |
| APOE4 homozygous | -61.31 [-130.83, 6.21] | 0.075 | 5.23 [-5.96, 16.41] | | 0.359 |
| Average acceleration*Sex | 0.62 [0.06, 1.18] | **0.031** |  | |  |
| Average acceleration*APOE4 heterozygous | 0.97 [0.18, 1.77] | **0.016** |  | |  |
| Average acceleration* APOE4 homozygous | 1.93 [-0.34, 4.20] | 0.096 |  | |  |
| Sex * APOE4 heterozygous | 2.13 [-31.97, 36.22] | 0.903 |  | |  |
| Sex * APOE4 homozygous | 67.69 [-28.24, 163.61] | 0.167 |  | |  |
| Average acceleration * Sex * APOE4 heterozygous | -0.19 [-1.35, 0.97] | 0.745 |  | |  |
| Average acceleration * Sex * APOE4 homozygous | -1.76 [-5.03, 1.50] | 0.290 |  | |  |
| Bipolar |  |  | -10.97 [-42.42, 20.47] | | 0.494 |
| Recurrent depression |  |  | -5.28 [-12.51, 1.95] | | 0.152 |
| Ethnicity Asian |  |  | 25.30 [8.61, 41.98] | | **0.003** |
| Ethnicity Black |  |  | 50.20 [24.06, 76.35] | | **<0.001** |
| Ethnicity Mixed |  |  | 13.92 [-2.82, 30.65] | | 0.103 |
| Has smoked |  |  | 1.69 [-1.68, 5.06] | | 0.326 |
| Secondary education |  |  | -12.57 [-23.31, -1.83] | | **0.022** |
| Higher education |  |  | -19.94 [-30.00, -9.88] | | **<0.001** |
| CVD diagnosis |  |  | -0.31 [-3.88, 3.27] | | 0.866 |
| Rare alcohol intake |  |  | 4.25 [-3.26, 11.77] | | 0.267 |
| Frequent alcohol intake |  |  | 0.47 [-6.52, 7.47] | | 0.895 |
| Sleep less than 7 hours |  |  | -0.97 [-4.85, 2.90] | | 0.623 |
| Sleep more than 9 hours |  |  | 10.65 [-4.08, 25.39] | | 0.156 |
| Diabetes diagnosis |  |  | 3.07 [-4.66, 10.81] | | 0.436 |
| BMI obese |  |  | 3.86 [-1.19, 8.91] | | 0.134 |
| BMI overweight |  |  | -0.17 [-3.52, 3.86] | | 0.929 |
| BMI severe obese |  |  | -8.59 [-25.09, 7.90] | | 0.307 |
| BMI underweight |  |  | 17.60 [0.06, 35.15] | | **0.049** |
| Age |  |  | 3.59 [3.46, 3.83] | | **<0.001** |

Supplementary Table 49. Results from the sensitivity analysis linear regression where mean time to correctly identify matches was modelled by IPAQ group, ‘best case’ data was imputed for missing covariates.

|  | Model one | | Model two | |
| --- | --- | --- | --- | --- |
|  | Estimate | p value | Estimate | p value |
| IPAQ high | -1.18 [-9.11, 6.75] | 0.771 | -1.52 [-6.12, 3.09] | 0.519 |
| IPAQ moderate | -4.21 [-11.88, 3.45] | 0.281 | -0.72 [-5.24, 3.79] | 0.753 |
| Sex | -22.17 [-31.25, -13.08] | **<0.001** | -23.42 [-26.82, -20.01] | **<0.001** |
| APOE4 heterozygous | -5.45 [-18.78, 7.89] | 0.423 | -2.32 [-6.20, 1.56] | 0.241 |
| APOE4 homozygous | -8.55 [-46.47, 29.36] | 0.658 | 5.27 [-5.92, 16.46] | 0.356 |
| IPAQ high*Sex | 4.18 [-6.97, 15.33] | 0.463 |  |  |
| IPAQ moderate*Sex | 7.70 [-3.20, 18.61] | 0.166 |  |  |
| IPAQ high * APOE4 heterozygous | 2.59 [-13.54, 18.71] | 0.753 |  |  |
| IPAQ moderate * APOE4 heterozygous | 4.21 [-11.59, 20.00] | 0.601 |  |  |
| IPAQ high * APOE4 homozygous | -10.69 [-57.30, 35.92] | 0.653 |  |  |
| IPAQ moderate * APOE4 homozygous | 13.08 [-32.27, 58.43] | 0.572 |  |  |
| Sex * APOE4 heterozygous | 0.26 [-18.82, 19.33] | 0.979 |  |  |
| Sex * APOE4 homozygous | 33.32 [-17.05, 83.68] | 0.195 |  |  |
| IPAQ high * Sex * APOE4 heterozygous | -5.66 [-28.78, 17.47] | 0.632 |  |  |
| IPAQ moderate * Sex * APOE4 heterozygous | -3.48 [-26.33, 19.38] | 0.765 |  |  |
| IPAQ high * Sex * APOE4 homozygous | 2.31 [-61.27, 65.88] | 0.943 |  |  |
| IPAQ moderate * Sex * APOE4 homozygous | -43.11 [-105.61, 19.39] | 0.176 |  |  |
| Bipolar |  |  | -11.18 [-42.64, 20.29] | 0.486 |
| Recurrent depression |  |  | -4.91 [-12.14, 2.33] | 0.184 |
| Ethnicity Asian |  |  | 25.42 [8.73, 42.12] | **0.003** |
| Ethnicity Black |  |  | 50.50 [24.33, 76.66] | **<0.001** |
| Ethnicity Mixed |  |  | 14.44 [-2.31, 31.18] | 0.091 |
| Has smoked |  |  | 1.62 [-1.75, 5.00] | 0.346 |
| Secondary education |  |  | -12.23 [-22.98, -1.48] | **0.026** |
| Higher education |  |  | -19.52 [-29.59, -9.45] | **<0.001** |
| CVD diagnosis |  |  | -0.07 [-3.65, 3.50] | 0.969 |
| Rare alcohol intake |  |  | 4.64 [-2.88, 12.15] | 0.227 |
| Frequent alcohol intake |  |  | 0.41 [-6.59, 7.41] | 0.909 |
| Sleep less than 7 hours |  |  | -1.06 [-4.94, 2.82] | 0.592 |
| Sleep more than 9 hours |  |  | 11.16 [-3.58, 25.91] | 0.138 |
| Diabetes diagnosis |  |  | 3.85 [-3.88, 11.59] | 0.329 |
| BMI obese |  |  | 5.43 [0.45, 10.41] | **0.033** |
| BMI overweight |  |  | 0.91 [-2.76, 4.58] | 0.627 |
| BMI severe obese |  |  | -5.63 [-22.05, 10.80] | 0.502 |
| BMI underweight |  |  | 16.80 [-0.75, 34.35] | 0.061 |
| Age |  |  | 3.68 [3.46, 3.92] | **<0.001** |

Supplementary Table 50. Results from the sensitivity analysis linear regression where % correct on the pairs matching task was modelled by average acceleration, ‘best case’ data was imputed for missing covariates.

|  | Model one | | Model two | |
| --- | --- | --- | --- | --- |
|  | Estimate | p | Estimate | p |
| Average acceleration | 0.01 [-0.04, 0.06] | 0.634 | -0.06 [-0.09, -0.03] | **<0.001** |
| Sex | -0.52 [-2.63, 1.58] | 0.625 | 0.47 [0.02, 0.92] | **0.039** |
| APOE4 heterozygous | 2.00 [-1.07, 5.07] | 0.202 | -0.42 [-0.93, 0.10] | 0.112 |
| APOE4 homozygous | -5.12 [-13.92, 3.67] | 0.254 | -0.56 [-2.04, 0.91] | 0.454 |
| Average acceleration*Sex | 0.02 [-0.06, 0.09] | 0.667 |  |  |
| Average acceleration*APOE4 heterozygous | -0.09 [-0.19, 0.01] | 0.076 |  |  |
| Average acceleration* APOE4 homozygous | 0.19 [-0.10, 0.48] | 0.198 |  |  |
| Sex * APOE4 heterozygous | -1.16 [-5.54, 3.22] | 0.604 |  |  |
| Sex * APOE4 homozygous | 6.64 [-5.67, 18.95] | 0.291 |  |  |
| Average acceleration * Sex * APOE4 heterozygous | 0.07 [-0.08, 0.22] | 0.373 |  |  |
| Average acceleration * Sex * APOE4 homozygous | -0.28 [-0.70, 0.13] | 0.184 |  |  |
| Bipolar |  |  | -0.97 [-5.12, 3.19] | 0.648 |
| Recurrent depression |  |  | -0.30 [-1.26, 0.66] | 0.538 |
| Ethnicity Asian |  |  | -3.38 [-5.58, -1.17] | **0.003** |
| Ethnicity Black |  |  | -5.76 [-9.22, -2.30] | **0.001** |
| Ethnicity Mixed |  |  | -2.76 [-4.97, -0.55] | **0.015** |
| Has smoked |  |  | -0.35 [-0.80, 0.10] | 0.123 |
| Secondary education |  |  | 1.53 [0.11, 2.95] | **0.035** |
| Higher education |  |  | 2.27 [0.94, 3.60] | **<0.001** |
| CVD diagnosis |  |  | -0.15 [-0.62, 0.32] | 0.540 |
| Rare alcohol intake |  |  | 0.91 [-0.08, 1.90] | 0.072 |
| Frequent alcohol intake |  |  | 0.59 [-0.33, 1.52] | 0.210 |
| Sleep less than 7 hours |  |  | -0.08 [-0.59, 0.44] | 0.768 |
| Sleep more than 9 hours |  |  | -1.39 [-3.34, 0.55] | 0.161 |
| Diabetes diagnosis |  |  | -0.96 [-1.98, 0.07] | 0.067 |
| BMI obese |  |  | 0.14 [-0.53, 0.80] | 0.691 |
| BMI overweight |  |  | 0.09 [-0.40, 0.58] | 0.710 |
| BMI severe obese |  |  | -0.46 [-2.64, 1.72] | 0.681 |
| BMI underweight |  |  | -2.43 [-4.75, -0.11] | **0.040** |
| Age |  |  | -0.28 [-0.31, -0.25] | **<0.001** |

Supplementary Table 51. Results from the sensitivity analysis linear regression where % correct on the pairs matching task was modelled by IPAQ group, ‘best case’ data was imputed for missing covariates.

|  | Model one | | Model two | |
| --- | --- | --- | --- | --- |
|  | Estimate | p | Estimate | p |
| IPAQ high | -1.19 [-2.20, -0.17] | **0.022** | -0.37 [-0.98, 0.24] | 0.238 |
| IPAQ moderate | -0.34 [-1.31, 0.64] | 0.602 | -0.04 [-0.64, 0.56] | 0.893 |
| Sex | -0.12 [-1.28, 1.04] | 0.836 | 0.51 [0.06, 0.95] | **0.028** |
| APOE4 heterozygous | -2.28 [-3.98, -0.58] | **0.009** | -0.42 [-0.93, 0.09] | 0.107 |
| APOE4 homozygous | -2.03 [-6.86, 2.81] | 0.412 | -0.56 [-2.04, 0.92] | 0.459 |
| IPAQ high*Sex | 0.37 [-1.05, 1.80] | 0.606 |  |  |
| IPAQ moderate*Sex | -0.25 [-1.64, 1.14] | 0.722 |  |  |
| IPAQ high * APOE4 heterozygous | 2.07 [0.01, 4.13] | **0.048** |  |  |
| IPAQ moderate * APOE4 heterozygous | 1.80 [-0.22, 3.81] | 0.080 |  |  |
| IPAQ high * APOE4 homozygous | 3.40 [-2.55, 9.34] | 0.263 |  |  |
| IPAQ moderate * APOE4 homozygous | 2.88 [-2.91, 8.66] | 0.329 |  |  |
| Sex * APOE4 heterozygous | 1.34 [-1.10, 3.77] | 0.281 |  |  |
| Sex * APOE4 homozygous | 1.77 [-4.65, 8.20] | 0.589 |  |  |
| IPAQ high * Sex * APOE4 heterozygous | -0.57 [-3.52, 2.38] | 0.705 |  |  |
| IPAQ moderate * Sex * APOE4 heterozygous | -0.59 [-3.51, 2.32] | 0.689 |  |  |
| IPAQ high * Sex * APOE4 homozygous | -4.64 [-12.57, 3.65] | 0.281 |  |  |
| IPAQ moderate * Sex * APOE4 homozygous | -4.12 [-12.09, 3.86] | 0.312 |  |  |
| Bipolar |  |  | -0.99 [-5.15, 3.16] | 0.639 |
| Recurrent depression |  |  | -0.25 [-1.12 0.70] | 0.603 |
| Ethnicity Asian |  |  | -3.36 [-5.56, -1.15] | **0.003** |
| Ethnicity Black |  |  | -5.71 [-9.17, -2.25] | **0.001** |
| Ethnicity Mixed |  |  | -2.66 [-4.87, -0.45] | **0.019** |
| Has smoked |  |  | -0.36 [-0.80, 0.09] | 0.116 |
| Secondary education |  |  | 1.56 [0.14, 2.98] | **0.031** |
| Higher education |  |  | 2.31 [0.98, 3.64] | **<0.001** |
| CVD diagnosis |  |  | -0.12 [-0.59, 0.36] | 0.627 |
| Rare alcohol intake |  |  | 0.96 [-0.03, 1.95] | 0.058 |
| Frequent alcohol intake |  |  | 0.58 [-0.35, 1.50] | 0.221 |
| Sleep less than 7 hours |  |  | -0.08 [-0.60, 0.43] | 0.743 |
| Sleep more than 9 hours |  |  | -1.32 [-3.27, 0.63] | 0.184 |
| Diabetes diagnosis |  |  | -0.86 [-1.88, 0.16] | 0.099 |
| BMI obese |  |  | 0.34 [-0.32, 0.99] | 0.318 |
| BMI overweight |  |  | 0.19 [-0.29, 0.68] | 0.440 |
| BMI severe obese |  |  | -0.08 [-2.25, 2.09] | 0.940 |
| BMI underweight |  |  | -2.53 [-4.85, -0.21] | **0.032** |
| Age |  |  | -0.27 [-0.30, -0.24] | **<0.001** |

**REFERENCES**

1. Meng XF, Yu JT, Wang HF, et al. Midlife vascular risk factors and the risk of Alzheimer's disease: a systematic review and meta-analysis. *J Alzheimers Dis* 2014; 42: 1295-1310.
2. Alzheimer’s Society. Risk Factors For Dementia, https://www.alzheimers.org.uk/about-dementia/managing-the-risk-of-dementia/risk-factors-for-dementia#:~:text=Race%20and%20ethnicity&text=A%20few%20studies%20have%20suggested,people%20from%20White%20ethnic%20groups. (2023, accessed May 12, 2024)
3. Nourhashémi F, Gillette-Guyonnet S, Andrieu S, et al. Alzheimer disease: protective factors. *Am J Clin Nutr* 2000; 71: 643S-649S.
4. Rehm J, Hasan OS, Black SE, et al. Alcohol use and dementia: a systematic scoping review. *Alzheimers Res Ther* 2019; 11: 1-11.
5. Kim JW, Lee DY, Lee BC, et al. Alcohol and cognition in the elderly: a review. *Psychiatry Invest* 2012; 9: 8.
6. Ma Y, Ajnakina O, Steptoe A, et al. Higher risk of dementia in English older individuals who are overweight or obese. *Int J Epidemiol* 2020; 49: 1353-1365.
7. Alzheimer’s Society. Depression and dementia risk, https://www.alzheimers.org.uk/about-dementia/managing-the-risk-of-dementia/reduce-your-risk-of-dementia/depression. (2023, accessed May 12, 2024)
8. Gorospe EC and Dave JK. The risk of dementia with increased body mass index. *Age Ageing* 2007; 36: 23-29.
