# Supplementary material for: The Relationship Between Physical Activity and Non-Modifiable Risk Factors on Alzheimer’s Disease and Brain Health Markers: A UK Biobank Study
Source: J Alzheimers Dis. 2024 Oct 8;101(4):1029–42. doi: 10.3233/JAD-240269 (PMC11492105; doi:10.3233/JAD-240269)
Supplement: Supplementary Material 3 [file jad-101-jad240269-s003.docx]

**Supplementary Material Missing Data**

There were 2,788 participants that were excluded from analysis due to missing covariate data (e.g., alcohol intake, sleep duration etc.). This is 4.04% of the total 69,060 participants that were eligible for inclusion in the analysis.

Supplementary Table 3: Descriptive statistics for included participants and participants excluded due to missing covariate data, presented with a statistical test of differences.

|  | **Total AD incidence group (included participants) (N=69,060) N(%)** | **Total ineligible participants (N=2,788) N(%)** | **Test of differences** | |
| --- | --- | --- | --- | --- |
| **Sex** |  |  |  | |
| Male | 31326 (45.36) | 1345 (48.24) | χ^2^(1) = 8.98, *p*=0.003 | |
| Female | 37734 (54.64) | 1443 (51.76) |  |  |
|  |  |  |  | |
| **Ethnicity** |  |  |  | |
| White | 67022 (97.05) | 2459 (88.20) | χ^2^(3) = 47.88, *p*<0.001 | |
| Asian | 796 (1.15) | 46 (1.65) |  |  |
| Black | 493 (0.71) | 39 (1.40) |  |  |
| Mixed | 749 (1.08) | 51 (1.83) |  |  |
| Missing |  | 193 |  |  |
|  |  |  |  | |
| **APOE genotype** |  |  |  | |
| Non-carrier | 50983 (73.82) | 2122 (76.11) | χ^2^(2) = 7.29, *p*=0.026 | |
| Heterozygous carrier | 16475 (23.86) | 608 (21.81) |  |  |
| Homozygous carrier | 1602 (2.32) | 58 (2.08) |  |  |
|  |  |  |  | |
| **Overall acceleration average (mg), Mean (SD)** | 27.59 (7.21) | 26.72 (7.20) | t(71846) = 6.25, *p*<0.001 | |
|  |  |  |  | |
| **IPAQ group** |  |  |  | |
| Low | 12330 (17.85) | 505 (18.11) | χ^2^(2) = 5.69, *p*=0.058 | |
| Moderate | 27097 (42.91) | 1148 (41.18) |  |  |
| High | 29633 (39.24) | 1135 (40.71) |  |  |
|  |  |  |  | |
| **Age (at follow up), Mean (SD)** | 62.28 (7.84) | 62.56 (7.97) | t(71846) = 1.85, *p*=0.065 | |
|  |  |  |  | |
| **Depression/Bipolar status** |  |  |  | |
| No depression or single depressive episode | 64787 (93.81) | 2637 (94.58) | χ^2^(2) = 5.55, *p*=0.062 | |
| Bipolar | 204 (0.30) | 12 (0.43) |  |  |
| Recurrent depression | 4069 (5.89) | 139 (4.99) |  |  |
|  |  |  |  | |
| **Smoking status** |  |  |  | |
| No | 26621 (38.55) | 996 (35.72) | χ^2^(1) = 2.87, *p*=0.090 | |
| Yes | 42439 (61.45) | 1701 (61.01) |  |  |
| Missing |  | 91 |  |  |
|  |  |  |  | |
| **Level of education** |  |  |  | |
| None | 4862 (7.04) | 217 (7.78) | χ^2^(2) = 9.44, *p*=0.009 | |
| Secondary (GCSE/A level) | 13703 (19.84) | 492 (17.65) |  |  |
| Higher (University degree/professional qualification) | 50495 (73.12) | 1802 (64.63) |  |  |
| Missing |  | 277 |  | |
|  |  |  |  | |
| **CVD diagnosis** |  |  |  | |
| No | 45123 (65.34) | 1010 (36.23) | χ^2^(1) = 988.29, *p*<0.001 | |
| Yes | 23937 (34.66) | 1778 (63.77) |  |  |
|  |  |  |  | |
| **Diabetes diagnosis** |  |  |  | |
| No | 66155 (95.79) | 2545 (91.28) | χ^2^(1) = 6.01, *p*=0.014 | |
| Yes | 2905 (4.21) | 139 (4.99) |  |  |
| Missing |  | 104 |  | |
|  |  |  |  | |
| **Frequency of alcohol intake** |  |  |  | |
| None | 4069 (5.89) | 191 (6.85) | χ^2^(2) = 6.89, *p*=0.032 | |
| Rarely (special occasions/1-3x per month) | 14211 (20.58) | 595 (21.34) |  |  |
| Frequently (more than 1/2x per week) | 50780 (73.53) | 1984 (71.16) |  |  |
| Missing |  | 18 |  |  |
|  |  |  |  | |
| **Sleep duration** |  |  |  | |
| Less than 7 hours | 15327 (22.19) | 682 (24.46) | χ^2^(2) = 17.71, *p<*0.001 | |
| 7 – 9 hours | 52897 (76.60) | 1979 (70.98) |  |  |
| More than 9 hours | 836 (1.21) | 43 (1.54) |  |  |
| Missing |  | 84 |  | |
|  |  |  |  | |
| **Social activity** |  |  |  | |
| Never/no friends/family outside household | 931 (1.35) | 46 (1.65) | χ^2^(2) = 6.52, *p*=0.038 | |
| Rarely (once every few months/once a month) | 14994 (21.70) | 611 (21.92) |  |  |
| Frequently (more than once a week) | 53145 (76.95) | 1981 (71.05) |  |  |
| Missing |  | 150 |  |  |
|  |  |  |  | |
| **BMI** **(kg/m^2^) (Mean, SD)** |  |  |  | |
| Underweight (>18.5) | 417 (0.60) | 16 (0.57) | χ^2^(4) = 9.40, *p*=0.052 | |
| Healthy (<=18.5 & <25) | 26852 (38.88) | 994 (35.65) |  |  |
| Overweight (<=25 & <30) | 28502 (41.27) | 1098 (39.38) |  |  |
| Obese (<=30 & <40) | 12367 (17.91) | 537 (19.26) |  |  |
| Severely obese (<=40) | 922 (1.34) | 41 (1.47) |  |  |
| Missing |  | 102 |  |  |
|  |  |  |  | |
| **IMD**, Mean (SD) | 14.59 (11.82) | 17.25 (13.26) | t(71846) = 11.59, *p*<0.001 | |
|  |  |  |  | |
| **AD incidence** | 141 (0.20) | 5 (0.20) | χ^2^(1) = 0.08, *p*=0.775 | |
|  |  |  |  | |
| **Time to follow up (years), Mean (SD)** | 13.86 (0.84) | 14.02 (0.85) | t(71846) = 9.86, *p*<0.001 | |
|  |  |  |  | |
| **Brain volume (mm^3^), Mean (SD)** | **For brain volume population only** | | | |
| Volume of ventricular CSF | 47437.29 (20523.04) | 48187.67 (19946.49) | | t(13509) = 0.73, *p*=0.463 |
| Total brain volume (grey and white matter) | 1162172.00 (110413.10) | 1160799.00 (113171.10) | | t(13509) = 0.25, *p*=0.803 |
|  |  |  | |  |
|  | **For cognition population only** | | | |
| **Cognitive function, Mean (SD)** |  |  | |  |
| Duration to complete TMT-B (s) | 526.42 (164.69) | 550.69 (182.63) | | t(10463) = 2.73, *p*=0.006 |
| Reaction time (ms) | 585.59 (89.72) | 591.81 (90.52) | | t(10463) = 1.31, *p*=0.190 |
| Pairs matching (% correct) | 80.44 (11.38) | 78.86 (12.17) | | t(10463) = 2.58, *p*=0.010 |
| Numeric memory (% correct) | 82.36 (6.25) | 81.55 (6.85) | | t(10463) = 2.40, *p*=0.016 |
| SDST (% correct) | 95.77 (8.20) | 94.83 (11.68) | | t(10463) = 2.09, *p*=0.036 |

Values given are N(%), unless otherwise stated in the row header. Some numbers do not reflect Figure 1, as some participants were missing data for multiple covariates but were only excluded for one reason in figure 1. χ^2^ tests conducted excluding participants with missing data for that variable. In Fig. 1, exclusion criteria for brain volume and cognitive test scores were applied last; however, here they were applied first to ensure missing data accurately reflected missing covariate data only.
