# Supplementary material for: The Relationship Between Physical Activity and Non-Modifiable Risk Factors on Alzheimer’s Disease and Brain Health Markers: A UK Biobank Study
Source: J Alzheimers Dis. 2024 Oct 8;101(4):1029–42. doi: 10.3233/JAD-240269 (PMC11492105; doi:10.3233/JAD-240269)
Supplement: Supplementary Material 4 [file jad-101-jad240269-s004.docx]

**Results**

**Assumptions testing**

Assumptions testing was completed for all models. All assumptions were met, except for the variable ‘frequency of social visits’ for the assumption of multicollinearity, VIF >10, and the variable ‘IMD score’ for the assumption of a linear relationship between the outcomes and predictor. As a result, these variables were removed from the full models. The distribution for the duration to complete the TMT-B was non-normal and was improved with a square root transformation prior to analysis. There were some violations of the Durbin Watson test and non-constant variance test for the regressions with cognitive outcomes, though these were considered acceptable.

**Model statistics**

Supplementary Table 4. Results from the logistic regression where AD incidence was modelled with average acceleration.

|  | Model one | | | Model two | | | | |
| --- | --- | --- | --- | --- | --- | --- | --- | --- |
|  | Estimate | Odds ratio [95% CI] | p | | Estimate | Odds ratio [95% CI] | p |  |
| Average acceleration | -0.10 [-0.17, -0.04] | 0.90 [0.84, 0.96] | **0.002** | | -0.04 [-0.07, -0.02] | 0.96 [0.93, 0.98] | **0.002** |  |
| Sex | -0.76 [-2.82, 1.32] | 0.47 [0.06, 3.75] | 0.469 | | 0.27 [-0.08, 0.63] | 1.31 [0.92, 1.87] | 0.137 |  |
| APOE4 heterozygous | 1.21 [-0.94, 3.38] | 3.36 [0.39, 29.43] | 0.271 | | 1.44 [1.08, 1.80] | 4.21 [2.93, 6.07] | **<0.001** |  |
| APOE4 homozygous | 3.18 [0.13, 6.16] | 24.07 [1.14, 471.26] | **0.038** | | 2.72 [2.18, 3.22] | 15.14 [8.84, 25.01] | **<0.001** |  |
| Average acceleration*Sex | 0.05 [-0.04, 0.13] | 1.05 [0.97, 1.14] | 0.261 | |  |  |  |  |
| Average acceleration*APOE4 heterozygous | 0.01 [-0.08, 0.09] | 1.01 [0.92, 1.10] | 0.881 | |  |  |  |  |
| Average acceleration* APOE4 homozygous | -0.02 [-0.14, 0.10] | 0.98 [0.87, 1.11] | 0.771 | |  |  |  |  |
| Sex * APOE4 heterozygous | 0.39 [-2.41, 3.19] | 1.48 [0.09, 24.19] | 0.783 | |  |  |  |  |
| Sex * APOE4 homozygous | 0.49 [-3.46, 4.44] | 1.63 [0.03, 85.11] | 0.807 | |  |  |  |  |
| Average acceleration * Sex * APOE4 heterozygous | -0.02 [-0.13, 0.10] | 0.98 [0.88, 1.10] | 0.759 | |  |  |  |  |
| Average acceleration * Sex * APOE4 homozygous | -0.04 [-0.20, 0.13] | 0.97 [0.82, 1.14] | 0.672 | |  |  |  |  |
| Bipolar |  |  |  | | 1.00 [-1.91, 2.61] | 2.73 [0.15, 13.64] | 0.338 |  |
| Recurrent depression |  |  |  | | -0.69 [-2.09, 0.29] | 0.50 [0.12, 1.34] | 0.241 |  |
| Ethnicity Asian |  |  |  | | 1.14 [-0.29, 2.17] | 3.13 [0.75, 8.77] | 0.060 |  |
| Ethnicity Black |  |  |  | | 0.61 [-2.27, 2.17] | 1.85 [ 0.10, 8.79] | 0.549 |  |
| Ethnicity Mixed |  |  |  | | 0.30 [-2.57, 1.82] | 1.35 [0.08, 6.17] | 0.766 |  |
| Has smoked |  |  |  | | 0.06 [-0.29, 0.43] | 1.06 [ 0.75, 1.54] | 0.733 |  |
| Secondary education |  |  |  | | -0.53 [-1.06, 0.01] | 0.59 [0.35, 1.01] | 0.051 |  |
| Higher education |  |  |  | | -0.71 [-1.13, -0.26] | 0.49 [ 0.32, 0.77] | **0.001** |  |
| CVD diagnosis |  |  |  | | -0.20 [-0.55, 0.16] | 0.82 [0.58, 1.17] | 0.276 |  |
| Rare alcohol intake |  |  |  | | -0.88 [-1.48, -0.27] | 0.41 [0.23, 0.76] | **0.004** |  |
| Frequent alcohol intake |  |  |  | | -0.86 [-1.35, -0.32] | 0.42 [0.26, 0.72] | **0.001** |  |
| Sleep less than 7 hours |  |  |  | | 0.25 [-0.14, 0.62] | 1.29 [0.87, 1.87] | 0.193 |  |
| Sleep more than 9 hours |  |  |  | | -0.87 [-3.74, 0.65] | 0.42 [0.02, 1.91] | 0.389 |  |
| Diabetes diagnosis |  |  |  | | -0.04 [-0.83, 0.62] | 0.96 [0.44, 1.86] | 0.906 |  |
| BMI obese |  |  |  | | -0.32 [-0.85, 0.18] | 0.72 [0.43, 1.19] | 0.217 |  |
| BMI overweight |  |  |  | | -0.28 [-0.67, 0.10] | 0.75 [0.51, 1.11] | 0.146 |  |
| BMI severe obese |  |  |  | | 0.67 [-0.58, 1.65] | 1.96 [0.56, 5.20] | 0.225 |  |
| BMI underweight |  |  |  | | -12.05 [-121.28, -3.22] | 0.00 [0.00, 0.04] | 0.966 |  |
| Age |  |  |  | | 0.19 [0.15, 0.22] | 1.20 [1.16, 1.25] | **<0.001** |  |

NB: Model one significantly predicted AD incidence, F(11, 69059) = 146.96, p<0.001. It accounted for 7.34% of the total variance (Nagelkerke R^2^). Model two also significantly predicted AD incidence, F(23,69059) = 331.06, p<0.001. It accounted for 16.52% of the total variance (Nagelkerke R^2^). Compared to the previous model it was significantly better at predicting the outcome, F(12, 69036) = 184.10, p<0.001.

Supplementary Table 5. Results from the Poisson regression where AD incidence was modelled by average acceleration.

|  | Model one | | | | Model two | | |
| --- | --- | --- | --- | --- | --- | --- | --- |
|  | Estimate | Risk ratio | p | Estimate | | Risk ratio | p |
| Average acceleration | -0.10 [-0.17, -0.04] | 0.90 [0.85, 0.96] | **0.003** | -0.04 [-0.07, -0.01] | | 0.96 [0.93, 0.99] | **0.002** |
| Sex | -0.76 [-2.81, 1.32] | 0.47 [0.06, 3.74] | 0.469 | .024 [-0.09, 0.62] | | 1.31 [0.92, 1.86] | 0.138 |
| APOE4 heterozygous | 1.21 [-0.93, 3.37] | 3.35 [0.39, 29.19] | 0.271 | 1.43 [1.07, 1.79] | | 4.17 [2.91. 5.98] | **<0.001** |
| APOE4 homozygous | 3.13 [0.10, 6.04] | 22.77 [1.11, 421.39] | **0.039** | 2.68 [2.17, 3.19] | | 14.61 [8.79, 24.28] | **<0.001** |
| Average acceleration*Sex | 0.05 [-0.04, 0.13] | 1.05 [0.97, 1.14] | 0.262 |  | |  |  |
| Average acceleration*APOE4 heterozygous | 0.01 [-0.08, 0.09] | 1.01 [0.92, 1.10] | 0.880 |  | |  |  |
| Average acceleration* APOE4 homozygous | -0.02 [-0.14, 0.10] | 0.98 [0.87, 1.11] | 0.789 |  | |  |  |
| Sex * APOE4 heterozygous | 0.38 [-2.42, 3.17] | 1.46 [0.09, 23.73] | 0.789 |  | |  |  |
| Sex * APOE4 homozygous | 0.49 [-3.40, 4.39] | 1.63 [0.03, 80.59] | 0.805 |  | |  |  |
| Average acceleration * Sex * APOE4 heterozygous | -0.02 [-0.13, 0.10] | 0.98 [0.88, 1.10] | 0.764 |  | |  |  |
| Average acceleration * Sex * APOE4 homozygous | -0.04 [-0.20, 0.13] | 0.97 [0.82, 1.14] | 0.669 |  | |  |  |
| Bipolar |  |  |  | 1.06 [-0.91, 3.04] | | 2.90 [0.40, 20.93] | 0.292 |
| Recurrent depression |  |  |  | -0.61 [-1.76, 0.53] | | 0.54 [0.17, 1.71] | 0.295 |
| Ethnicity Asian |  |  |  | 1.17 [0.00, 2.35] | | 3.23 [1.00, 10.44] | 0.050 |
| Ethnicity Black |  |  |  | 0.65 [-1.33, 2.63] | | 1.92 [0.26, 13.90] | 0.519 |
| Ethnicity Mixed |  |  |  | 0.31 [-1.66, 2.29] | | 1.37 [0.19, 9.85] | 0.754 |
| Has smoked |  |  |  | 0.06 [-0.30, 0.42] | | 1.06 [0.74, 1.52] | 0.740 |
| Secondary education |  |  |  | -0.52 [-1.05, 0.00] | | 0.59 [0.35, 1.00] | 0.051 |
| Higher education |  |  |  | -0.71 [-1.14, -0.27] | | 0.49 [0.32, 0.76] | **0.001** |
| CVD diagnosis |  |  |  | -0.19 [-0.54, 0.16] | | 0.83 [0.58, 1.17] | 0.285 |
| Rare alcohol intake |  |  |  | -0.87 [-1.47, -0.28] | | 0.42 [0.23, 0.76] | **0.004** |
| Frequent alcohol intake |  |  |  | -0.85 [-1.36, -0.35] | | 0.43 [0.26, 0.71] | **0.001** |
| Sleep less than 7 hours |  |  |  | 0.25 [-0.13, 0.63] | | 1.28 [0.88, 1.87] | 0.195 |
| Sleep more than 9 hours |  |  |  | -0.86 [-2.84, 1.11] | | 0.42 [0.06, 3.04] | 0.391 |
| Diabetes diagnosis |  |  |  | -0.03 [-0.74, 0.67] | | 0.97 [0.48, 1.96] | 0.923 |
| BMI obese |  |  |  | -0.32 [-0.83, 0.19] | | 0.73 [0.44, 1.21] | 0.220 |
| BMI overweight |  |  |  | -0.28 [-0.66, 0.10] | | 0.76 [0.52, 1.11] | 0.149 |
| BMI severe obese |  |  |  | 0.67 [-0.40, 1.74] | | 1.96 [0.67, 5.71] | 0.217 |
| BMI underweight |  |  |  | -12.80 [-809.31, 783.71] | | 0.00 [0.00, Inf] | 0.975 |
| Age |  |  |  | 0.18 [0.15, 0.22] | | 1.20 [1.16, 1.25] | **<0.001** |

NB: Model one could significantly predict AD incidence, F(11, 69153)= 146.12, p<0.001. It accounted for 7.31% of the total variance (Nagelkerke R^2^).

Model two could also significantly predict AD incidence, F(23, 69153) = 327.39, p<0.001. It accounted for 16.35% of the total variance (Nagelkerke R^2^).

Supplementary Table 6. Results from the logistic regression where AD incidence was modelled by IPAQ group.

|  | Model one | | | Model two | | |  |
| --- | --- | --- | --- | --- | --- | --- | --- |
|  | Estimate | Odds ratio | p | Estimate | Odds ratio | p | |
| IPAQ high | -0.16 [-1.13, 0.91] | 0.85 [0.32, 2.48] | 0.754 | -0.32 [-0.76, 0.14] | 0.73 [0.47, 1.15] | 0.164 | |
| IPAQ moderate | -0.95 [-2.11, 0.21] | 0.39 [0.12, 1.24] | 0.101 | -0.46 [-0.91, 0.00] | 0.63 [0.40, 1.00] | **0.047** | |
| Sex | 0.63 [-0.36, 1.71] | 1.88 [0.70, 5.53] | 0.222 | 0.29 [-0.06, 0.65] | 1.34 [0.94, 1.91] | 0.111 | |
| APOE4 heterozygous | 1.29 [0.19, 2.43] | 3.64 [1.21, 11.31] | **0.020** | 1.43 [1.07, 1.80] | 4.19 [2.92, 6.05] | **<0.001** | |
| APOE4 homozygous | 1.79 [-1.15, 3.56] | 5.98 [0.32, 35.31] | 0.099 | 2.71 [2.17, 3.21] | 15.05 [8.79, 24.85] | **<0.001** | |
| IPAQ high*Sex | -0.45 [-1.80, 0.84] | 0.63 [0.17, 2.31] | 0.494 |  |  |  | |
| IPAQ moderate*Sex | 0.06 [-1.41, 1.52] | 1.06 [0.24, 4.58] | 0.936 |  |  |  | |
| IPAQ high * APOE4 heterozygous | -0.21 [-1.61, 1.18] | 0.81 [0.20, 3.24] | 0.768 |  |  |  | |
| IPAQ moderate * APOE4 heterozygous | 0.45 [-1.04, 1.96] | 1.56 [0.35, 7.07] | 0.553 |  |  |  | |
| IPAQ high * APOE4 homozygous | 0.62 [-1.57, 3.72] | 1.87 [0.21, 41.30] | 0.612 |  |  |  | |
| IPAQ moderate * APOE4 homozygous | 1.55 [-0.64, 4.66] | 4.71 [0.53, 105.96] | 0.213 |  |  |  | |
| Sex * APOE4 heterozygous | -0.64 [-2.18, 0.84] | 0.53 [0.11, 2.32] | 0.400 |  |  |  | |
| Sex * APOE4 homozygous | -0.62 [-3.99, 2.73] | 0.54 [0.02, 15.39] | 0.680 |  |  |  | |
| IPAQ high * Sex * APOE4 heterozygous | 0.95 [-0.91, 2.85] | 2.58 [0.40, 17.28] | 0.320 |  |  |  | |
| IPAQ moderate * Sex * APOE4 heterozygous | 0.62 [-1.36, 2.61] | 1.85 [0.26, 13.56] | 0.540 |  |  |  | |
| IPAQ high * Sex * APOE4 homozygous | 0.94 [-2.69, 4.57] | 2.55 [0.07, 96.87] | 0.581 |  |  |  | |
| IPAQ moderate * Sex * APOE4 homozygous | -0.05 [-3.74, 3.64] | 0.95 [0.02, 38.09] | 0.977 |  |  |  | |
| Bipolar |  |  |  | 1.04 [-1.88, 2.66] | 2.84 [0.15, 14.25] | 0.321 | |
| Recurrent depression |  |  |  | -0.68 [-2.08, 0.30] | 0.51 [0.12, 1.35] | 0.248 | |
| Ethnicity Asian |  |  |  | 1.13 [-0.31, 2.16] | 3.09 [0.74, 8.68] | 0.064 | |
| Ethnicity Black |  |  |  | 0.59 [-2.29, 2.15] | 1.81 [0.10, 8.57] | 0.562 | |
| Ethnicity Mixed |  |  |  | 0.25 [-2.63, 1.77] | 1.28 [0.07, 5.85] | 0.807 | |
| Has smoked |  |  |  | 0.07 [-0.29, 0.44] | 1.07 [0.75, 1.55] | 0.703 | |
| Secondary education |  |  |  | -0.53 [-1.06, 0.00] | 0.59 [0.35, 1.00] | 0.050 | |
| Higher education |  |  |  | -0.69 [-1.12, -0.24] | 0.50 [0.33, 0.79] | **0.002** | |
| CVD diagnosis |  |  |  | -0.16 [-0.51, 0.19] | 0.85 [0.60, 1.21] | 0.372 | |
| Rare alcohol intake |  |  |  | -0.86 [-1.46, -0.24] | 0.42 [0.23, 0.78] | **0.005** | |
| Frequent alcohol intake |  |  |  | -0.86 [-1.35, -0.32] | 0.42 [0.26, 0.73] | **0.001** | |
| Sleep less than 7 hours |  |  |  | 0.23 [-0.16, 0.61] | 1.26 [0.85, 1.83] | 0.227 | |
| Sleep more than 9 hours |  |  |  | -0.84 [-3.71, 0.68] | 0.43 [0.02, 1.97] | 0.408 | |
| Diabetes diagnosis |  |  |  | 0.01 [-0.77, 0.67] | 1.01 [0.46, 1.96] | 0.978 | |
| BMI obese |  |  |  | -0.18 [-0.70, 0.31] | 0.84 [0.50, 1.37] | 0.490 | |
| BMI overweight |  |  |  | -0.21 [-0.59, 0.18] | 0.81 [0.56, 1.19] | 0.290 | |
| BMI severe obese |  |  |  | 0.92 [-0.32, 1.88] | 2.51 [0.73, 6.54] | 0.092 | |
| BMI underweight |  |  |  | -12.10 [-127.41, -117.45] | 0.00 [0.00, 0.00] | 0.965 | |
| Age |  |  |  | 0.20 [0.16, 0.24] | 1.22 [1.18, 1.27] | **<0.001** | |

NB: Model one significantly predicted AD incidence, F(17, 69059) = 113.15, p<0.001. It accounted for 5.66% of the total variance (Nagelkerke R^2^).

Model two also significantly predicted AD incidence, F(24, 69059) = 325.25, p<0.001. It accounted for 16.23% of the total variance (Nagelkerke R^2^). Compared to the previous model it was significantly better at predicting the outcome, F(7, 69035) = 212.10, p<0.001.

Supplementary Table 7. Results from the Poisson regression where AD incidence was modelled by IPAQ group.

|  | Model one | | | Model two | | |
| --- | --- | --- | --- | --- | --- | --- |
|  | Estimate | Risk ratio | p | Estimate | Risk ratio | p |
| IPAQ high | -0.15 [-1.12, 0.91] | 0.86 [0.33, 2.49] | 0.761 | -0.31 [-0.75, 0.13] | 0.73 [0.47, 1.14] | 0.172 |
| IPAQ moderate | -0.94 [-2.11, 0.22] | 0.39 [0.12, 1.24] | 0.102 | -0.45 [-0.90, 0.00] | 0.64 [0.41, 1.00] | 0.050 |
| Sex | 0.63 [-0.36, 1.71] | 1.88 [0.70, 5.51] | 0.223 | 0.29 [-0.07, 0.64] | 1.33 [0.94, 1.90] | 0.111 |
| APOE4 heterozygous | 1.29 [0.19, 2.42] | 3.63 [1.20, 11.27] | **0.021** | 1.42 [1.06, 1.78] | 4.15 [2.90, 5.96] | **<0.001** |
| APOE4 homozygous | 1.78 [-1.15, 3.55] | 5.96 [0.32, 34.86] | 0.099 | 2.67 [2.17, 3.18] | 14.51 [8.73, 24.12] | **<0.001** |
| IPAQ high*Sex | -0.45 [-1.79, 0.84] | 0.64 [0.17, 2.31] | 0.494 |  |  |  |
| IPAQ moderate*Sex | 0.06 [-1.41, 1.52] | 1.06 [0.24 4.58], | 0.935 |  |  |  |
| IPAQ high * APOE4 heterozygous | -0.21 [-1.61, 1.17] | 0.81 [0.20, 3.23] | 0.768 |  |  |  |
| IPAQ moderate * APOE4 heterozygous | 0.45 [-1.03, 1.96] | 1.56 [0.36, 7.07] | 0.553 |  |  |  |
| IPAQ high * APOE4 homozygous | 0.62 [-1.56, 3.71] | 1.86 [0.21, 40.95] | 0.613 |  |  |  |
| IPAQ moderate * APOE4 homozygous | 1.54 [-0.64, 4.65] | 4.66 [0.53, 104.39] | 0.214 |  |  |  |
| Sex * APOE4 heterozygous | -0.64 [-2.17, 0.84] | 0.53 [0.11, 2.31] | 0.399 |  |  |  |
| Sex * APOE4 homozygous | -0.63 [-3.99, 2.72] | 0.53 [0.02, 15.17] | 0.675 |  |  |  |
| IPAQ high * Sex * APOE4 heterozygous | 0.95 [-0.91, 2.85] | 2.58 [0.40, 17.24] | 0.319 |  |  |  |
| IPAQ moderate * Sex * APOE4 heterozygous | 0.61 [-1.36, 2.60] | 1.85 [0.26, 13.49] | 0.541 |  |  |  |
| IPAQ high * Sex * APOE4 homozygous | 0.93 [-2.68, 4.56] | 2.54 [0.07, 95.78] | 0.581 |  |  |  |
| IPAQ moderate * Sex * APOE4 homozygous | -0.04 [-3.72, 3.64] | 0.96 [0.02, 38.26] | 0.984 |  |  |  |
| Bipolar |  |  |  | 1.10 [-0.88, 3.08] | 2.99 [0.41, 21.69] | 0.278 |
| Recurrent depression |  |  |  | -0.60 [-1.75, 0.54] | 0.55 [0.17, 1.72] | 0.302 |
| Ethnicity Asian |  |  |  | 1.16 [-0.02, 2.33] | 3.18 [0.98, 10.28] | 0.054 |
| Ethnicity Black |  |  |  | 0.63 [-1.35, 2.61] | 1.88 [0.26, 13.66] | 0.532 |
| Ethnicity Mixed |  |  |  | 0.26 [-1.71, 2.23] | 1.29 [0.18, 9.30] | 0.798 |
| Has smoked |  |  |  | 0.07 [-0.29, 0.43] | 1.07 [0.75, 1.53] | 0.711 |
| Secondary education |  |  |  | -0.53 [-1.05, 0.00] | 0.59 [0.35, 1.00] | 0.050 |
| Higher education |  |  |  | -0.69 [-1.12, -0.26] | 0.50 [0.33, 0.77] | **0.002** |
| CVD diagnosis |  |  |  | -0.16 [-0.50, 0.19] | 0.86 [0.60, 1.21] | 0.382 |
| Rare alcohol intake |  |  |  | -0.85 [-1.44, -0.25] | 0.43 [0.24, 0.78] | **0.005** |
| Frequent alcohol intake |  |  |  | -0.85 [-1.36, -0.34] | 0.43 [0.26, 0.71] | **0.001** |
| Sleep less than 7 hours |  |  |  | 0.23 [-0.15, 0.61] | 1.25 [0.86, 1.84] | 0.229 |
| Sleep more than 9 hours |  |  |  | -0.83 [-2.81, 1.14] | 0.43 [0.06, 3.13] | 0.408 |
| Diabetes diagnosis |  |  |  | 0.02 [-0.69, 0.72] | 1.02 [0.50, 2.06] | 0.963 |
| BMI obese |  |  |  | -0.17 [-0.67, 0.32] | 0.84 [0.51, 1.38] | 0.493 |
| BMI overweight |  |  |  | -0.20 [-0.58, 0.18] | 0.82 [0.56, 1.19] | 0.296 |
| BMI severe obese |  |  |  | 0.92 [-0.13, 1.97] | 2.51 [0.88, 7.20] | 0.087 |
| BMI underweight |  |  |  | -12.84 [-810.02, 784.34] | 0.00 [0.00, Inf] | 0.975 |
| Age |  |  |  | 0.20 [0.16, 0.23] | 1.22 [1.17, 1.26] | **<0.001** |

NB: Model one was significantly able to predict AD incidence, F(17, 69153) = 112.70, p<0.001. It accounted for 5.64% of the total variance (Nagelkerke R^2^).

Model two could also significantly predict AD incidence, F(24, 69153) = 321.64, p<0.001. It accounted for 16.06% of the total variance (Nagelkerke R^2^).

Supplementary Table 8. Results from the linear regression where volume of ventricular CSF was modelled by average acceleration.

|  | **Model one** | | | **Model two** | |
| --- | --- | --- | --- | --- | --- |
|  | **Estimate** | **p** | **Estimate** | | **p** |
| Average acceleration | -251.39 [-330.35, -172.43] | **<0.001** | -65.54 [-109.68, -17.40] | | **0.007** |
| Sex | 15689.06 [12471.11, 18907.00] | **<0.001** | 7226.62 [6585.26, 7867.98] | | <**0.001** |
| APOE4 heterozygous | -7.00 [-4623.66, 4609.65] | 0.998 | 355.91 [-373.73, 1085.55] | | 0.339 |
| APOE4 homozygous | -6148.60 [-20039.20, 7742.00] | 0.386 | 722.92 [-1337.00, 2782.83] | | 0.492 |
| Average acceleration*Sex | -216.80 [-328.52, -105.08] | **<0.001** |  | |  |
| Average acceleration*APOE4 heterozygous | 11.56 [-143.64, 166.77] | 0.884 |  | |  |
| Average acceleration* APOE4 homozygous | 177.15 [-287.63, 641.93] | 0.455 |  | |  |
| Sex * APOE4 heterozygous | -3591.48 [-10203.02, 3020.06] | 0.287 |  | |  |
| Sex * APOE4 homozygous | 10723.22 [-8977.20, 30423.63] | 0.286 |  | |  |
| Average acceleration * Sex * APOE4 heterozygous | 87.59 [-139.58, 314.76] | 0.450 |  | |  |
| Average acceleration * Sex * APOE4 homozygous | -255.67 [-922.32, 410.99] | 0.452 |  | |  |
| Bipolar |  |  | -722.85 [-6725.08, 5279.37] | | 0.813 |
| Recurrent depression |  |  | 14.42 [-1331.21, 1360.06] | | 0.943 |
| Ethnicity Asian |  |  | -1642.87 [-4734.29, 1448.54] | | 0.298 |
| Ethnicity Black |  |  | -7043.42 [-11660.92, -2425.92] | | **0.003** |
| Ethnicity Mixed |  |  | 406.54 [-2818.02, 3631.10] | | 0.805 |
| Has smoked |  |  | 431.25 [-202.98, 1065.49] | | 0.183 |
| Secondary education |  |  | 1303.58 [-444.40, 3051.55] | | 0.144 |
| Higher education |  |  | 2624.48 [1014.27, 4234.68] | | **0.001** |
| CVD diagnosis |  |  | 1294.53 [621.52, 1967.54] | | **<0.001** |
| Rare alcohol intake |  |  | -1316.05 [-2719.07, 86.97] | | 0.055 |
| Frequent alcohol intake |  |  | -8.12 [-1317.39, 1301.15] | | 0.990 |
| Sleep less than 7 hours |  |  | -50.45 [-778.08, 677.17] | | 0.892 |
| Sleep more than 9 hours |  |  | 1695.03 [-1033.11, 4423.18] | | 0.223 |
| Diabetes diagnosis |  |  | 3520.84 [2079.88, 4961.81] | | **<0.001** |
| BMI obese |  |  | -115.89 [-1061.38, 829.60] | | 0.810 |
| BMI overweight |  |  | -636.28 [-1329.59, 57.02] | | 0.072 |
| BMI severe obese |  |  | 2529.45 [-717.36, 5776.26] | | 0.127 |
| BMI underweight |  |  | -548.99 [-4083.64, 2985.66] | | 0.761 |
| Age |  |  | 1132.81 [1089.05, 1176.57] | | **<0.001** |

NB: Model one significantly predicted volume of ventricular CSF, F(11, 13084) = 93.28, p<0.001. It accounted for 7.19% of the total variance (R^2^).

Model two also significantly predicted the volume of ventricular CSF, F(23, 13072) = 187.5, p<0.001. It accounted for 24.67% of the total variance (R^2^), with an R^2^ change value of 17.48% compared to the previous model.

Supplementary Table 9. Results from the linear regression where volume of ventricular CSF was modelled by IPAQ group.

|  | Model one | | Model two | |
| --- | --- | --- | --- | --- |
|  | Estimate | p | Estimate | p |
| IPAQ high | 1581.75 [12.12, 3151.38] | **0.048** | 822.57 [-51.14, 1696.27] | 0.065 |
| IPAQ moderate | 977.60 [-553.57, 2508.77] | 0.211 | 699.88 [-161.87, 1561.63] | 0.111 |
| Sex | 9402.08 [7554.95, 11249.20] | **<0.001** | 7260.89 [6619.28, 7902.49] | **<0.001** |
| APOE4 heterozygous | -555.98 [-3187.54, 2075.57] | 0.679 | 331.35 [-398.42, 1061.12] | 0.373 |
| APOE4 homozygous | -2253.34 [-9879.73, 5373.05] | 0.562 | 687.50 [-1372.73, 2747.74] | 0.513 |
| IPAQ high*Sex | 307.63 [-1931.98, 2547.23] | 0.788 |  |  |
| IPAQ moderate*Sex | 1222.18 [-987.97, 3431.44] | 0.278 |  |  |
| IPAQ high * APOE4 heterozygous | 1364.53 [-1800.56, 4529.62] | 0.398 |  |  |
| IPAQ moderate * APOE4 heterozygous | 434.99 [-2687.61, 3557.59] | 0.785 |  |  |
| IPAQ high * APOE4 homozygous | 1811.00 [-7373.52, 10995.51] | 0.699 |  |  |
| IPAQ moderate * APOE4 homozygous | 913.98 [-7982.82, 9810.78] | 0.840 |  |  |
| Sex * APOE4 heterozygous | -177.20 [-4068.32, 3713.93] | 0.929 |  |  |
| Sex * APOE4 homozygous | 6094.24 [-4599.15, 16787.64] | 0.264 |  |  |
| IPAQ high * Sex * APOE4 heterozygous | -974.77 [-5639.27, 3689.74] | 0.682 |  |  |
| IPAQ moderate * Sex * APOE4 heterozygous | -1525.72 [-6165.04, 3113.60] | 0.519 |  |  |
| IPAQ high * Sex * APOE4 homozygous | -1914.31 [-14752.80, 10924.18] | 0.770 |  |  |
| IPAQ moderate * Sex * APOE4 homozygous | -6863.40 [-19995.65, 6268.84] | 0.306 |  |  |
| Bipolar |  |  | -791.25 [-6794.51, 5212.01] | 0.796 |
| Recurrent depression |  |  | 73.36 [-1271.33, 1418.84] | 0.914 |
| Ethnicity Asian |  |  | -1680.64 [-4773.01, 1411.73] | 0.287 |
| Ethnicity Black |  |  | -7069.07 [-11687.63, -2450.51] | **0.003** |
| Ethnicity Mixed |  |  | 391.23 [-2834.10, 3616.55] | 0.812 |
| Has smoked |  |  | 431.08 [-203.30, 1065.46] | 0.183 |
| Secondary education |  |  | 1371.74 [-376.96, 3120.43] | 0.124 |
| Higher education |  |  | 2674.27 [1062.79, 4285.75] | **0.001** |
| CVD diagnosis |  |  | 1342.04 [669.35, 2014.73] | **<0.001** |
| Rare alcohol intake |  |  | -1286.45 [-2689.75, 116.84] | 0.072 |
| Frequent alcohol intake |  |  | -50.23 [-1359.58, 1259.12] | 0.940 |
| Sleep less than 7 hours |  |  | -57.54 [-785.56, 670.47] | 0.877 |
| Sleep more than 9 hours |  |  | 1865.39 [-862.22, 4593.00] | 0.180 |
| Diabetes diagnosis |  |  | 3702.00 [2261.98, 5142.01] | **<0.001** |
| BMI obese |  |  | 203.74 [-725.57, 1133.05] | 0.667 |
| BMI overweight |  |  | -490.44 [-1178.88, 197.99] | 0.163 |
| BMI severe obese |  |  | 3186.59 [-41.96, 6415.15] | **0.**053 |
| BMI underweight |  |  | -628.23 [-4162.92, 2906.46] | 0.728 |
| Age |  |  | 1143.44 [1100.61, 1186.26] | **<0.001** |

NB: Model one significant predicted volume of ventricular CSF, F(17, 13078) = 48.19, p<0.001. It accounted for 5.77% of the total variance (R^2^).

Model two also significantly predicted the volume of ventricular CSF, F(24, 13071) = 179.4, p<0.001. It accounted for 24.64% of the total variance (R^2^), with an R^2^ value of 18.87% compared to the previous model.

Supplementary Table 10. Results from the linear regression where total brain volume was modelled by average acceleration.

|  | Model one | | | Model two | |
| --- | --- | --- | --- | --- | --- |
|  | Estimate | p | Estimate | | p |
| Average acceleration | 630.61 [254.66, 1006.56] | .**<0.001** | -124.11 [-350.83, 102.60] | | 0.283 |
| Sex | 100068.15 [84746.08, 115390.22] | **<0.001** | 123277.97 [120126.48, 126429.46] | | **<0.001** |
| APOE4 heterozygous | -25619.38 [-47601.33, -3637.43] | **0.022** | 1593.20 [-1992.07, 5178.46] | | 0.384 |
| APOE4 homozygous | -3676.64 [-69815.94, 62462.67] | 0.913 | -527.74 [-10649.64, 9594.17] | | 0.919 |
| Average acceleration*Sex | 496.46 [-35.48, 1028.39] | 0.067 |  | |  |
| Average acceleration*APOE4 heterozygous | 873.23 [134.23, 1612.23] | **0.021** |  | |  |
| Average acceleration* APOE4 homozygous | 219.19 [-1993.84, 2432.22] | 0.846 |  | |  |
| Sex * APOE4 heterozygous | 43435.51 [11955.04, 74915.99] | **0.007** |  | |  |
| Sex * APOE4 homozygous | 48604.75 [-45197.69, 142407.19] | 0.310 |  | |  |
| Average acceleration * Sex * APOE4 heterozygous | -1213.21 [-2294.86, -131.55] | **0.028** |  | |  |
| Average acceleration * Sex * APOE4 homozygous | -1846.23 [-5020.45, 1328.00] | 0.254 |  | |  |
| Bipolar |  |  | -4939.51 [-34432.91, 24553.89] | | 0.743 |
| Recurrent depression |  |  | 6708.33 [96.21, 13320.44] | | **0.047** |
| Ethnicity Asian |  |  | -96708.04 [-111898.47, -81517.61] | | **<0.001** |
| Ethnicity Black |  |  | -57720.53 [-80409.76, -35031.31] | | **<0.001** |
| Ethnicity Mixed |  |  | -44347.94 [-60192.63, -28503.26] | | **<0.001** |
| Has smoked |  |  | -1665.42 [-4781.88, 1451.03] | | 0.295 |
| Secondary education |  |  | 10692.14 [2103.04, 19281.24] | | **0.015** |
| Higher education |  |  | 20278.79 [12366.65, 28190.93] | | **<0.001** |
| CVD diagnosis |  |  | -2965.74 [-6272.74, 341.26] | | 0.079 |
| Rare alcohol intake |  |  | 6883.35 [-10.72, 13777.43] | | 0.050 |
| Frequent alcohol intake |  |  | 4710.49 [-1722.93, 11143.91] | | 0.151 |
| Sleep less than 7 hours |  |  | -4732.45 [-8307.82, -1157.07] | | **0.009** |
| Sleep more than 9 hours |  |  | -10959.32 [-24364.72, 2446.08] | | 0.109 |
| Diabetes diagnosis |  |  | -22689.92 [-29770.47, -15609.37] | | **<0.001** |
| BMI obese |  |  | 535.80 [-4110.10, 5181.71] | | 0.821 |
| BMI overweight |  |  | 2193.52 [-1213.20, 5600.24] | | 0.207 |
| BMI severe obese |  |  | 17105.72 [1151.72, 33059.72] | | **0.036** |
| BMI underweight |  |  | -16496.54 [-33864.90, 871.83] | | 0.063 |
| Age |  |  | -4254.93 [-4469.96, -4039.90] | | **<0.001** |

NB: Model one significantly predicted total brain volume, F(11, 13084) = 448.20, p<0.001. It accounted for 27.31% of the total variance (R^2^).

Model two significantly predicted total brain volume, F(23, 13072) = 337.70, p<0.001. It accounted for 37.16% of the total variance (R^2^), with an R^2^ change value of 9.85%.

Supplementary Table 11. Results from the linear regression where total brain volume was modelled by IPAQ group.

|  | Model one | | Model two | |  |
| --- | --- | --- | --- | --- | --- |
|  | Estimate | p | Estimate | p | |
| IPAQ high | -7924.94 [-15359.31, -490.56] | **0.037** | 2996.97 [-1295.11, 7289.05] | 0.171 | |
| IPAQ moderate | 1413.93 [-5838.30, 8666.17] | 0.702 | 4398.46 [165.11, 8631.82] | **0.042** | |
| Sex | 107933.52 [99184.82, 116682.22] | **<0.001** | 123427.54 [120275.66, 126579.42] | **<0.001** | |
| APOE4 heterozygous | -327.74 [-12791.80, 12136.31] | 0.959 | 1553.03 [-2031.97, 5138.02] | 0.396 | |
| APOE4 homozygous | -8931.16 [-45052.70, 27190.38] | 0.628 | -531.53 [-10652.42, 9589.35] | 0.918 | |
| IPAQ high*Sex | 11430.15 [822.53, 22037.77] | **0.035** |  |  | |
| IPAQ moderate*Sex | 1730.41 [-8733.47, 12194.29] | 0.746 |  |  | |
| IPAQ high * APOE4 heterozygous | 7309.79 [-7681.29, 22300.88] | 0.339 |  |  | |
| IPAQ moderate * APOE4 heterozygous | -5376.69 [-20166.54, 9413.15] | 0.476 |  |  | |
| IPAQ high * APOE4 homozygous | 19144.34 [-24357.06, 62645.74] | 0.388 |  |  | |
| IPAQ moderate * APOE4 homozygous | 10587.70 [-31550.99, 52726.40] | 0.622 |  |  | |
| Sex * APOE4 heterozygous | 6857.22 [-11572.64, 25287.09] | 0.466 |  |  | |
| Sex * APOE4 homozygous | 33756.37 [-16891.68, 84404.42] | 0.191 |  |  | |
| IPAQ high * Sex * APOE4 heterozygous | -8717.91 [-30810.81, 13374.98] | 0.439 |  |  | |
| IPAQ moderate * Sex * APOE4 heterozygous | 12001.34 [-9972.28, 33974.96] | 0.284 |  |  | |
| IPAQ high * Sex * APOE4 homozygous | -51739.66 [-112547.72, 9068.40] | 0.095 |  |  | |
| IPAQ moderate * Sex * APOE4 homozygous | -40309.37 [-102508.76, 21890.02] | 0.204 |  |  | |
| Bipolar |  |  | -5082.67 [-34573.61, 24408.26] | 0.736 | |
| Recurrent depression |  |  | 6767.83 [160.10, 13375.56] | **0.045** | |
| Ethnicity Asian |  |  | -96943.43 [-112134.66, -81752.19] | **<0.001** | |
| Ethnicity Black |  |  | -57681.49 [-80370.12, -34992.85] | **<0.001** | |
| Ethnicity Mixed |  |  | -44362.15 [-60206.53, -28517.78] | **<0.001** | |
| Has smoked |  |  | -1637.66 [-4754.05, 1478.73] | 0.303 | |
| Secondary education |  |  | 10729.99 [2139.53, 19320.45] | **0.014** | |
| Higher education |  |  | 20196.33 [12279.96, 28112.69] | **<0.001** | |
| CVD diagnosis |  |  | -2860.22 [-6164.79, 444.35] | 0.090 | |
| Rare alcohol intake |  |  | 6942.78 [49.11, 13836.44] | **0.048** | |
| Frequent alcohol intake |  |  | 4563.32 [-1868.85, 10995.49] | 0.164 | |
| Sleep less than 7 hours |  |  | -4680.43 [-8256.79, -1104.06] | **0.010** | |
| Sleep more than 9 hours |  |  | -10469.01 [-23868.36, 2930.34] | 0.126 | |
| Diabetes diagnosis |  |  | -22189.09 [-29263.16, -15115.02] | **<0.001** | |
| BMI obese |  |  | 1259.42 [-3305.80, 5824.64] | 0.589 | |
| BMI overweight |  |  | 2529.48 [-852.45, 5911.42] | 0.143 | |
| BMI severe obese |  |  | 18740.40 [2880.14, 34600.65] | **0.021** | |
| BMI underweight |  |  | -16526.76 [-33890.89, 837.38] | 0.062 | |
| Age |  |  | -4237.94 [-4448.33, -4027.55] | **<0.001** | |

NB: Model one significantly predicted total brain volume, F(17, 13078) = 285.40, p<0.001. It accounted for 26.97% of the total variance (R^2^).

Model two also significantly predicted total brain volume, F(24, 13071) = 323.80, p<0.001. It accounted for 37.17% of the total variance (R^2^) with an R^2^ change value of 10.20%.

Supplementary Table 12. Results from the linear regression where % correct on the SDST was modelled by average acceleration.

|  | Model one | | Model two | | |
| --- | --- | --- | --- | --- | --- |
|  | Estimate | p | Estimate | p |  |
| Average acceleration | 0.00 [-0.04, 0.04] | 0.911 | -0.01 [-0.04, 0.01] | 0.296 |  |
| Sex | -0.61 [-2.15, 0.94] | 0.441 | 0.58 [0.25, 0.91] | **0.001** |  |
| APOE4 heterozygous | -1.03 [-3.27, 1.21] | 0.367 | -0.22 [-0.59, 0.16] | 0.264 |  |
| APOE4 homozygous | -6.11 [-12.44, 0.22] | 0.058 | -0.91 [-2.00, 0.17] | 0.099 |  |
| Average acceleration*Sex | 0.04 [-0.01, 0.09] | 0.160 |  |  |  |
| Average acceleration*APOE4 heterozygous | 0.04 [-0.04, 0.11] | 0.335 |  |  |  |
| Average acceleration* APOE4 homozygous | 0.19 [-0.02, 0.40] | 0.073 |  |  |  |
| Sex * APOE4 heterozygous | 0.40 [-2.79, 3.60] | 0.804 |  |  |  |
| Sex * APOE4 homozygous | 6.02 [-2.91, 14.96] | 0.186 |  |  |  |
| Average acceleration * Sex * APOE4 heterozygous | -0.03 [0.14, 0.08] | 0.618 |  |  |  |
| Average acceleration * Sex * APOE4 homozygous | -0.23 [-0.53, 0.07] | 0.138 |  |  |  |
| Bipolar |  |  | -0.94 [-4.01, 2.14] | 0.550 |  |
| Recurrent depression |  |  | -0.44 [-1.14, 0.26] | 0.221 |  |
| Ethnicity Asian |  |  | -0.56 [-2.17, 1.06] | 0.499 |  |
| Ethnicity Black |  |  | -4.23 [-6.86, -1.59] | **0.002** |  |
| Ethnicity Mixed |  |  | -1.65 [-3.31, 0.00] | 0.051 |  |
| Has smoked |  |  | 0.07 [-0.25, 0.40] | 0.655 |  |
| Secondary education |  |  | 1.54 [0.49, 2.60] | **0.004** |  |
| Higher education |  |  | 1.80 [0.81, 2.79] | **<0.001** |  |
| CVD diagnosis |  |  | -0.25 [-0.60, 0.10] | 0.159 |  |
| Rare alcohol intake |  |  | -0.38 [-1.11, 0.36] | 0.318 |  |
| Frequent alcohol intake |  |  | -0.21 [-0.90, 0.48] | 0.550 |  |
| Sleep less than 7 hours |  |  | -0.29 [-0.67, 0.09] | 0.129 |  |
| Sleep more than 9 hours |  |  | 0.04 [-1.40, 1.49] | 0.952 |  |
| Diabetes diagnosis |  |  | -0.87 [-1.62, -0.11] | **0.024** |  |
| BMI obese |  |  | -0.25 [-0.74, 0.24] | 0.318 |  |
| BMI overweight |  |  | -0.21 [-0.57, 0.15] | 0.255 |  |
| BMI severe obese |  |  | -1.35 [-2.95, 0.15] | 0.099 |  |
| BMI underweight |  |  | -0.39 [-2.11, 1.33] | 0.654 |  |
| Age |  |  | -0.14 [-0.16, -0.11] | **<0.001** |  |

NB: The first model was unable to significantly predict % correct scores on the SDST, F(11, 10095) = 1.77, p>0.05.

The full model was able to significantly predict % correct scores on the SDST, F(23, 10083) = 9.61, p<0.001. It was able to account for 1.81% of the total variance (R^2^).

Supplementary Table 13. Results from the linear regression (Yeo-Johnson transformation) where % correct on the SDST was modelled by average acceleration.

|  | Model one | | Model two | |
| --- | --- | --- | --- | --- |
|  | Estimate | p | Estimate | p |
| Average acceleration | 0.00 [0.00, 0.00] | 0.926 | 0.00 [0.00, 0.00] | 0.250 |
| Sex | -0.01 [-0.04, 0.02] | 0.642 | 0.01 [0.00, 0.01] | **0.033** |
| APOE4 heterozygous | -0.02 [-0.06, 0.03] | 0.400 | 0.00 [-0.01, 0.00] | 0.212 |
| APOE4 homozygous | -0.09 [-0.21, 0.04] | 0.179 | -0.01 [-0.03, 0.01] | 0.510 |
| Average acceleration*Sex | 0.00 [0.00, 0.00] | 0.369 |  |  |
| Average acceleration*APOE4 heterozygous | 0.00 [0.00, 0.00] | 0.377 |  |  |
| Average acceleration* APOE4 homozygous | 0.00 [0.00, 0.01] | 0.188 |  |  |
| Sex * APOE4 heterozygous | 0.01 [-0.05, 0.07] | 0.784 |  |  |
| Sex * APOE4 homozygous | 0.09 [-0.09, 0.27] | 0.324 |  |  |
| Average acceleration * Sex * APOE4 heterozygous | 0.00 [0.00, 0.00] | 0.550 |  |  |
| Average acceleration * Sex * APOE4 homozygous | 0.00 [-0.01, 0.00] | 0.287 |  |  |
| Bipolar |  |  | -0.02 [-0.08, 0.04] | 0.513 |
| Recurrent depression |  |  | 0.00 [-0.02, 0.01] | 0.770 |
| Ethnicity Asian |  |  | -0.01 [-0.04, 0.03] | 0.760 |
| Ethnicity Black |  |  | -0.14 [-0.19, -0.09] | **<0.001** |
| Ethnicity Mixed |  |  | -0.02 [-0.05, 0.01] | 0.217 |
| Has smoked |  |  | 0.00 [-0.01, 0.01] | 0.818 |
| Secondary education |  |  | 0.02 [0.00, 0.04] | 0.069 |
| Higher education |  |  | 0.02 [0.00, 0.04] | **0.039** |
| CVD diagnosis |  |  | 0.00 [-0.01, 0.00] | 0.543 |
| Rare alcohol intake |  |  | -0.01 [-0.02, 0.01] | 0.460 |
| Frequent alcohol intake |  |  | 0.00 [-0.02, 0.01] | 0.850 |
| Sleep less than 7 hours |  |  | 0.00 [-0.01, 0.00] | 0.472 |
| Sleep more than 9 hours |  |  | 0.00 [-0.02, 0.03] | 0.757 |
| Diabetes diagnosis |  |  | -0.02 [-0.03, 0.00] | **0.025** |
| BMI obese |  |  | 0.00 [-0.01, 0.01] | 0.402 |
| BMI overweight |  |  | 0.00 [-0.01, 0.00] | 0.426 |
| BMI severe obese |  |  | -0.01 [-0.04, 0.02] | 0.491 |
| BMI underweight |  |  | -0.01 [-0.04, 0.03] | 0.642 |
| Age |  |  | 0.00 [0.00, 0.00] | **<0.001** |

NB: Model one was not significantly able to predict % correct scores on the SDST, F(11, 10095) = 0.83, p>0.05.

Model two was significantly able to predict % correct scores on the SDST, F(23, 10083) = 5.76, p<0.001. It accounted for 1.07% of the total variance (R^2^).

Supplementary Table 14. Results from the linear regression where % correct on the SDST was modelled by IPAQ group.

|  | Model one | | Model two | | |  |
| --- | --- | --- | --- | --- | --- | --- |
|  | Estimate | p | | Estimate | p | |
| IPAQ high | -0.74 [-1.48, 0.00] | **0.049** | | -0.14 [-0.59, 0.31] | 0.553 | |
| IPAQ moderate | 0.12 [-0.59, 0.84] | 0.736 | | 0.18 [-0.26, 0.62] | 0.420 | |
| Sex | 0.24 [-0.61, 1.08] | 0.586 | | 0.59 [0.26, 0.93] | **0.001** | |
| APOE4 heterozygous | -0.81 [-2.05, 0.43] | 0.199 | | -0.21 [-0.59, 0.16] | 0.266 | |
| APOE4 homozygous | 0.31 [-3.17, 3.78] | 0.863 | | -0.91 [-1.99, 0.18] | 0.101 | |
| IPAQ high*Sex | 0.46 [-0.58, 1.50] | 0.384 | |  |  | |
| IPAQ moderate*Sex | 0.13 [-0.89, 1.15] | 0.803 | |  |  | |
| IPAQ high * APOE4 heterozygous | 1.82 [0.32, 3.32] | **0.017** | |  |  | |
| IPAQ moderate * APOE4 heterozygous | 0.40 [-1.07, 1.86] | 0.593 | |  |  | |
| IPAQ high * APOE4 homozygous | 0.32 [-3.96, 4.60] | 0.883 | |  |  | |
| IPAQ moderate * APOE4 homozygous | -2.06 [-6.23, 2.11] | 0.332 | |  |  | |
| Sex * APOE4 heterozygous | -0.21 [-1.98, 1.57] | 0.820 | |  |  | |
| Sex * APOE4 homozygous | -0.37 [-5.02, 4.29] | 0.877 | |  |  | |
| IPAQ high * Sex * APOE4 heterozygous | -0.29 [-2.44, 1.86] | 0.792 | |  |  | |
| IPAQ moderate * Sex * APOE4 heterozygous | -0.31 [-2.44, 1.81] | 0.775 | |  |  | |
| IPAQ high * Sex * APOE4 homozygous | -3.84 [-9.70, 2.02] | 0.199 | |  |  | |
| IPAQ moderate * Sex * APOE4 homozygous | 2.80 [-3.01, 8.61] | 0.344 | |  |  | |
| Bipolar |  |  | | -0.95 [-4.02, 2.12] | 0.544 | |
| Recurrent depression |  |  | | -0.44 [-1.14, 0.27] | 0.223 | |
| Ethnicity Asian |  |  | | -0.56 [-2.17, 1.06] | 0.499 | |
| Ethnicity Black |  |  | | -4.20 [-6.84, -1.56] | **0.002** | |
| Ethnicity Mixed |  |  | | -1.61 [-3.26, 0.05] | 0.057 | |
| Has smoked |  |  | | 0.08 [-0.25, 0.41] | 0.651 | |
| Secondary education |  |  | | 1.54 [0.49, 2.60] | **0.004** | |
| Higher education |  |  | | 1.79 [0.80, 2.78] | **<0.001** | |
| CVD diagnosis |  |  | | -0.24 [-0.59, 0.10] | 0.169 | |
| Rare alcohol intake |  |  | | -0.37 [-1.11, 0.37] | 0.326 | |
| Frequent alcohol intake |  |  | | -0.22 [-0.90, 0.47] | 0.533 | |
| Sleep less than 7 hours |  |  | | -0.29 [-0.67, 0.09] | 0.135 | |
| Sleep more than 9 hours |  |  | | 0.08 [-1.36, 1.52] | 0.916 | |
| Diabetes diagnosis |  |  | | -0.85 [-1.60, -0.09] | **0.028** | |
| BMI obese |  |  | | -0.21 [-0.70, 0.27] | 0.392 | |
| BMI overweight |  |  | | -0.19 [-0.54, 0.17] | 0.307 | |
| BMI severe obese |  |  | | -1.27 [-2.86, 0.32] | 0.118 | |
| BMI underweight |  |  | | -0.40 [-2.12, 1.32] | 0.646 | |
| Age |  |  | | -0.13 [-0.16, -0.11] | **<0.001** | |

NB: Model one was significantly able to predict % correct scores on the SDST, F(17, 10089) = 2.14, p=0.004. It accounted for 0.19% of the total variance (R^2^).

Model two was also significantly able to predict % correct scores on the SDST, F(24, 10082)=9.29, p<0.001. It accounted for 1.93% of the total variance (R^2^) with an R^2^ change value of 1.74% compared to the previous model.

Supplementary Table 15. Results from the linear regression (Yeo-Johnson transformation) where % correct on the SDST was modelled by IPAQ group.

|  | Model one | | Model two | | |
| --- | --- | --- | --- | --- | --- |
|  | Estimate | p | Estimate | p |  |
| IPAQ high | -0.01 [-0.03, 0.00] | 0.138 | 0.00 [-0.01, 0.01] | 0.984 |  |
| IPAQ moderate | 0.01 [-0.01, 0.02] | 0.450 | 0.01 [0.00, 0.02] | 0.133 |  |
| Sex | 0.00 [-0.01, 0.02] | 0.583 | 0.01 [0.00, 0.01] | **0.025** |  |
| APOE4 heterozygous | -0.02 [-0.05, 0.00] | 0.094 | 0.00 [-0.01, 0.00] | 0.212 |  |
| APOE4 homozygous | 0.01 [-0.06, 0.08] | 0.784 | -0.01 [-0.03, 0.01] | 0.525 |  |
| IPAQ high * Sex | 0.01 [-0.02, 0.03] | 0.616 |  |  |  |
| IPAQ moderate *Sex | 0.00 [-0.02, 0.02] | 0.978 |  |  |  |
| IPAQ high * APOE4 heterozygous | 0.04 [0.01, 0.07] | **0.014** |  |  |  |
| IPAQ moderate * APOE4 heterozygous | 0.02 [-0.01, 0.05] | 0.258 |  |  |  |
| IPAQ high * APOE4 homozygous | 0.01 [-0.08, 0.09] | 0.905 |  |  |  |
| IPAQ moderate * APOE4 homozygous | -0.04 [-0.12, 0.05] | 0.391 |  |  |  |
| Sex* APOE4 heterozygous | 0.00 [-0.04, 0.03] | 0.824 |  |  |  |
| Sex* APOE4 homozygous | -0.01 [-0.10, 0.09] | 0.889 |  |  |  |
| IPAQ high * sex * APOE4 heterozygous | 0.00 [-0.04, 0.04] | 0.952 |  |  |  |
| IPAQ moderate * sex * APOE4 heterozygous | -0.01 [-0.06, 0.03] | 0.509 |  |  |  |
| IPAQ high * sex * APOE4 homozygous | -0.05 [-0.16, 0.07] | 0.444 |  |  |  |
| IPAQ moderate * sex * APOE4 homozygous | 0.04 [-0.07, 0.16] | 0.478 |  |  |  |
| Bipolar |  |  | -0.02 [-0.08, 0.04] | 0.504 |  |
| Recurrent depression |  |  | 0.00 [-0.02, 0.01] | 0.765 |  |
| Ethnicity Asian |  |  | -0.01 [-0.04, 0.03] | 0.752 |  |
| Ethnicity Black |  |  | -0.14 [-0.19, -0.09] | **<0.001** |  |
| Ethnicity Mixed |  |  | -0.02 [-0.05, 0.01] | 0.239 |  |
| Has smoked |  |  | 0.00 [-0.01, 0.01] | 0.806 |  |
| Secondary education |  |  | 0.02 [0.00, 0.04] | 0.069 |  |
| Higher education |  |  | 0.02 [0.00, 0.04] | **0.042** |  |
| CVD diagnosis |  |  | 0.00 [-0.01, 0.00] | 0.570 |  |
| Rare alcohol intake |  |  | -0.01 [-0.02, 0.01] | 0.471 |  |
| Frequent alcohol intake |  |  | 0.00 [-0.02, 0.01] | 0.824 |  |
| Sleep less than 7 hours |  |  | 0.00 [-0.01, 0.00] | 0.494 |  |
| Sleep more than 9 hours |  |  | 0.01 [-0.02, 0.03] | 0.710 |  |
| Diabetes diagnosis |  |  | -0.02 [-0.03, 0.00] | **0.031** |  |
| BMI obese |  |  | 0.00 [-0.01, 0.01] | 0.534 |  |
| BMI overweight |  |  | 0.00 [-0.01, 0.00] | 0.522 |  |
| BMI severe obese |  |  | -0.01[-0.04, 0.02] | 0.582 |  |
| BMI underweight |  |  | -0.01 [-0.04, 0.03] | 0.638 |  |
| Age |  |  | 0.00 [0.00, 0.00] | **<0.001** |  |

NB: Model one was significantly able to predict % correct scores on the SDST, F(17, 10089) = 1.78, p=0.025. It accounted for 0.13% of the total variance (R^2^).

Model two was significantly able to predict % correct scores on the SDST, F(24, 10082) = 5.64, p=0,.001. It accounted for 1.09% of the total variance R^2^, with an R^2^ change value of 0.96% compared to the previous model.

Supplementary Table 16. Results from the linear regression where duration to complete the TMT-B was modelled by average acceleration.

|  | Model one | | | Model two | |
| --- | --- | --- | --- | --- | --- |
|  | Estimate | p | Estimate | | p |
| Average acceleration | -0.05 [-0.06, -0.03] | **<0.001** | 0.01 [0.00, 0.02] | | 0.062 |
| Sex | 0.11 [-0.54, 0.77] | 0.742 | -0.04 [-0.17, 0.09] | | 0.584 |
| APOE4 heterozygous | -0.59 [-1.54, 0.36] | 0.223 | 0.08 [-0.07, 0.23] | | 0.282 |
| APOE4 homozygous | -0.12 [-2.80, 2.57] | 0.931 | 0.04 [-0.38, 0.46] | | 0.851 |
| Average acceleration*Sex | 0.00 [-0.02, 0.03] | 0.713 |  | |  |
| Average acceleration*APOE4 heterozygous | 0.02 [-0.01, 0.05] | 0.159 |  | |  |
| Average acceleration* APOE4 homozygous | 0.00 [-0.09, 0.09] | 0.952 |  | |  |
| Sex * APOE4 heterozygous | 0.10 [-1.25, 1.46] | 0.882 |  | |  |
| Sex * APOE4 homozygous | -0.43 [-4.22, 3.37] | 0.825 |  | |  |
| Average acceleration * Sex * APOE4 heterozygous | -0.01 [-0.05, 0.04] | 0.733 |  | |  |
| Average acceleration * Sex * APOE4 homozygous | 0.02 [-0.11, 0.15] | 0.780 |  | |  |
| Bipolar |  |  | -0.51 [-1.71, 0.69] | | 0.406 |
| Recurrent depression |  |  | 0.16 [-0.11, 0.44] | | 0.248 |
| Ethnicity Asian |  |  | 1.53 [0.90, 2.16] | | **<0.001** |
| Ethnicity Black |  |  | 1.80 [0.78, 2.83] | | **0.001** |
| Ethnicity Mixed |  |  | 0.86 [0.22, 1.51] | | **0.009** |
| Has smoked |  |  | 0.11 [-0.01, 0.24] | | 0.079 |
| Secondary education |  |  | -1.28 [-1.69, -0.87] | | **<0.001** |
| Higher education |  |  | -2.05 [-2.43, -1.66] | | **<0.001** |
| CVD diagnosis |  |  | 0.26 [0.12, 0.40] | | **<0.001** |
| Rare alcohol intake |  |  | -0.22 [-0.51, 0.07] | | 0.132 |
| Frequent alcohol intake |  |  | -0.37 [-0.64, -0.11] | | **0.006** |
| Sleep less than 7 hours |  |  | 0.14 [-0.01, 0.28] | | 0.070 |
| Sleep more than 9 hours |  |  | 0.78 [0.22, 1.34] | | **0.007** |
| Diabetes diagnosis |  |  | 0.43 [0.13, 0.72] | | **0.004** |
| BMI obese |  |  | 0.34 [0.15, 0.53] | | **<0.001** |
| BMI overweight |  |  | 0.04 [-0.10, 0.18] | | 0.562 |
| BMI severe obese |  |  | 0.03 [-0.59, 0.65] | | 0.921 |
| BMI underweight |  |  | 0.75 [0.08, 1.42] | | **0.029** |
| Age |  |  | 0.18 [0.17, 0.19] | | **<0.001** |

NB: Model one was significantly able to predict the time taken to complete the TMT-B, F(11, 10095) = 7.41, p<0.001. It accounted for 0.69% of the total variance (R^2^).

Model two was also significantly able to predict the time taken to complete the TMT-B, F(23, 10083) = 99.56, p<0.001. It accounted for 17.17% of the total variance (R^2^), with an R^2^ change value of 17.46% compared to the previous model.

Supplementary Table 17. Results from the linear regression where duration to complete the TMT–B was modelled by IPAQ group.

|  | Model one | | Model two | | |
| --- | --- | --- | --- | --- | --- |
|  | Estimate | p | | Estimate | p |
| IPAQ high | 0.41 [0.09, 0.72] | **0.011** | | 0.49 [0.31, 0.66] | **<0.001** |
| IPAQ moderate | 0.05 [-0.25, 0.35] | 0.746 | | 0.27 [0.10, 0.45] | **0.002** |
| Sex | 0.03 [-0.33, 0.39] | 0.876 | | -0.04 [-0.17, 0.09] | 0.536 |
| APOE4 heterozygous | -0.06 [-0.58, 0.47] | 0.837 | | 0.08 [-0.07, 0.22] | 0.305 |
| APOE4 homozygous | 0.64 [-0.84, 2.12] | 0.393 | | 0.05 [-0.37, 0.47] | 0.819 |
| IPAQ high*Sex | 0.25 [-0.20, 0.69] | 0.275 | |  |  |
| IPAQ moderate*Sex | 0.34 [-0.10, 0.77] | 0.129 | |  |  |
| IPAQ high * APOE4 heterozygous | 0.12 [-0.52, 0.75] | 0.718 | |  |  |
| IPAQ moderate * APOE4 heterozygous | 0.10 [-0.52, 0.73] | 0.744 | |  |  |
| IPAQ high * APOE4 homozygous | -1.17 [-2.98, 0.65] | 0.209 | |  |  |
| IPAQ moderate * APOE4 homozygous | -1.00 [-2.77, 0.78] | 0.270 | |  |  |
| Sex * APOE4 heterozygous | -0.06 [-0.82, 0.69] | 0.873 | |  |  |
| Sex * APOE4 homozygous | -1.39 [-3.37, 0.59] | 0.169 | |  |  |
| IPAQ high * Sex * APOE4 heterozygous | -0.21 [-1.12, 0.71] | 0.660 | |  |  |
| IPAQ moderate * Sex * APOE4 heterozygous | 0.05 [-0.86, 0.95] | 0.919 | |  |  |
| IPAQ high * Sex * APOE4 homozygous | 2.80 [0.31, 5.29] | **0.028** | |  |  |
| IPAQ moderate * Sex * APOE4 homozygous | 1.12 [-1.35, 3.59] | 0.374 | |  |  |
| Bipolar |  |  | | -0.52 [-1.72, 0.68] | 0.395 |
| Recurrent depression |  |  | | 0.15 [-0.13, 0.43] | 0.294 |
| Ethnicity Asian |  |  | | 1.50 [0.87, 2.13] | **<0.001** |
| Ethnicity Black |  |  | | 1.78 [0.75, 2.80] | **0.001** |
| Ethnicity Mixed |  |  | | 0.83 [0.19, 1.48] | **0.011** |
| Has smoked |  |  | | 0.12 [-0.01, 0.25] | 0.070 |
| Secondary education |  |  | | -1.28 [-1.69, -0.87] | **<0.001** |
| Higher education |  |  | | -2.05 [-2.44, -1.67] | **<0.001** |
| CVD diagnosis |  |  | | 0.26 [0.12, 0.39] | **<0.001** |
| Rare alcohol intake |  |  | | -0.23 [-0.51, 0.06] | 0.121 |
| Frequent alcohol intake |  |  | | -0.37 [-0.64, -0.11] | **0.006** |
| Sleep less than 7 hours |  |  | | 0.14 [-0.01, 0.29] | 0.065 |
| Sleep more than 9 hours |  |  | | 0.79 [0.22, 1.35] | **0.006** |
| Diabetes diagnosis |  |  | | 0.45 [0.15, 0.74] | **0.003** |
| BMI obese |  |  | | 0.35 [0.16, 0.54] | **<0.001** |
| BMI overweight |  |  | | 0.04 [-0.10, 0.18] | 0.595 |
| BMI severe obese |  |  | | 0.06 [-0.56, 0.68] | 0.857 |
| BMI underweight |  |  | | 0.78 [0.11, 1.45] | **0.023** |
| Age |  |  | | 0.17 [0.17, 0.18] | **<0.001** |

NB: Model one was significantly able to predict the time taken to complete the TMT-B, F(17, 10089) = 3.49, p<0.001. It accounted for 0.42% of the total variance (R^2^).

Model two was also significantly able to predict the time taken to complete the TMT-B, F(24, 10082) = 93.89, p<0.001. It accounted for 18.07% of the total variance (R^2^), with an R^2^ value of 17.65% compared to the previous model.

Supplementary Table 18. Results from the linear regression where % correct on the numeric memory task was modelled by average acceleration.

|  | Model one | | Model two | | |
| --- | --- | --- | --- | --- | --- |
|  | Estimate | p | | Estimate | p |
| Average acceleration | 0.03 [0.00, 0.05] | 0.089 | | -0.01 [-0.02, 0.01] | 0.536 |
| Sex | 0.40 [-0.78, 1.57] | 0.507 | | 0.58 [0.33, 0.83] | **<0.001** |
| APOE4 heterozygous | 1.17 [-0.54, 2.87] | 0.179 | | 0.06 [-0.23, 0.35] | 0.665 |
| APOE4 homozygous | 2.76 [-2.06, 7.58] | 0.261 | | 0.68 [-0.15, 1.51] | 0.108 |
| Average acceleration*Sex | 0.00 [-0.04, 0.04] | 0.870 | |  |  |
| Average acceleration*APOE4 heterozygous | -0.04 [-0.10, 0.02] | 0.163 | |  |  |
| Average acceleration* APOE4 homozygous | -0.08 [-0.24, 0.08] | 0.315 | |  |  |
| Sex * APOE4 heterozygous | 0.32 [-2.12, 2.75] | 0.799 | |  |  |
| Sex * APOE4 homozygous | -2.84 [-9.64, 3.97] | 0.414 | |  |  |
| Average acceleration * Sex * APOE4 heterozygous | -0.01 [-0.09, 0.08] | 0.904 | |  |  |
| Average acceleration * Sex * APOE4 homozygous | 0.13 [-0.10, 0.36] | 0.284 | |  |  |
| Bipolar |  |  | | 0.47 [-1.88, 2.82] | 0.694 |
| Recurrent depression |  |  | | 0.13 [-0.41, 0.66] | 0.642 |
| Ethnicity Asian |  |  | | -2.42 [-3.65, -1.18] | **<0.001** |
| Ethnicity Black |  |  | | -1.87 [-3.89, 0.15] | 0.069 |
| Ethnicity Mixed |  |  | | -1.22 [-2.48, 0.05] | 0.060 |
| Has smoked |  |  | | 0.24 [-0.01, 0.49] | 0.062 |
| Secondary education |  |  | | 1.39 [0.58, 2.19] | **0.001** |
| Higher education |  |  | | 1.66 [0.90, 2.41] | **<0.001** |
| CVD diagnosis |  |  | | -0.05 [-0.32, 0.21] | 0.702 |
| Rare alcohol intake |  |  | | 0.10 [-0.46, 0.67] | 0.720 |
| Frequent alcohol intake |  |  | | 0.33 [-0.20, 0.85] | 0.221 |
| Sleep less than 7 hours |  |  | | 0.02 [-0.27, 0.31] | 0.896 |
| Sleep more than 9 hours |  |  | | 0.08 [-1.02, 1.19] | 0.881 |
| Diabetes diagnosis |  |  | | -0.02 [-0.60, 0.56] | 0.951 |
| BMI obese |  |  | | -0.49 [-0.87, -0.11] | **0.011** |
| BMI overweight |  |  | | -0.02 [-0.29, 0.26] | 0.912 |
| BMI severe obese |  |  | | -2.18 [-3.41, -0.96] | **<0.001** |
| BMI underweight |  |  | | -0.81 [-2.12, 0.51] | 0.230 |
| Age |  |  | | -0.06 [-0.08, -0.04] | **<0.001** |

NB: Model one was significantly able to predict % correct scores on the numeric memory task, F(11,10095)= 2.93, p<0.001. It accounted for 0.21% of the total variance (R^2^).

Model two was also significantly able to predict % correct scores on the numeric memory task, F(23, 10083) = 6.41, p<0.001. It accounted for 1.22% (R^2^) of the total variance, with an R^2^ change value of 1.01% compared to the previous model.

Supplementary Table 19. Results from the linear regression (Box cox transformation) where % correct on the numeric memory task was modelled by average acceleration.

|  | Model one | | Model two | |
| --- | --- | --- | --- | --- |
|  | Estimate | p | Estimate | p |
| Average acceleration | 1.98 [-0.32, 4.27] | 0.092 | -0.45 [-1.90, 1.00] | 0.543 |
| Sex | 34.49 [-58.51, 127.49] | 0.467 | 47.42 [27.31, 67.52] | **<0.001** |
| APOE4 heterozygous | 85.03 [-45.85, 223.91] | 0.196 | 5.94 [-16.95, 28.84] | 0.611 |
| APOE4 homozygous | 233.74 [-147.71, 615.19] | 0.230 | 53.93 [-11.75, 119.61] | 0.108 |
| Average acceleration*Sex | 0.21 [-2.97, 3.40] | 0.896 |  |  |
| Average acceleration*APOE4 heterozygous | -3.03 [-7.51, 1.44] | 0.184 |  |  |
| Average acceleration* APOE4 homozygous | -7.03 [-19.66, 5.61] | 0.276 |  |  |
| Sex * APOE4 heterozygous | 20.30 [-172.21, 212.80] | 0.836 |  |  |
| Sex * APOE4 homozygous | -257.61 [-796.56, 281.35] | 0.349 |  |  |
| Average acceleration * Sex * APOE4 heterozygous | -0.24 [-6.79, 6.30] | 0.942 |  |  |
| Average acceleration * Sex * APOE4 homozygous | 11.22 [-7.10, 29.53] | 0.230 |  |  |
| Bipolar |  |  | 39.21 [-146.81, 225.24] | 0.679 |
| Recurrent depression |  |  | 10.74 [-31.81, 53.29] | 0.621 |
| Ethnicity Asian |  |  | -188.03 [-285.91, -90.14] | **<0.001** |
| Ethnicity Black |  |  | -155.29 [-315.07, 4.48] | 0.057 |
| Ethnicity Mixed |  |  | -91.04 [-191.26, 9.18] | 0.075 |
| Has smoked |  |  | 20.22 [0.29, 40.15] | **0.047** |
| Secondary education |  |  | 107.81 [43.97, 171.65] | **0.001** |
| Higher education |  |  | 129.10 [69.21, 188.99] | **<0.001** |
| CVD diagnosis |  |  | -4.06 [-25.20, 17.07] | 0.706 |
| Rare alcohol intake |  |  | 7.05 [-37.55, 51.64] | 0.757 |
| Frequent alcohol intake |  |  | 24.78 [-16.75, 66.31] | 0.242 |
| Sleep less than 7 hours |  |  | 2.91 [-20.05, 25.87] | 0.804 |
| Sleep more than 9 hours |  |  | 8.82 [-78.49, 96.13] | 0.843 |
| Diabetes diagnosis |  |  | -2.20 [-47.93, 43.54] | 0.925 |
| BMI obese |  |  | -39.31 [-69.14, -9.48] | **0.010** |
| BMI overweight |  |  | -0.78 [-22.62, 21.06] | 0.944 |
| BMI severe obese |  |  | -167.65 [-264.45, -70.86] | **0.001** |
| BMI underweight |  |  | -70.54 [-174.73, 33.66] | 0.185 |
| Age |  |  | -4.85 [-6.24, -3.46] | **<0.001** |

NB: Model one could predict % correct on the numeric memory task, F(11, 10095) = 3.04, p<0.001. It accounted for 0.22% of the total variance (R^2^).

Model two could also significantly predict % correct on the numeric memory task, F(23, 10083) = 6.46, p<0.001. It accounted for 1.22% of the total variance (R^2^), with an R^2^change value of 1.00%.

Supplementary Table 20. Results from the linear regression where % correct on the numeric memory task was modelled by IPAQ group.

|  | Model one | | | Model two | |
| --- | --- | --- | --- | --- | --- |
|  | Estimate | p | Estimate | | p |
| IPAQ high | -0.43 [-0.99, 0.14] | 0.136 | -0.30 [-0.64, 0.05] | | 0.091 |
| IPAQ moderate | -0.59 [-1.13, -0.04] | **0.035** | -0.15 [-0.49, 0.18] | | 0.377 |
| Sex | 0.11 [-0.54, 0.75] | 0.742 | 0.58 [0.33, 0.84] | | **<0.001** |
| APOE4 heterozygous | -0.74 [-1.68, 0.20] | 0.123 | 0.07 [-0.22, 0.36] | | 0.653 |
| APOE4 homozygous | -0.08 [-2.73, 2.57] | 0.955 | 0.68 [-0.15, 1.51] | | 0.110 |
| IPAQ high*Sex | 0.23 [-0.57, 1.02] | 0.557 |  | |  |
| IPAQ moderate*Sex | 0.63 [-0.15, 1.40] | 0.113 |  | |  |
| IPAQ high * APOE4 heterozygous | 0.66 [-0.51, 1.77] | 0.297 |  | |  |
| IPAQ moderate * APOE4 heterozygous | 1.13 [0.02, 2.25] | **0.046** |  | |  |
| IPAQ high * APOE4 homozygous | -0.89 [-4.15, 2.37] | 0.592 |  | |  |
| IPAQ moderate * APOE4 homozygous | 1.79 [-1.39, 4.96] | 0.271 |  | |  |
| Sex * APOE4 heterozygous | 0.83 [-0.52, 2.18] | 0.227 |  | |  |
| Sex * APOE4 homozygous | 1.53 [-2.02, 5.07] | 0.399 |  | |  |
| IPAQ high * Sex * APOE4 heterozygous | -0.67 [-2.31, 0.97] | 0.423 |  | |  |
| IPAQ moderate * Sex * APOE4 heterozygous | -0.78 [-2.40, 0.84] | 0.346 |  | |  |
| IPAQ high * Sex * APOE4 homozygous | 0.25 [-4.21, 4.72] | 0.911 |  | |  |
| IPAQ moderate * Sex * APOE4 homozygous | -1.92 [-6.34, 2.51] | 0.396 |  | |  |
| Bipolar |  |  | 0.48 [-1.87, 2.83] | | 0.690 |
| Recurrent depression |  |  | 0.14 [-0.40, 0.67] | | 0.619 |
| Ethnicity Asian |  |  | -2.40 [-3.64, -1.16] | | **<0.001** |
| Ethnicity Black |  |  | -1.85 [-3.87, 0.17] | | 0.072 |
| Ethnicity Mixed |  |  | -1.20 [-2.46, 0.07] | | 0.064 |
| Has smoked |  |  | 0.24 [-0.01, 0.49] | | 0.064 |
| Secondary education |  |  | 1.39 [0.58, 2.19] | | **0.001** |
| Higher education |  |  | 1.66 [0.90, 2.41] | | **<0.001** |
| CVD diagnosis |  |  | -0.05 [-0.32, 0.22] | | 0.713 |
| Rare alcohol intake |  |  | 0.11 [-0.46, 0.67] | | 0.710 |
| Frequent alcohol intake |  |  | 0.33 [-0.20, 0.85] | | 0.222 |
| Sleep less than 7 hours |  |  | 0.02 [-0.27, 0.31] | | 0.903 |
| Sleep more than 9 hours |  |  | 0.08 [-1.02, 1.18] | | 0.884 |
| Diabetes diagnosis |  |  | -0.03 [-0.60, 0.55] | | 0.927 |
| BMI obese |  |  | -0.49 [-0.86, -0.12] | | **0.010** |
| BMI overweight |  |  | -0.01 [-0.29, 0.26] | | 0.929 |
| BMI severe obese |  |  | -2.20 [-3.41, -0.98] | | **0.001** |
| BMI underweight |  |  | -0.82 [-2.14, 0.49] | | 0.220 |
| Age |  |  | -0.06 [-0.08, -0.04] | | **<0.001** |

NB: Model one was significantly able to predict % correct score on the numeric memory task, F(17, 10089)= 2.17, p=0.003. It accounted for 0.70% of the total variance (R^2^).

Model two was also significantly able to predict % correct score on the numeric memory task, F(24, 10082) = 6.25, p<0.001. It accounted for 1.23% of the total variance (R^2^), with an R^2^ change value of 0.53%.

Supplementary Table 21. Results from the linear regression (Box Cox transformation) where % correct on the numeric memory task was modelled by IPAQ group.

|  | Model one | | | Model two | |
| --- | --- | --- | --- | --- | --- |
|  | Estimate | p | Estimate | | p |
| IPAQ high | -37.70 [-82.34, 6.94] | 0.098 | -26.83 [-54.02, 0.36] | | 0.053 |
| IPAQ moderate | -48.79 [-91.96, -5.63] | **0.027** | -14.31 [-40.93, 12.30] | | 0.292 |
| Sex | 11.01 [-40.10, 62.11] | 0.673 | 47.69 [27.58, 67.80] | | **<0.001** |
| APOE4 heterozygous | -59.01 [-133.58, 15.56] | 0.121 | 6.17 [-16.72, 29.06] | | 0.597 |
| APOE4 homozygous | -10.36 [-220.17, 199.44] | 0.923 | 53.46 [-12.22, 119.14] | | 0.111 |
| IPAQ high*Sex | 16.63 [-46.11, 79.37] | 0.603 |  | |  |
| IPAQ moderate*Sex | 48.58 [-12.78, 109.94] | 0.121 |  | |  |
| IPAQ high * APOE4 heterozygous | 53.10 [-37.21, 143.41] | 0.249 |  | |  |
| IPAQ moderate * APOE4 heterozygous | 90.33 [1.96, 178.71] | **0.045** |  | |  |
| IPAQ high * APOE4 homozygous | -68.53 [-326.45, 189.39] | 0.602 |  | |  |
| IPAQ moderate * APOE4 homozygous | 147.48 [-104.18, 399.14] | 0.251 |  | |  |
| Sex * APOE4 heterozygous | 63.99 [-43.00, 170.97] | 0.241 |  | |  |
| Sex * APOE4 homozygous | 120.06 [-160.73, 400.84] | 0.402 |  | |  |
| IPAQ high * Sex * APOE4 heterozygous | -51.95 [-181.88, 77.98] | 0.433 |  | |  |
| IPAQ moderate * Sex * APOE4 heterozygous | -59.50 [-187.68, 68.89] | 0.363 |  | |  |
| IPAQ high * Sex * APOE4 homozygous | 25.43 [-328.00, 278.86] | 0.888 |  | |  |
| IPAQ moderate * Sex * APOE4 homozygous | -150.14 [-500.65, 200.36] | 0.401 |  | |  |
| Bipolar |  |  | 39.89 [-146.11, 225.89] | | 0.674 |
| Recurrent depression |  |  | 11.50 [-31.04, 54.04] | | 0.596 |
| Ethnicity Asian |  |  | -186.58 [-284.47, -88.70] | | **<0.001** |
| Ethnicity Black |  |  | -153.71 [-313.48, 6.05] | | 0.059 |
| Ethnicity Mixed |  |  | -89.38 [-189.61, 10.84] | | 0.080 |
| Has smoked |  |  | 20.05 [0.13, 39.97] | | **0.049** |
| Secondary education |  |  | 107.63 [43.81, 171.46] | | **0.001** |
| Higher education |  |  | 129.29 [69.41, 189.18] | | **<0.001** |
| CVD diagnosis |  |  | -3.91 [-25.03, 17.20] | | 0.716 |
| Rare alcohol intake |  |  | 7.32 [-37.25, 51.90] | | 0.747 |
| Frequent alcohol intake |  |  | 24.72 [-16.81, 66.25] | | 0.243 |
| Sleep less than 7 hours |  |  | 2.80 [-20.16, 25.76] | | 0.811 |
| Sleep more than 9 hours |  |  | 8.45 [-78.85, 95.74] | | 0.850 |
| Diabetes diagnosis |  |  | -3.18 [-48.86, 42.50] | | 0.891 |
| BMI obese |  |  | -39.84 [-69.22, -10.46] | | 0.080 |
| BMI overweight |  |  | -0.64 [-22.34, 21.05] | | 0.954 |
| BMI severe obese |  |  | -169.32 [-265.64, -73.00] | | **0.001** |
| BMI underweight |  |  | -71.94 [-176.10, 32.21] | | 0.176 |
| Age |  |  | -4.71 [-6.07, -3.36] | | **<0.001** |

NB: Model one could significantly predict % correct on the numeric memory task, F(17, 10089) = 2.31, p=0.002. It accounted for 0.21% of the total variance (R^2^).

Model two could significantly predict % correct on the numeric memory task, F(24, 10082) = 6.36, p<0.001. It accounted for 1.25% of the total variance (R^2^), with an R^2^ change value of 1.04% compared to the previous model.

Supplementary Table 22. Results from the linear regression where mean time to correctly identify matches was modelled by average acceleration.

|  | Model one | | Model two | |
| --- | --- | --- | --- | --- |
|  | Estimate | p | Estimate | p |
| Average acceleration | -1.78 [-2.19, -1.37] | **<0.001** | -0.43 [-0.68, -0.18] | **0.001** |
| Sex | -32.94 [-49.65, -16.24] | **<0.001** | -23.02 [-26.48, -19.56] | **<0.001** |
| APOE4 heterozygous | -29.75 [-53.98, -5.52] | **0.016** | -2.87 [-6.81, 1.07] | 0.153 |
| APOE4 homozygous | -61.25 [-129.78, 7.27] | 0.080 | 3.81 [-7.49, 15.10] | 0.509 |
| Average acceleration*Sex | 0.53 [-0.04, 1.10] | 0.069 |  |  |
| Average acceleration*APOE4 heterozygous | 0.94 [0.14, 1.75] | **0.021** |  |  |
| Average acceleration* APOE4 homozygous | 1.85 [-0.42, 4.12] | 0.110 |  |  |
| Sex * APOE4 heterozygous | 0.57 [-34.01, 35.15] | 0.974 |  |  |
| Sex * APOE4 homozygous | 66.36 [-30.45, 163.18] | 0.179 |  |  |
| Average acceleration * Sex * APOE4 heterozygous | -0.15 [-1.32, 1.03] | 0.804 |  |  |
| Average acceleration * Sex * APOE4 homozygous | -1.70 [-4.99, 1.59] | 0.310 |  |  |
| Bipolar |  |  | -13.10 [-45.09, 18.90] | 0.422 |
| Recurrent depression |  |  | -5.94 [-13.26, 1.38] | 0.112 |
| Ethnicity Asian |  |  | 23.90 [7.06, 40.73] | **0.005** |
| Ethnicity Black |  |  | 55.05 [27.57, 82.53] | **<0.001** |
| Ethnicity Mixed |  |  | 17.57 [0.33, 34.80] | **0.046** |
| Has smoked |  |  | 0.93 [-2.50, 4.35] | 0.596 |
| Secondary education |  |  | -15.40 [-26.38, -4.42] | **0.006** |
| Higher education |  |  | -23.28 [-33.58, -12.98] | **<0.001** |
| CVD diagnosis |  |  | -0.41 [-4.05, 3.22] | 0.825 |
| Rare alcohol intake |  |  | 4.81 [-2.86, 12.48] | 0.219 |
| Frequent alcohol intake |  |  | 1.25 [-5.89, 8.39] | 0.731 |
| Sleep less than 7 hours |  |  | -1.85 [-5.79, 2.10] | 0.360 |
| Sleep more than 9 hours |  |  | 6.90 [-8.12, 21.92] | 0.368 |
| Diabetes diagnosis |  |  | 2.78 [-5.09, 10.64] | 0.489 |
| BMI obese |  |  | 3.62 [-1.51, 8.75] | 0.167 |
| BMI overweight |  |  | 0.26 [-3.50, 4.01] | 0.894 |
| BMI severe obese |  |  | -7.56 [-24.21, 9.09] | 0.373 |
| BMI underweight |  |  | 19.70 [1.78, 37.62] | **0.031** |
| Age |  |  | 3.59 [3.35, 3.83] | **<0.001** |

NB: Model one was significantly able to predict the mean reaction time, F(11,10095)= 19.87, p<0.001. It accounted for 2.01% of the total variance (R^2^).

Model two was also significantly able to predict the mean reaction time, F(23, 10083) = 55.76, p<0.001. It account for 11.08% of the total variance (R^2^), and had an R^2^ change value of 9.07% compared to the previous model.

Supplementary Table 23. Results from the linear regression where mean time to correctly identify matches was modelled by IPAQ group.

|  | Model one | | | Model two | |
| --- | --- | --- | --- | --- | --- |
|  | Estimate | p | Estimate | | p |
| IPAQ high | -1.15 [-9.22, 6.92] | 0.780 | -1.51 [-6.18, 3.17] | | 0.528 |
| IPAQ moderate | -4.53 [-12.33, 3.27] | 0.255 | -0.22 [-4.80, 4.36] | | 0.926 |
| Sex | -21.84 [-31.08, -12.61] | <**0.001** | -22.82 [-26.28, -19.36] | | **<0.001** |
| APOE4 heterozygous | -6.47 [-19.95, 7.00] | 0.346 | -2.95 [-6.89, 0.99] | | 0.142 |
| APOE4 homozygous | -8.33 [-46.24, 29.57] | 0.667 | 3.84 [-7.47, 15.14] | | 0.506 |
| IPAQ high*Sex | 3.76 [-7.57, 15.10] | 0.515 |  | |  |
| IPAQ moderate*Sex | 8.89 [-2.19, 19.98] | 0.116 |  | |  |
| IPAQ high * APOE4 heterozygous | 2.60 [-13.72, 18.91] | 0.755 |  | |  |
| IPAQ moderate * APOE4 heterozygous | 5.14 [-10.83, 21.11] | 0.528 |  | |  |
| IPAQ high * APOE4 homozygous | -10.71 [-57.31, 35.88] | 0.652 |  | |  |
| IPAQ moderate * APOE4 homozygous | 9.75 [-35.71, 55.22] | 0.674 |  | |  |
| Sex * APOE4 heterozygous | -0.12 [-19.45, 19.21] | 0.990 |  | |  |
| Sex * APOE4 homozygous | 33.92 [-16.81, 84.65] | 0.190 |  | |  |
| IPAQ high * Sex * APOE4 heterozygous | -4.46 [-27.83, 19.12] | 0.716 |  | |  |
| IPAQ moderate * Sex * APOE4 heterozygous | -4.60 [-27.76, 18.56] | 0.697 |  | |  |
| IPAQ high * Sex * APOE4 homozygous | 1.79 [-62.06, 65.65] | 0.956 |  | |  |
| IPAQ moderate * Sex * APOE4 homozygous | -45.08 [-108.41, 18.24] | 0.163 |  | |  |
| Bipolar |  |  | -13.54 [-45.55, 18.48] | | 0.407 |
| Recurrent depression |  |  | -5.59 [-12.92, 1.73] | | 0.134 |
| Ethnicity Asian |  |  | 24.10 [7.25, 40.94] | | **0.005** |
| Ethnicity Black |  |  | 55.16 [27.66, 82.66] | | **<0.001** |
| Ethnicity Mixed |  |  | 18.04 [0.79, 35.29] | | **0.040** |
| Has smoked |  |  | 0.84 [-2.59, 4.27] | | 0.630 |
| Secondary education |  |  | -15.09 [-26.08, -4.11] | | **0.007** |
| Higher education |  |  | -22.90 [-33.21, -12.60] | | **<0.001** |
| CVD diagnosis |  |  | -0.18 [-3.82, 3.45] | | 0.921 |
| Rare alcohol intake |  |  | 5.17 [-2.50, 12.85] | | 0.186 |
| Frequent alcohol intake |  |  | 1.16 [-5.99, 8.31] | | 0.751 |
| Sleep less than 7 hours |  |  | -1.92 [-5.87, 2.03] | | 0.340 |
| Sleep more than 9 hours |  |  | 7.55 [-7.47, 22.58] | | 0.325 |
| Diabetes diagnosis |  |  | 3.55[-4.31, 11.41] | | 0.376 |
| BMI obese |  |  | 5.18 [0.13, 10.24] | | **0.044** |
| BMI overweight |  |  | 1.01 [-2.72, 4.74] | | 0.596 |
| BMI severe obese |  |  | -4.59 [-21.16, 11.99] | | 0.558 |
| BMI underweight |  |  | 18.91 [0.99, 36.84] | | **0.039** |
| Age |  |  | 3.68 [3.45, 3.91] | | **<0.001** |

NB: Model one was significantly able to predict mean reaction time, F(17, 10089) = 6.28, p<0.001. It accounted for 0.88% of the total variance (R^2^).

Model two was also significantly able to predict mean reaction time, F(24, 10082) =52.91, p<0.001. It accounted for 10.98% of the total variance (R^2^), with an R^2^ change value of 10.10% compared to the previous model.

Supplementary Table 24. Results from the linear regression where % correct on the pairs matching task was modelled by average acceleration.

|  | Model one | | Model two | |
| --- | --- | --- | --- | --- |
|  | Estimate | p | Estimate | p |
| Average acceleration | 0.00 [-0.05, 0.05] | 0.998 | -0.06 [-0.09, -0.03] | **<0.001** |
| Sex | -1.06 [-3.20, 1.08] | 0.331 | 0.53 [0.08, 0.99] | **0.022** |
| APOE4 heterozygous | 1.47 [-1.64, 4.57] | 0.354 | -0.43 [-0.95, 0.09] | 0.104 |
| APOE4 homozygous | -5.40 [-14.18, 3.38] | 0.228 | -0.39 [-1.88, 1.10] | 0.607 |
| Average acceleration*Sex | 0.04 [-0.04, 0.11] | 0.338 |  |  |
| Average acceleration*APOE4 heterozygous | -0.08 [-0.18, 0.03] | 0.151 |  |  |
| Average acceleration* APOE4 homozygous | 0.21 [-0.08, 0.50] | 0.162 |  |  |
| Sex * APOE4 heterozygous | -0.61 [-5.04, 3.82] | 0.786 |  |  |
| Sex * APOE4 homozygous | 7.37 [-5.03, 19.78] | 0.244 |  |  |
| Average acceleration * Sex * APOE4 heterozygous | 0.05 [-0.10, 0.20] | 0.505 |  |  |
| Average acceleration * Sex * APOE4 homozygous | -0.31 [-0.73, 0.11] | 0.146 |  |  |
| Bipolar |  |  | -1.26 [-5.48, 2.96] | 0.558 |
| Recurrent depression |  |  | -0.33 [-1.29, 0.64] | 0.506 |
| Ethnicity Asian |  |  | -3.65 [-5.87, -1.43] | **0.001** |
| Ethnicity Black |  |  | -7.03 [-10.66, -3.41] | **<0.001** |
| Ethnicity Mixed |  |  | -2.59 [-4.86, -0.32] | **0.026** |
| Has smoked |  |  | -0.24 [-0.69, 0.21] | 0.304 |
| Secondary education |  |  | 1.77 [0.32, 3.22] | **0.017** |
| Higher education |  |  | 2.53 [1.18, 3.89] | **<0.001** |
| CVD diagnosis |  |  | -0.17 [-0.65, 0.31] | 0.483 |
| Rare alcohol intake |  |  | 0.82 [-0.19, 1.83] | 0.113 |
| Frequent alcohol intake |  |  | 0.44 [-0.51, 1.38] | 0.364 |
| Sleep less than 7 hours |  |  | -0.05 [-0.57, 0.47] | 0.861 |
| Sleep more than 9 hours |  |  | -1.05 [-3.03, 0.93] | 0.300 |
| Diabetes diagnosis |  |  | -0.90 [-1.94, 0.14] | 0.089 |
| BMI obese |  |  | 0.16 [-0.52, 0.83] | 0.652 |
| BMI overweight |  |  | 0.03 [-0.47, 0.53] | 0.906 |
| BMI severe obese |  |  | -0.45 [-2.65, 1.75] | 0.687 |
| BMI underweight |  |  | -2.66 [-5.03, -0.30] | **0.027** |
| Age |  |  | -0.28 [-0.31, -0.25] | **<0.001** |

NB: Model one was not significantly able to predict % correct scores on the pairs matching task, F(11, 10095)= 1.19, p>0.05.

Model two was significantly able to predict % correct scores on the pairs matching task, F(23, 10083) = 18.29, p<0.001. It accounted for 3.79% of the total variance (R^2^).

Supplementary Table 25. Results from the linear regression (Yeo-Johnson transformation) where % correct on the pairs matching task was modelled by average acceleration.

|  | Model one | | Model two | |  |
| --- | --- | --- | --- | --- | --- |
|  | Estimate | p | Estimate | p | |
| Average acceleration | 0.00 [0.00, 0.00] | 0.887 | 0.00 [0.00, 0.00] | **0.001** | |
| Sex | -0.01 [-0.04, 0.02] | 0.384 | 0.01 [0.00, 0.01] | **0.012** | |
| APOE4 heterozygous | 0.02 [-0.03, 0.06] | 0.480 | -0.01 [-0.01, 0.00] | 0.107 | |
| APOE4 homozygous | -0.06 [-0.19, 0.06] | 0.302 | -0.01 [-0.03, 0.01] | 0.553 | |
| Average acceleration*Sex | 0.00 [0.00, 0.00] | 0.357 |  |  | |
| Average acceleration*APOE4 heterozygous | 0.00 [0.00, 0.00] | 0.230 |  |  | |
| Average acceleration* APOE4 homozygous | 0.00 [0.00, 0.01] | 0.214 |  |  | |
| Sex * APOE4 heterozygous | -0.01 [-0.07, 0.06] | 0.868 |  |  | |
| Sex * APOE4 homozygous | 0.08 [-0.09, 0.25] | 0.360 |  |  | |
| Average acceleration * Sex * APOE4 heterozygous | 0.00 [0.00, 0.00] | 0.589 |  |  | |
| Average acceleration * Sex * APOE4 homozygous | 0.00 [-0.01, 0.00] | 0.208 |  |  | |
| Bipolar |  |  | -0.01 [-0.07, 0.05] | 0.666 | |
| Recurrent depression |  |  | 0.00 [-0.02, 0.01] | 0.497 | |
| Ethnicity Asian |  |  | -0.05 [-0.08, -0.02] | **0.002** | |
| Ethnicity Black |  |  | -0.11 [-0.16, -0.06] | **<0.001** | |
| Ethnicity Mixed |  |  | -0.04 [-0.07, -0.01] | **0.009** | |
| Has smoked |  |  | 0.00 [-0.01, 0.00] | 0.247 | |
| Secondary education |  |  | 0.03 [0.01, 0.05] | **0.011** | |
| Higher education |  |  | 0.04 [0.02, 0.05] | **<0.001** | |
| CVD diagnosis |  |  | 0.00 [-0.01, 0.01] | 0.687 | |
| Rare alcohol intake |  |  | 0.01 [0.00, 0.02] | 0.139 | |
| Frequent alcohol intake |  |  | 0.00 [-0.01, 0.02] | 0.541 | |
| Sleep less than 7 hours |  |  | 0.00 [-0.01, 0.01] | 0.587 | |
| Sleep more than 9 hours |  |  | -0.01 [-0.04, 0.02] | 0.424 | |
| Diabetes diagnosis |  |  | -0.01 [-0.03, 0.00] | 0.055 | |
| BMI obese |  |  | 0.00 [-0.01, 0.01] | 0.740 | |
| BMI overweight |  |  | 0.00 [-0.01, 0.01] | 0.683 | |
| BMI severe obese |  |  | -0.01 [-0.04, 0.02] | 0.560 | |
| BMI underweight |  |  | -0.04 [-0.07, 0.00] | **0.032** | |
| Age |  |  | 0.00 [0.00, 0.00] | **<0.001** | |

NB: Model one was not significantly able to predict % correct scores on the pairs matching task, F(11, 10095) = 1.11, p>0.05.

Model two could significantly predict % correct scores on the pairs matching task, F(23, 10083) = 16.04, p<0.001. It had an R^2^ value of 3.31%.

Supplementary Table 26. Results from the linear regression where % correct on the pairs matching was modelled by IPAQ group.

|  | Model one | | | Model two | |
| --- | --- | --- | --- | --- | --- |
|  | Estimate | p | Estimate | | p |
| IPAQ high | -1.27 [-2.29, -0.24] | **0.016** | -0.34 [-0.95, 0.28] | | 0.285 |
| IPAQ moderate | -0.48 [-1.47, 0.51] | 0.342 | -0.09 [-0.69, 0.51] | | 0.771 |
| Sex | -0.21 [-1.39, 0.96] | 0.722 | 0.55 [0.11, 1.02] | | **0.016** |
| APOE4 heterozygous | -2.44 [-4.15, -0.72] | **0.005** | -0.44 [-0.96, 0.08] | | 0.097 |
| APOE4 homozygous | -2.17 [-7.00, 2.66] | 0.378 | -0.39 [-1.88, 1.10] | | 0.609 |
| IPAQ high*Sex | 0.54 [-0.90, 1.99] | 0.460 |  | |  |
| IPAQ moderate*Sex | -0.13 [-1.54, 1.29] | 0.861 |  | |  |
| IPAQ high * APOE4 heterozygous | 2.13 [0.05, 4.20] | **0.045** |  | |  |
| IPAQ moderate * APOE4 heterozygous | 2.01 [-0.02, 4.04] | 0.053 |  | |  |
| IPAQ high * APOE4 homozygous | 3.48 [-2.46, 9.41] | 0.251 |  | |  |
| IPAQ moderate * APOE4 homozygous | 3.57 [-2.22, 9.37] | 0.226 |  | |  |
| Sex * APOE4 heterozygous | 1.44 [-1.02, 3.90] | 0.252 |  | |  |
| Sex * APOE4 homozygous | 1.88 [-4.59, 8.34] | 0.569 |  | |  |
| IPAQ high * Sex * APOE4 heterozygous | -0.46 [-3.45, 2.53] | 0.762 |  | |  |
| IPAQ moderate * Sex * APOE4 heterozygous | -0.75 [-3.70, 2.20] | 0.619 |  | |  |
| IPAQ high * Sex * APOE4 homozygous | -4.64 [-12.78, 3.49] | 0.263 |  | |  |
| IPAQ moderate * Sex * APOE4 homozygous | -4.26 [-12.33, 3.80] | 0.300 |  | |  |
| Bipolar |  |  | -1.32 [-5.54, 2.91] | | 0.542 |
| Recurrent depression |  |  | -0.28 [-1.24, 0.69] | | 0.571 |
| Ethnicity Asian |  |  | -3.62 [-5.84, -1.39] | | **0.001** |
| Ethnicity Black |  |  | -7.01 [-10.64, -3.38] | | **<0.001** |
| Ethnicity Mixed |  |  | -2.52 [-4.79, -0.24] | | **0.030** |
| Has smoked |  |  | -0.25 [-0.70, 0.20] | | 0.281 |
| Secondary education |  |  | 1.81 [0.36, 3.26] | | **0.015** |
| Higher education |  |  | 2.58 [1.22, 3.94] | | **<0.001** |
| CVD diagnosis |  |  | -0.14 [-0.62, 0.34] | | 0.563 |
| Rare alcohol intake |  |  | 0.87 [-0.14, 1.88] | | 0.093 |
| Frequent alcohol intake |  |  | 0.42 [-0.52, 1.37] | | 0.379 |
| Sleep less than 7 hours |  |  | -0.06 [-0.58, 0.46] | | 0.831 |
| Sleep more than 9 hours |  |  | -0.97 [-2.95, 1.02] | | 0.340 |
| Diabetes diagnosis |  |  | -0.81 [-1.84, 0.23] | | 0.128 |
| BMI obese |  |  | 0.36 [-0.31, 1.02] | | 0.295 |
| BMI overweight |  |  | 0.13 [-0.36, 0.62] | | 0.609 |
| BMI severe obese |  |  | -0.07 [-2.26, 2.11] | | 0.948 |
| BMI underweight |  |  | -2.77 [-5.14, -0.41] | | **0.022** |
| Age |  |  | -0.27 [-0.30, -0.24] | | **<0.001** |

NB: Model one was not significantly able to predict % correct on the pairs matching task, F(17, 10089) = 1.21, p>0.05.

Model two was significantly able to predict % correct on the pairs matching task, F(24, 10082) = 17.06, p<0.001. It accounted for 3.68% of the total variance (R^2^).

Supplementary Table 27. Results from the linear regression (Yeo Johnson transformation) where % correct on the pairs matching task was modelled by IPAQ group.

|  | Model one | | | Model two | |
| --- | --- | --- | --- | --- | --- |
|  | Estimate | p | Estimate | | p |
| IPAQ high | -0.01 [-0.03, 0.00] | 0.133 | 0.00 [-0.01, 0.01] | | 0.627 |
| IPAQ moderate | 0.00 [-0.02, 0.01] | 0.794 | 0.00 [-0.01, 0.01] | | 0.968 |
| Sex | 0.00 [-0.01, 0.02] | 0.712 | 0.01 [0.00, 0.01] | | **0.009** |
| APOE4 heterozygous | -0.03 [-0.06, -0.01] | **0.011** | -0.01 [-0.01, 0.00] | | 0.098 |
| APOE4 homozygous | -0.02 [-0.09, 0.05] | 0.544 | -0.01 [-0.03, 0.01] | | 0.557 |
| IPAQ high*Sex | 0.00 [-0.02, 0.02] | 0.940 |  | |  |
| IPAQ moderate*Sex | -0.01 [-0.03, 0.01] | 0.466 |  | |  |
| IPAQ high * APOE4 heterozygous | 0.03 [0.00, 0.05] | 0.083 |  | |  |
| IPAQ moderate * APOE4 heterozygous | 0.03 [0.00, 0.05] | 0.079 |  | |  |
| IPAQ high * APOE4 homozygous | 0.04 [-0.04, 0.12] | 0.376 |  | |  |
| IPAQ moderate * APOE4 homozygous | 0.04 [-0.04, 0.12[ | 0.320 |  | |  |
| Sex * APOE4 heterozygous | 0.02 [-0.02, 0.05] | 0.361 |  | |  |
| Sex * APOE4 homozygous | 0.02 [-0.07, 0.11] | 0.674 |  | |  |
| IPAQ high * Sex * APOE4 heterozygous | 0.00 [-0.04, 0.04] | 0.962 |  | |  |
| IPAQ moderate * Sex * APOE4 heterozygous | -0.01 [-0.05. 0.03] | 0.724 |  | |  |
| IPAQ high * Sex * APOE4 homozygous | -0.06 [-0.17, 0.06] | 0.342 |  | |  |
| IPAQ moderate * Sex * APOE4 homozygous | -0.07 [-0.18, 0.05] | 0.239 |  | |  |
| Bipolar |  |  | -0.01 [-0.07, 0.05] | | 0.646 |
| Recurrent depression |  |  | 0.00 [-0.02, 0.01] | | 0.555 |
| Ethnicity Asian |  |  | -0.05 [-0.08, -0.02] | | **0.002** |
| Ethnicity Black |  |  | -0.11 [-0.16, -0.06] | | **<0.001** |
| Ethnicity Mixed |  |  | -0.04 [-0.07, -0.01] | | **0.011** |
| Has smoked |  |  | 0.00 [-0.01, 0.00] | | 0.230 |
| Secondary education |  |  | 0.03 [0.01, 0.05] | | **0.009** |
| Higher education |  |  | 0.04 [0.02, 0.05] | | **<0.001** |
| CVD diagnosis |  |  | 0.00 [-0.01, 0.01] | | 0.779 |
| Rare alcohol intake |  |  | 0.01 [0.00, 0.03] | | 0.116 |
| Frequent alcohol intake |  |  | 0.00 [-0.01, 0.02 | | 0.559 |
| Sleep less than 7 hours |  |  | 0.00 [-0.01, 0.01] | | 0.563 |
| Sleep more than 9 hours |  |  | -0.01 [-0.04, 0.02] | | 0.477 |
| Diabetes diagnosis |  |  | -0.01 [-0.03, 0.00] | | 0.086 |
| BMI obese |  |  | 0.00 [0.00, 0.01] | | 0.332 |
| BMI overweight |  |  | 0.00 [-0.01, 0.01] | | 0.990 |
| BMI severe obese |  |  | 0.00 [-0.03, 0.03] | | 0.819 |
| BMI underweight |  |  | -0.04 [-0.07, 0.00] | | **0.026** |
| Age |  |  | 0.00 [0.00, 0.00] | | **<0.001** |

NB: Model one was unable to significantly predict % correct on the pairs matching task, F(17, 10089) = 1.12, p>0.05.

Model two was significantly able to predict % correct score on the pairs matching task, F(24, 10082) = 14.89, p<0.001. It accounted for 3.19% of the total variance (R^2^).

**Post hoc ANOVA model statistics**

For AD incidence group:

The one-way ANOVA could significantly predict the relationship between IPAQ group and average acceleration, F(2, 69057) = 1560.00, *p*<.0001; all Tukey HSD post hoc tests, *p*<0.001.

For brain volume group:

The one-way ANOVA could significantly predict the relationship between IPAQ group and average acceleration, F(2, 13093) = 281.60, *p*<0.001; all Tukey HSD post hoc tests, *p*<0.001.

For cognition group:

The one-way ANOVA could significantly predict the relationship between IPAQ group and average acceleration, F(2, 10104) = 241.60, *p*<0.001; all Tukey HSD post hoc tests, *p*<0.001.
